# Fragment Merging, Growing, and Linking Identify New Trypanothione Reductase Inhibitors for Leishmaniasis

**DOI:** 10.1021/acs.jmedchem.3c01439

**Published:** 2024-01-02

**Authors:** Cécile Exertier, Alessandra Salerno, Lorenzo Antonelli, Annarita Fiorillo, Riccardo Ocello, Francesca Seghetti, Jessica Caciolla, Elisa Uliassi, Matteo Masetti, Eleonora Fiorentino, Stefania Orsini, Trentina Di Muccio, Andrea Ilari, Maria Laura Bolognesi

**Affiliations:** † Institute of Molecular Biology and Pathology (IBPM) of the National Research Council of Italy (CNR), c/o Department of Biochemical Sciences, Sapienza University of Rome, Piazzale A. Moro 5, Roma 00185, Italy; ‡ Department of Pharmacy and Biotechnology, Alma Mater Studiorum—University of Bologna, Via Belmeloro 6, Bologna 40126, Italy; § Department of Biochemical Sciences “A. Rossi Fanelli”, Sapienza University of Rome, Piazzale A. Moro 5, Roma 00185, Italy; ∥ Computational and Chemical Biology, Istituto Italiano di Tecnologia, via Morego 30, Genova 16163, Italy; ⊥ Department of Infectious Diseases, Istituto Superiore di Sanità, Viale Regina Elena 299, Roma 00161, Italy

## Abstract

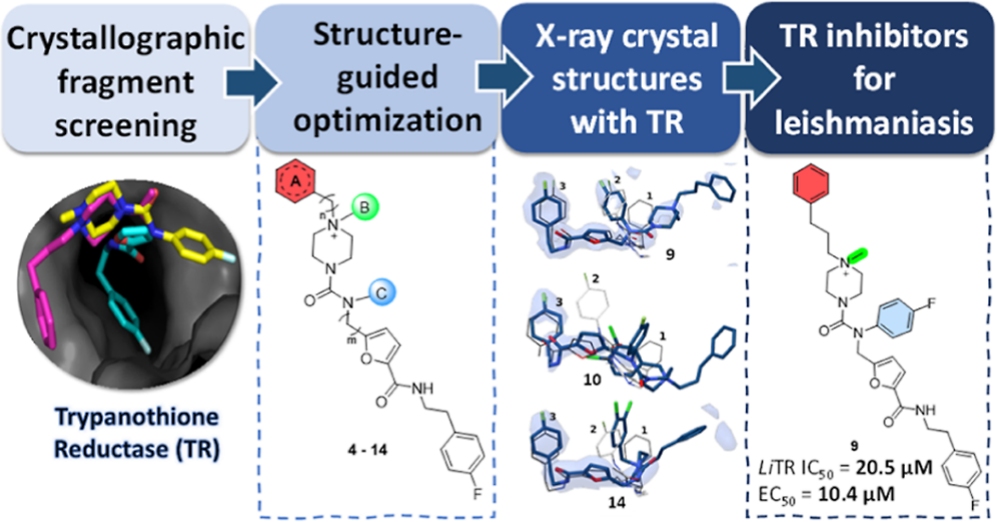

Trypanothione reductase (TR) is a suitable target for drug discovery approaches against leishmaniasis, although the identification of potent inhibitors is still challenging. Herein, we harnessed a fragment-based drug discovery (FBDD) strategy to develop new TR inhibitors. Previous crystallographic screening identified fragments **1**–**3**, which provided ideal starting points for a medicinal chemistry campaign. *In silico* investigations revealed critical hotspots in the TR binding site, guiding our structure- and ligand-based structure-actvity relationship (SAR) exploration that yielded fragment-derived compounds **4**–**14**. A trend of improvement in *Leishmania infantum* TR inhibition was detected along the optimization and confirmed by the crystal structures of **9**, **10**, and **14** in complex with *Trypanosoma brucei* TR. Compound **10** showed the best TR inhibitory profile (*K*_i_ = 0.2 μM), whereas **9** was the best one in terms of *in vitro* and *ex vivo* activity. Although further fine-tuning is needed to improve selectivity, we demonstrated the potentiality of FBDD on a classic but difficult target for leishmaniasis.

## Introduction

Leishmaniasis is a neglected zoonotic tropical disease transmitted by sandflies infected by protozoan parasites of the *Leishmania* genus. It has two main clinical forms: cutaneous leishmaniasis (CL) and visceral leishmaniasis (VL).^1^ CL leads to disfiguration with life-long scars that bring severe social stigma, particularly for women and children. VL—also known as kala-azar—causes fever, weight loss, spleen, and liver enlargement. Without proper diagnosis and treatment, VL can be deadly if left untreated.^1^

The disease is a significant public health concern in many parts of the world, especially in remote rural areas or conflict zones, where the poorest and most vulnerable populations live.^1^ However, environmental and climatic transformations (*i.e.*, deforestation and global warming) have induced the northward shifting of sandfly geographical distribution, spreading the disease to areas traditionally considered *Leishmania*-free.^1^ In Mediterranean regions, *Leishmania infantum* (*Li*) is the main cause of VL and CL.^2^

The latest estimates of leishmaniasis consist of one million new cases annually in 101 endemic countries.^1,3^ Recently, the World Health Organization (WHO) has identified the worldwide control of leishmaniasis and its elimination as priority targets.^4^ However, also because the less severe forms of leishmaniasis are not always fatal, the disease is receiving little attention from pharmaceutical companies, funding agencies, and local health systems.^5,6^

For all these reasons, new approaches for leishmaniasis drug discovery are urgently needed. To date, treatment opportunities are restricted to quite obsolete solutions (*i.e.*, pentavalent antimonials, amphotericin B, miltefosine, paromomycin) often endowed with heavy secondary effects, poor efficacy, and increasing parasite resistance.^7^ In the past decade, novel chemical entities, together with alternative therapeutic strategies, have been slowly populating the preclinical and clinical pipelines.^5^ Phenotypic drug discovery approaches still seem to stand out as the keystone, while target-based ones are poorly applied, due to the paucity of fully validated targets and the difficulty to relate leishmanicidal activity with on-target effects.^8^

**Figure 1:**
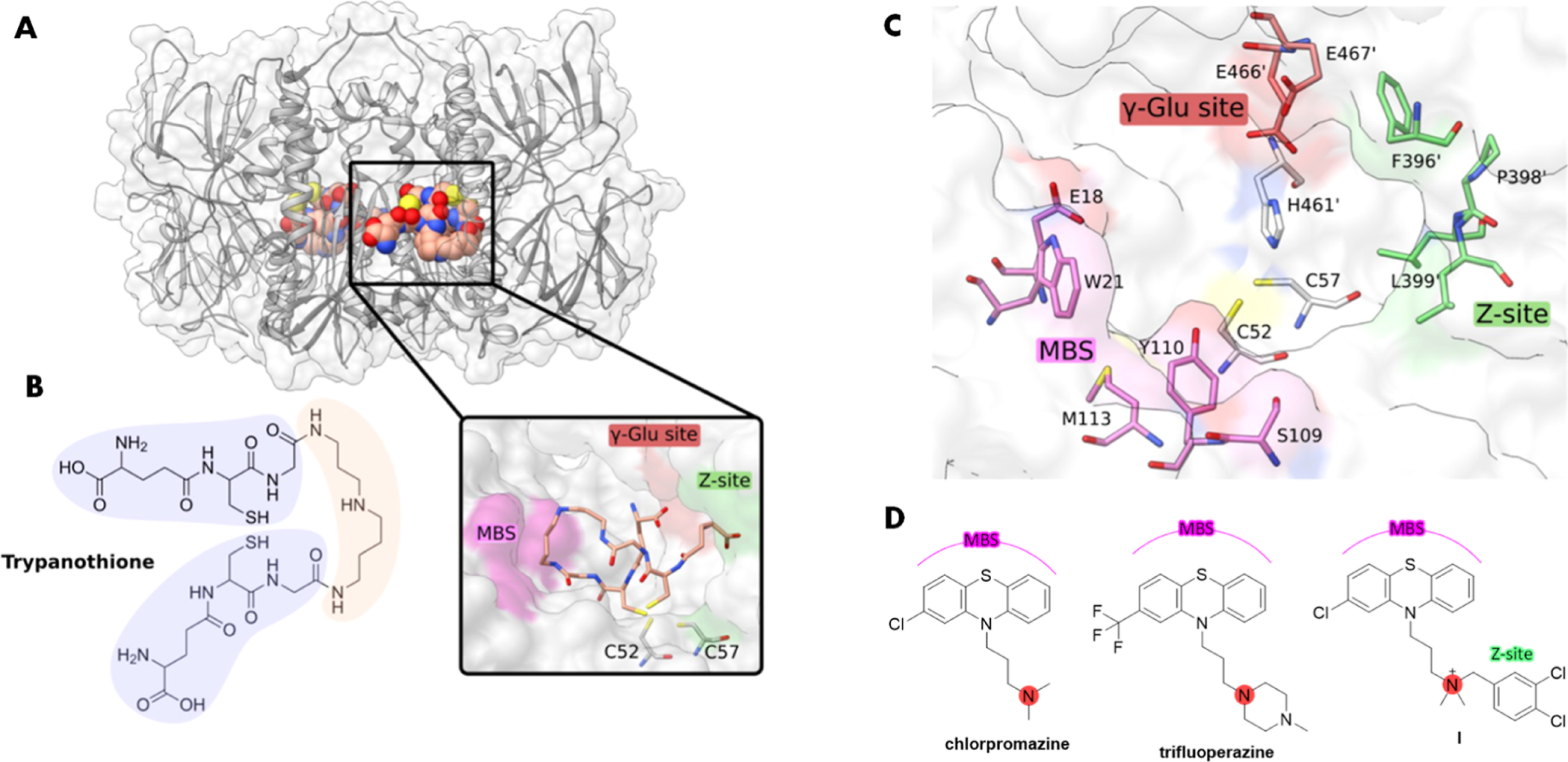
Structure of TR and of phenothiazine-based TR inhibitors. (A) Ribbon-and-stick representation of TR (PDB code 2WOW(20)). Trypanothione is displayed as spheres. A close-up view of the trypanothione close to the two catalytic cysteines is shown in the inset. (B) Trypanothione is composed of two glutathione moieties (light blue) joined by a spermidine (light red) linker *via* two amide bonds. (C) Representation of the trypanothione cavity. Residues belonging to the MBS are shown in magenta, and those belonging to the Z-site and to the γ-Glu site are displayed, respectively, in green and red. (D) Structure of phenothiazine-based TR inhibitors (chlorpromazine, trifluoperazine, and **I**(17)) targeting the MBS and Z-Site.

Among others, redox enzymes have been recognized as promising targets against *Leishmania*.^9^ Redox homeostasis plays a key role in parasite survival against the oxidative environment produced by the host macrophages. The redox defense of *Leishmania* mainly relies on trypanothione, which is kept in its reduced form by the activity of the trypanothione reductase (TR) enzyme (Figure 1A,B).^10^

TR has been pinpointed as a suitable target for several reasons: (i) the high level of genetic validation,^11,12^ (ii) the low risk for toxicity due to the differences in substrate specificity and structure compared to the homologue human glutathione reductase (*h*GR), (iii) assay feasibility, and (iv) detailed structural information.^13^

TR structure from different protozoan parasites has been extensively characterized by crystallography,^14^ showing that all key residues are conserved.^15^ This homodimeric flavoenzyme possesses four cavities binding two nicotinamide adenine dinucleotide phosphate hydrogen (NADPH) and two trypanothione molecules. Each NADPH cavity is separated from the trypanothione active site by a flavin adenine dinucleotide (FAD) cofactor allowing the transfer of two electrons by the participation of two catalytic cysteines (C52 and C57) which, together with H461′, E466′, and E467′ of the γ-Glu site (Figure 1C), form the catalytic machinery.

The active site features a hydrophobic cleft, named mepacrine binding site (MBS) after the crystal structure with the mepacrine inhibitor was solved.^16^ The MBS contains four residues, E18, W21, S109, and M113, where the negatively charged E18 is involved in binding with the trypanothione positive charges (Figure 1C). As these residues are not conserved in *h*GR, most of the TR inhibitors, especially those which were phenothiazine-based (*e.g*., chlorpromazine and trifluoperazine, Figure 1D), were designed to target the MBS.^10−15^

Nearby the MBS lies an additional hydrophobic subpocket, namely, the Z-site, mainly formed by residues F396′, P398′, and L399′ (Figure 1C).^17,18^ Targeting the Z-site proved to be a strategy for developing stronger TR inhibitors. For instance, compound **I** (Figure 1D) was developed by enlisting the Z-site in addition to the MBS and vectoring the inhibitor’s interaction by means of a third electrostatic site.^17^ This “three-point attachment” was realized upon the introduction of a *N*-3,4-dichlorobenzyl substituent on chlorpromazine, leading to a 2-order-of-magnitude-increase in TR inhibitory potency.^17^ Moreover, in a recent work, Ilari and co-workers^19^ reported a class of inhibitors targeting the Z-site and endowed with high activity and selectivity for TR.

Although TR can be claimed as a suitable target, its druggability is hampered by the large, featureless, and solvent-exposed trypanothione binding site and by a fast enzyme cellular turnover.^13,21^ As a consequence, competitive TR inhibitors developed so far have demonstrated low potency [kinetic inhibition constants (*K*_i_) in the micromolar range], and none of them have proceeded to the clinics.^14^ This makes the identification of high-affinity molecules still challenging.^14^

**Figure 2:**
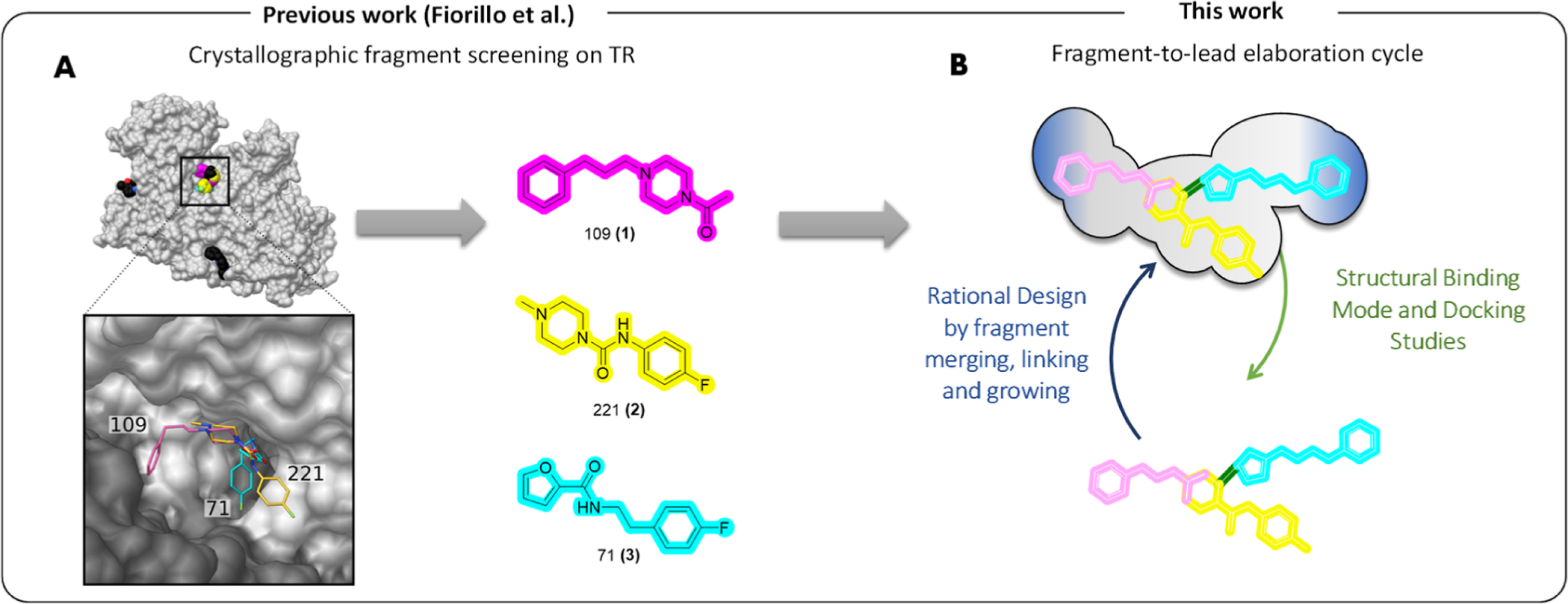
(A) Fragment screening reported in^18^ led to the identification of fragments **1**–**3** binding the Z-site of TR; (B) FBDD and fragment optimization workflow applied herein.

**Figure 3:**
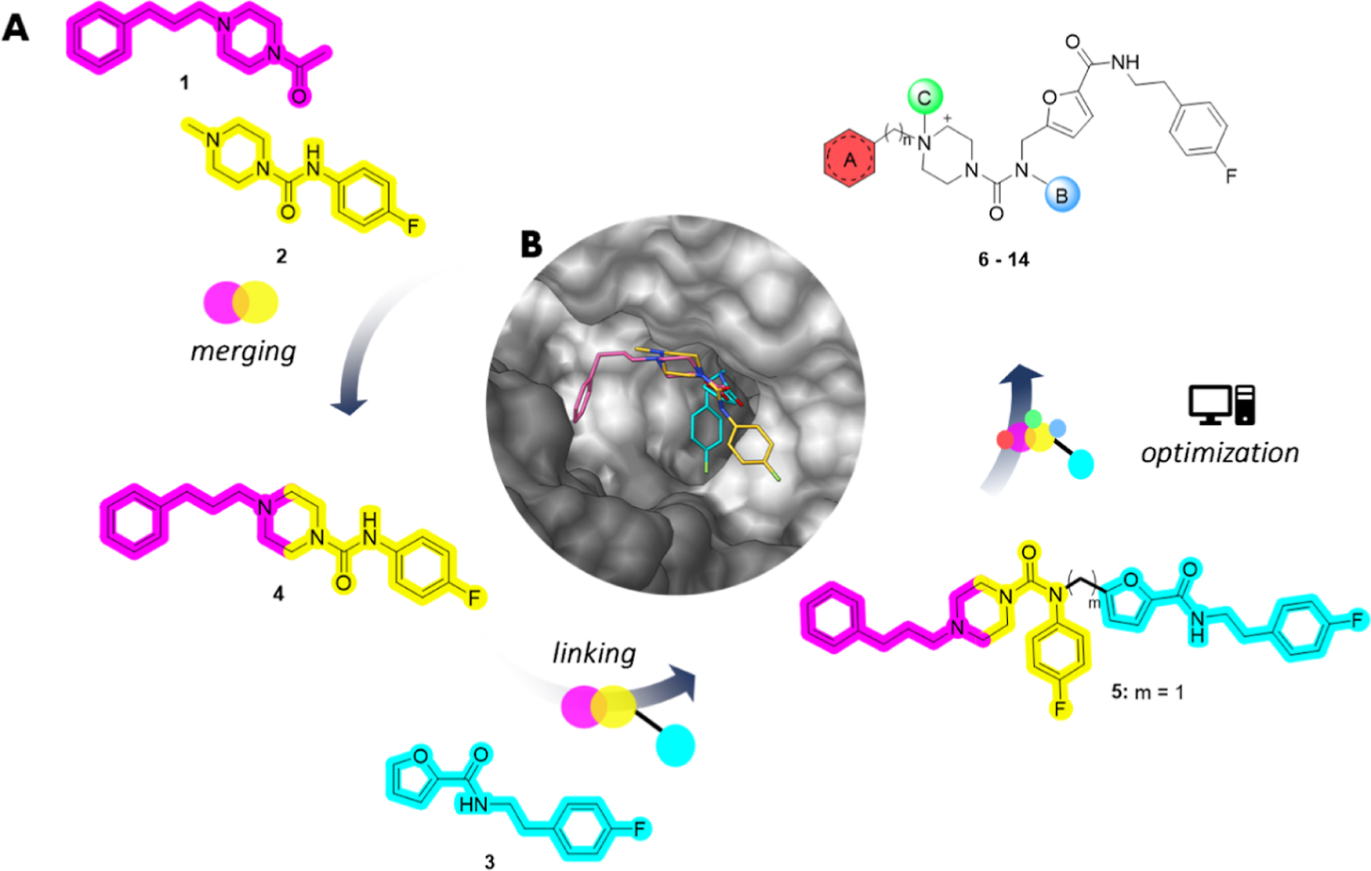
(A) Fragment-based drug design and structure-guided fragment elaboration of **4**–**14**. (B) **1**–**3** in complex with TR (PDB code 5S9W).^18^

Fragment-based drug discovery (FBDD) has been demonstrated as a powerful approach for enzymes difficult to inhibit^22^ (particularly beneficial for those targets that are large and open to solvent) as well as for identifying underexplored structural hot spots. Based on these considerations, we recently reported the first crystallographic fragment screening on TR,^18^ which led to the identification of new scaffolds with rapid follow-up possibilities.^23^ Taking three of them [109^18^ (**1**), 221^18^ (**2**), and 71^18^ (**3**), Figure 2A] into account, in this work, we explored a fragment elaboration cycle by harnessing fragment merging, linking, and growing strategies. Fragment-to-lead optimization (Figure 2B) was performed by combining structural data and *in silico* docking studies, leading to the design and synthesis of **4**–**14** (see Figure 3 and Table 1 for individual structures).

**Table 1:**
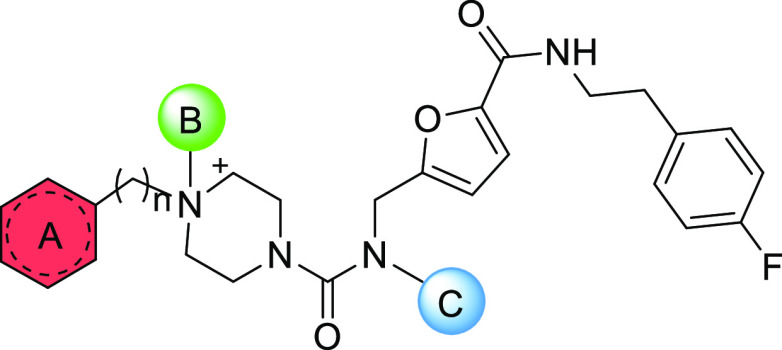

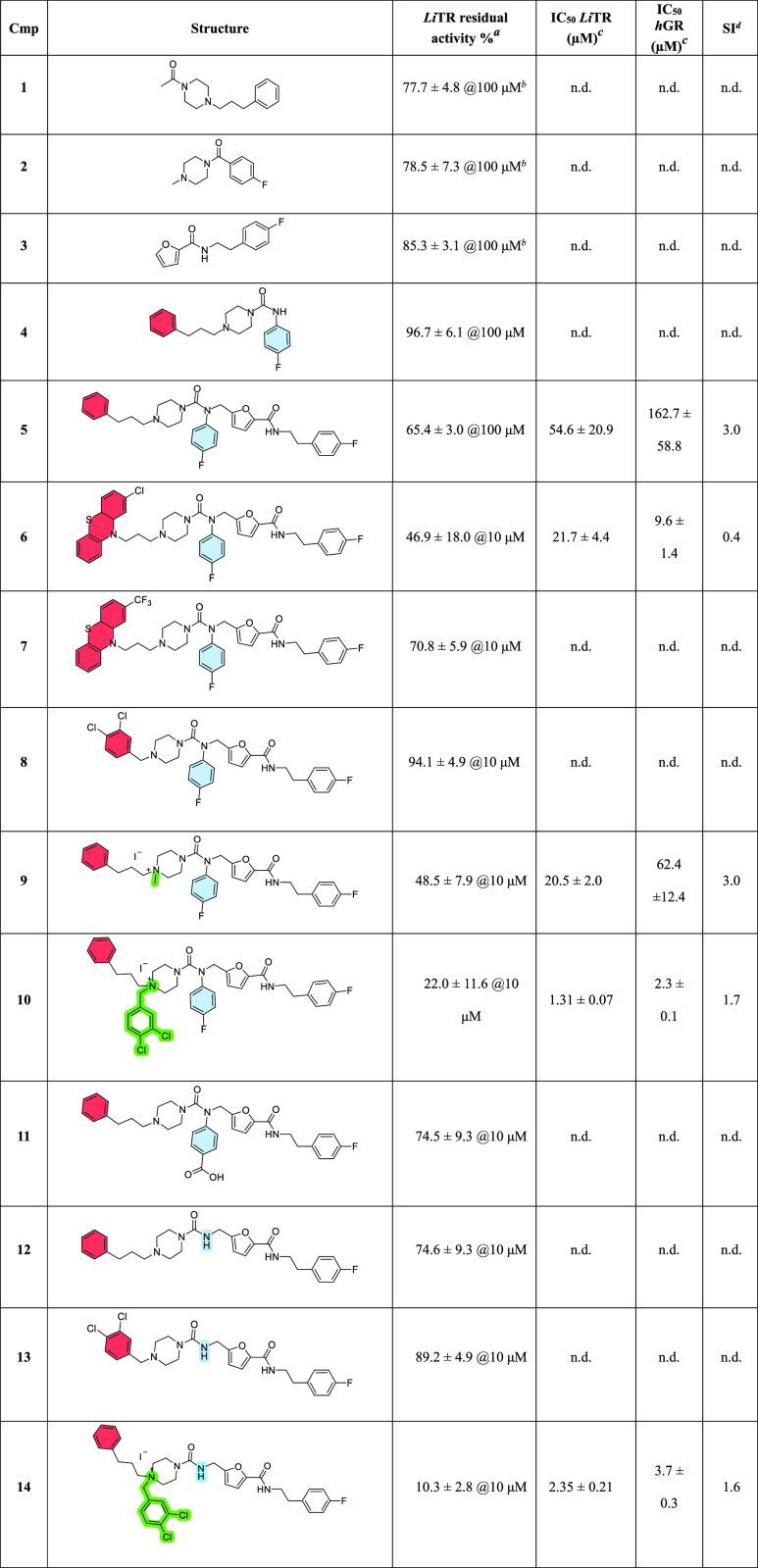
Inhibitory Activities of **4–14** for *Li*TR and *h*GR Enzymes and Their Selectivity Indexes^e^

a Residual enzyme activity (%) of the tested compounds at a given concentration (100 or 10 μM), compared to that of the positive control, are presented as the average from three independent measurements (mean ± SD, *n* = 3).

b Data taken from ref (18).

c IC_50_ values were determined upon fitting TR residual activity as a dose–response logistic equation defined as *y*min + (*y*max – *y*min)/(1 + (*x*/IC_50_)^slope).

d Selectivity index (SI): ratio of GR IC_50_/TR IC_50_; n.d. = not determined.

e *Li*TR inhibitory activities of **1–3** are reported for comparison.

For all of them, we assessed the *Leishmania infantum* TR (*Li*TR) inhibitory activity and the inhibition kinetics for the selected derivatives. Moreover, we determined the crystal structures and binding modes of three inhibitors (**9**, **10**, and **14**). Finally, we evaluated antileishmanial and cytotoxicity effects of **4**–**14** against a *L. infantum* reference strain through *in vitro* and *ex vivo* studies.

## Results and Discussion

### Design of **4** and **5** by Merging and Linking Strategies

The binding modes and activities of *fragments***1**–**3** at the Z-site have been described elsewhere.^18^ In line with the general behavior of fragments, their TR inhibitory profiles span from 22.3 to 14.7% at 100 μM concentration.^18^ In this work, such hit fragments were merged, linked, or grown into larger and potentially more potent compounds using a rational structure-based drug design approach.

As illustrated in Figure 3B, the crystal structures revealed an intriguing spatial shape complementarity between fragments **1** and **2** around the overlapped piperazine moiety. Thus, as a first step, we explored a fragment merging strategy combining the propylphenyl of **1** and the *p*-fluorophenyl of **2**, mounted on the two piperazine nitrogen atoms. This led to compound **4** (Figure 3A). Additionally, fragment **3** (Figure 3A) was found to target a narrow portion of the Z-site (Figure 3B), differing from *h*GR and in principle exploitable for the design of selective TR inhibitors.^18^ To this end, **4** provided us with an accessible, chemically derivatizable growth point at the secondary urea nitrogen atom, with the view of exploring the adjacent subpocket occupied by the furan of **3**. The linking strategy appears a suitable approach for contacting underexplored subpockets, especially for those targets endowed with a large binding site.^24^ Thus, we linked **3** to **4** by a methylene unit (*m* = 1), leading to **5** (Figure 3A). By reaching this second site, we assumed to potentially achieve improved potency and selectivity.

**Figure 4:**
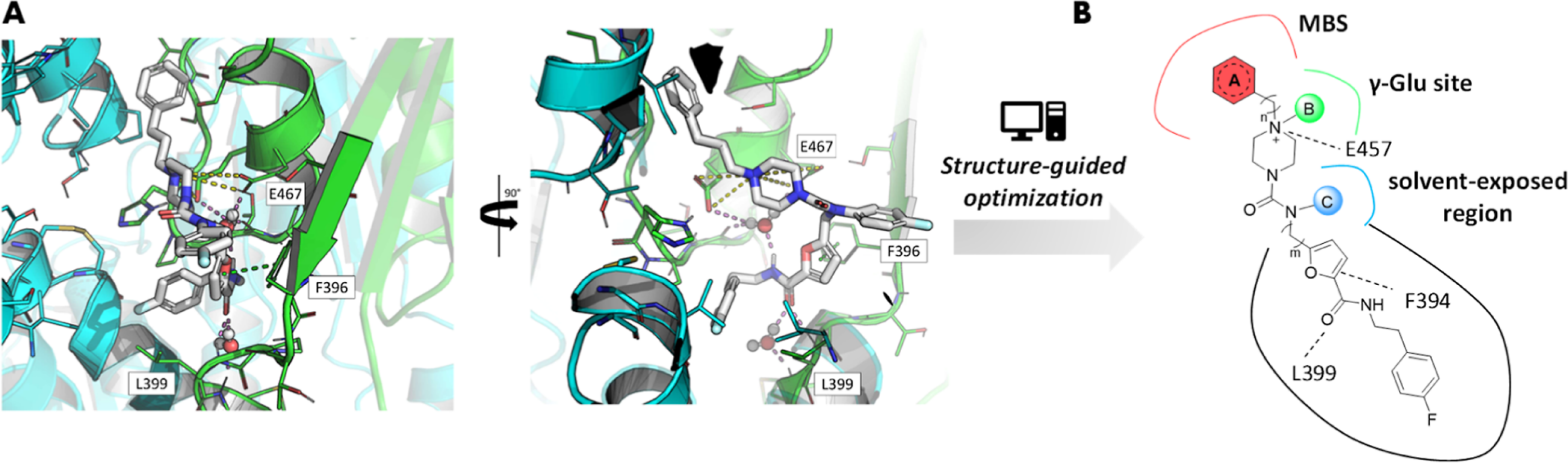
(A) Docking of **5** at the TR binding site (PDB code 5S9W).^18^ The ligand is represented as thick white sticks, water molecules are represented as spheres, and the main interacting residues are displayed as thin green and cyan sticks. (B) Schematic representation of the optimization strategy performed by analysis of adjacent hot spots (in red, green, and blue).

To test this hypothesis, docking studies of fragment-derived **5** in complex with *Li*TR were carried out with the GLIDE software, showing that it was able to preserve the experimental binding mode of the parent fragments (Figure S1).^18^ As illustrated in Figure 4A, **5** engages in several interactions at the Z-site, with the ethyl-*p-*fluorophenyl end of the 2-furoic amide portion pointing toward the lower and narrowed entrance of the interfacial cavity connecting the two trypanothione binding sites. Tracing **3**’s binding mode, the furan ring interacts with the side chain of F396, and the amide group engages in H-bonds with the protein backbone (L399) through water mediation. Noteworthily, the *N*-ureido *p*-fluorophenyl moiety protrudes to the aqueous bulk according to the crystal structure of **2**.^18^ The positively charged nitrogen of piperazine interacts alternatively with E466 or E467 by a charge-assisted H bond. Moreover, the propylphenyl aromatic terminus is accommodated into the upper side of the binding pocket directed toward the MBS and mainly engaged in van der Waals interactions.

Starting from the predicted binding mode of **5**, the design of fragment-derived compounds **6**–**14** (Figure 3A) was driven by knowledge-based approaches.

### Fragment Optimization: Design of **6–14**

The possibility of reaching adjacent hot spots around **5** guided further optimization into more potent TR inhibitors (Figure 4B). Particularly, we harnessed a strategy involving the combination of elements from known ligands to create *hybrid structures*.^25^ The fact that most of the structurally characterized TR inhibitors bind regions nearby that of **5** opened up the opportunity for combining **5** with these inhibitors and increasing binding valency. Thus, generation of fragment-derived compounds **6**–**10** was accomplished by performing structural modifications into region A and region B of **5** (Figure 4B), whereas compounds **11**–**14** were obtained by structural modification or simplification acting on region C (Figure 4B). Such modifications will be discussed separately as follows.

#### Targeting the MBS

The “three-point attachment”^17^ involving the MBS, Z-site, and bridged ionic interaction is known to increase the inhibitory activity. Accordingly, the predicted binding of **5** (Figure 4A) suggested that the MBS constitutes an additional site to be targeted for improving activity. For this reason, new compounds were designed as bearing in A (Figure 4B) an extended hydrophobic portion contacting the MBS. Thus, two classic MBS-binding motives, *i.e.*, chloro- and trifluoromethyl-phenothiazines (Cl-PTZ and CF_3_-PTZ from chlorpromazine and trifluoperazine), were introduced in place of the phenyl group of A, affording **6**–**7** (see Table 1 for structures), respectively. With the same aim, the 3,4-dichlorobenzyl (diClBn) moiety successfully exploited in **I**(17) (Figure 1A) was also explored, giving rise to hybrid structure **8** (Table 1).

#### Targeting the γ-Glu Site

Noticeably, most of the high-affinity TR inhibitors^17,26−29^ feature a basic tertiary or a quaternary nitrogen reported to establish interactions with the γ-Glu site (Figure 1C). Electrostatic analysis of the structures of TR and *h*GR also provides a rationale for the introduction of a permanent charge toward the design of the selective inhibitors.^30^ The effect of *N*-piperazine substitution on binding affinity was assessed by introducing a methyl or a diClBn group in B to afford quaternary ammonium salts **9** and **10**, respectively (Table 1).

#### Targeting the Solvent-Exposed Region

From both the crystal structure of **2** and the docking pose of **5**, the *p-*fluorophenyl moiety in position C (Figure 4B) seems to point toward the solvent. Solvent-exposed regions within the ligand-binding site provide opportunities for introducing charged and polar functional groups or water-solubility-enhancing groups as a means to improve the pharmacokinetic (PK) features and drug-like properties of a prospective drug candidate.^31^ Thus, the *p-*fluoro substituent of **5** was replaced with a more polar carboxylic acid (**11**). Noteworthily, a carboxylic acid can be also a structural handle for further proteolysis-targeting chimeras (PROTACs) or functionalized chemical probes.^31^

Additionally, with an eye to ligand efficiency, the *p-*fluorophenyl group in B of **5**, **8**, and **10** was removed while keeping in A the diClBn or the phenylpropane, leading to **12**, **13**, and **14** (Table 1), respectively.

For each cluster of modifications, docking studies were performed on representative compounds, which were able to confirm the above-described design (predicted binding modes are reported in Figure S2).

### Synthesis of Compounds **4–14**

**Scheme 1:**
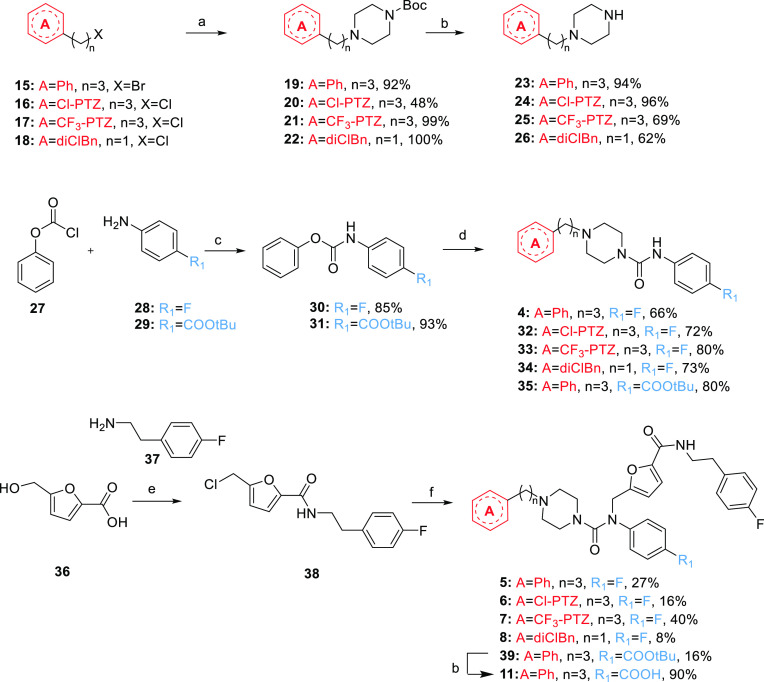
Synthesis of Compounds **4–8** and **11** Reagent and conditions: (a) *tert*-butyl piperazine-1-carboxylate, K_2_CO_3_, KI, CH_3_CN, 80 °C; (b) TFA, DCM, from 0 °C to r.t.; (c) Na_2_CO_3_, THF/EA/H_2_O, r.t.; (d) **23** or **26**, DCM or DMF, Et_3_N, 40 °C, overnight; (e) (i) SOCl_2_, toluene, 110 °C; (ii) toluene, microwave irradiation at 100 °C, 150 W, 10 min; (f) **4, 32–35**, NaH 60%, dry DMF, from 0 °C to r.t.

**Scheme 2:**
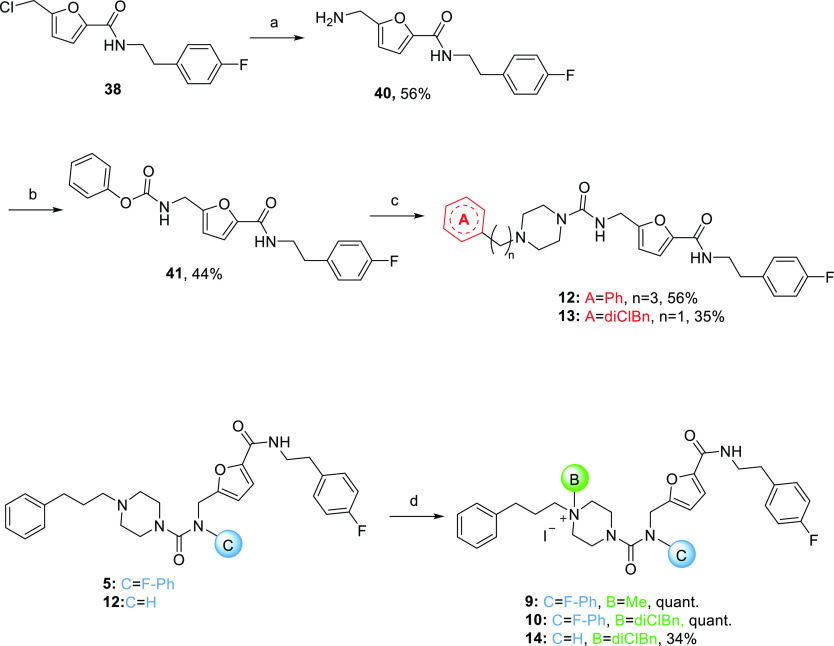
Synthesis of Compounds **9–10** and **12–14** Reagent and conditions: (a) (i) Phthalimide potassium salt, DMF, 60 °C; (ii) H_2_N–H_2_N, EtOH, reflux; (b) Na_2_CO_3_, THF/EA/H_2_O, r.t.; (c) **23** or **26**, DCM, Et_3_N, 40 °C, overnight; (d) MeI or 3,4-dichlorobenzyl chloride (diClBn), CH_3_CN, KI, 80 °C.

The preparation of initial fragments **1**–**3** was previously reported.^18^ Synthesis of compounds **4**–**8** and **11** is outlined in Scheme 1 whereas that of **9**–**10** and **12**–**14** in Scheme 2.

We started from commercially available or *in-house* prepared alkyl halides **15**–**18**, which reacted with *N*-Boc piperazine *via* a nucleophilic substitution to achieve **19**–**22** (Scheme 1). The *tert*-butyl group was then removed under acidic conditions, affording unsymmetrically substituted piperazine derivatives **23**–**26**.

Phenyl chloroformate (**27**) reacted with *p*-substituted anilines (**28**–**29**) to deliver the corresponding carbamates **30**–**31**. Those reacted with the appropriate piperazines **23**–**26** to afford the ureido-based merged fragment (**4**) and intermediates **32**–**35**.

Synthesis of fragment-derived **5**–**8** and **11** was started by 5-hydroxymethyl-2-furancarboxylic acid (**36**). Both hydroxy functions of **36** were substituted with chlorine in a single step by treatment with an excess of SOCl_2_. The acyl chloride functionality then reacted *in situ* with 2-(4-fluorophenyl)ethan-1-amine (**37**) under microwave irradiation to afford amide **38**. *N*-Alkylation of the ureido-based compounds **4** and **32**–**35** by alkyl halide **38** was obtained in the presence of sodium hydride to deliver fragment-derived compounds **5**–**8** and intermediate **39** (Scheme 1). The latter was subsequently deprotected in an acidic medium to provide **11**, featuring the free carboxylic acid handle.

To obtain the aliphatic ureido intermediates **12**–**13**, the alkyl halide functionality of **38** was transformed into primary amine (**40**) *via* Gabriel synthesis by treatment with potassium phthalimide followed by hydrazinolysis (Scheme 2). Phenyl chloroformate (**27**) reacted with **40** to form the activated carbamate intermediate **41**, which reacted with piperazines **23** or **26** affording aliphatic-ureido compounds **12** and **13**, respectively. Synthesis of piperazinium salts **9**, **10**, and **14** was performed in acetonitrile at 80 °C from piperazine derivatives **5** and **12** using the proper alkylating agent (methyl iodide or 3,4-dichlorobenzyl chloride) (Scheme 2).

### Evaluation of TR Enzymatic Inhibition and SAR

The inhibitory activity of the merged (**4**), linked (**5**), and fragment-derived **6**–**14** was assessed in an enzymatic assay using *Li*TR,^18^ and the results are reported in Table 1.

As expected for fragments–binders with low molecular weight and affinity −,^32^**1**–**3** were reported to have a low but evident effect on protein activity at 100 μM concentration (residual activity ranging from 77.7 to 85.3%).^18^

In order to make a direct comparison with **1**–**3**, the inhibition of merged compound **4** was initially tested at 100 μM concentration. Disappointingly, the expected increase in potency was not observed as **4** showed negligible inhibitory activity (96.7% residual activity). Conversely, when tested at the same concentration (100 μM), compound **5** caused a 35.1% decrease in TR activity (residual activity of 64.9%), indicating that the performed linking approach was successful and that fragment **3** might have a role in target recognition. This may also suggest that the one-methylene linker allowed a proper fit within the TR binding site.

The inhibitory activity was then assessed for larger fragment-derived **6**–**14** at 10 μM concentration, and IC_50_ values (Table 1) were calculated for **5** and for those compounds able to inhibit at least 50% of the enzyme activity (*i.e.*, **6**, **9**–**10**, and **14**, curves at Figure S3). For the most promising compounds **9**, **10**, and **14**, the inhibition constants (*K*_i_) were graphically determined from the Dixon plots (Figure S4).

Based on the data of Table 1, preliminary structure–activity relationship (SAR) can be captured by evaluating the effect of structural modifications on compound regions (A), (B), and (C) of Figure 4B.

#### Increasing Binding Valency by Targeting Both the Z-Site and MBS Resulted in Enhanced Inhibitory Activity

This strategy was pursued by modifying the phenyl ring of **5** (region A). The introduction of an extended hydrophobic system (as the Cl-PTZ) improved the inhibitory activity of **6** (IC_50_ of 21.7 μM) by more than 2-fold compared to that of the fragment-derived hit **5** (54.6 μM). CF_3_-PTZ-based compound **7** showed a residual activity of 70.8% at 10 μM, which was higher than that of **6** (residual activity 46.9%). This might suggest a critical role of the substituent in position 2 of the PTZ ring. After replacing the phenylpropyl moiety of **5** with a diClBn (**8**, 94.1% and **13**, 89.2%), the compounds resulted almost inactive, suggesting the importance of a proper spaced aromatic substituent to stabilize the interaction with the aromatic residues of the MBS.

#### *N*-Alkylation of Piperazine Increases Inhibitory Activity

The expected enhancement of the inhibitory activity was observed upon *N*-alkylation of piperazine on region B, as evident when comparing IC_50_ of **5** (IC_50_ = 54.6 μM) with that of its *N*-methyl derivative **9** (IC_50_ = 20.5 μM). Quaternization with the larger diClBn moiety (**10** and **14**) provided significant gains in IC_50_ values, 42-fold for **10** (IC_50_ = 1.31 μM) and 23-fold for **14** (IC_50_ = 2.35 μM) with respect to **5**. Noteworthily, piperazinium salts **9**, **10**, and **14** were the most active inhibitors of the series. The successful identification of **9**, **10**, and **14** prompted us to estimate the *K*_i_ value and mode of inhibition. The Dixon plots showed a linear competitive inhibition with the *K*_i_ values of 5.5 ± 0.2, 0.2 ± 0.1, and 0.8 ± 0.2 μM, respectively (Figure S4). Particularly, **10** and **14** are among the most active *Li*TR competitive inhibitors—in terms of *K*_i_ values—yet identified.^14,19^

#### Modifications Are Allowed on the Solvent-Exposed Region

Modifications at the solvent-exposed position (region C, Figure 4B) had little impact on enzyme activity. Replacement of fluorine on the *p*-phenyl position of **5** (residual activity = 65.4%) with a carboxylic acid as in **11** did not affect enzyme activity (74.5%). Similarly, the removal of the entire *p*-fluorophenyl group seemed to not influence the inhibitory activity (*e.g.*, **13***vs***8**).

**Figure 5:**
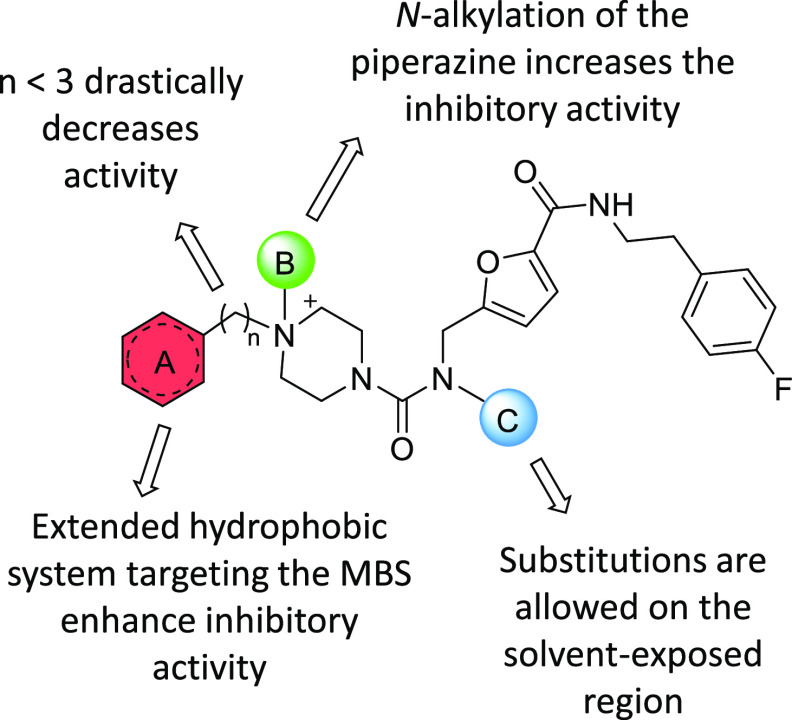
Summary of the SAR of the optimized fragment-derived compounds **6**–**14**.

Figure 5 summarizes the preliminary SAR around FBDD-derived hit **5**. As lipophilicity is an important requisite for targeting TR hydrophobic pockets,^33^ we also calculated logP and logD values of the compound series by using Swiss ADME and Chemaxon’s Playground, respectively (Table S1). However, no overt correlation between lipophilicity and TR inhibitory activity was evident.

Collectively, the performed modifications provided a significant enhancement of *Li*TR activity with three inhibitors (**9**, **10**, and **14**) showing *K*_i_ values in the low micromolar and submicromolar range. Remarkably, **10**, endowed with a completely new chemotype and a *K*_i_ value of 0.2 μM, stands among the most effective *Li*TR inhibitors so far developed.

In addition, to evaluate the selectivity of hit **5** and most promising inhibitors (**6**, **9**, **10**, and **14**), the inhibitory activity toward host *h*GR was assessed by the determination of IC_50_ values, followed by the calculation of selectivity indexes (SIs) (Table 1). Notwithstanding the careful considerations during compound design (*i.e.*, permanent charges and preserved network of interactions of the furan fragment known to target a selective TR subpocket), we observed an inhibitory effect also on *h*GR. Compound **6** displayed an even preferential inhibition of GR over TR (SI < 1), while **10** and **14** displayed poor selectivity (1 < SI < 3). Conversely, **5** and **9** showed a higher SI (SI = 3).

### X-ray Crystal Structures of *Tb*TR Bound to **9**, **10**, and **14** and Description of Their Binding Modes

**Figure 6:**
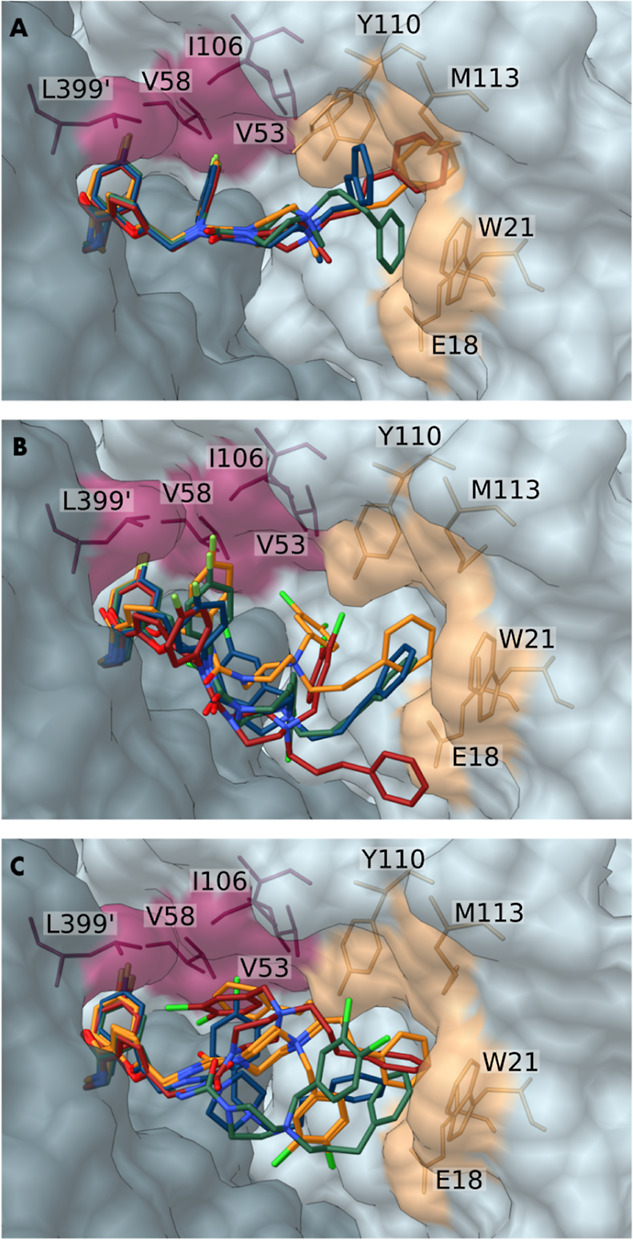
X-ray crystal structures of *Trypanosoma brucei* TR with **9**, **10**, and **14**. Compounds **9** (A), **10** (B), and **14** (C) are anchored to the Z-site (pink) and accommodate close to the MBS (orange). Monomers forming the *Tb*TR homodimer were differentiated for clarity and are shown as surfaces in shades of dark and light gray. **9**, **10**, and **14** and TR residues are displayed as sticks.

To validate the predicted binding mode for further rational structure-based drug design and ligand optimization, the structures of *Tb*TR with the most promising compounds **9**, **10**, and **14** were determined by X-ray crystallography (Figure 6). This was because the three compounds were designed starting from the fragment-screening experiment performed on *Tb*TR,^18^ which provided a more validated crystallization system delivering crystals with diffraction properties to considerably higher resolution than that of *Li*TR. A further advantage of using higher-resolution diffracting crystals was that they might enable a more accurate description of ligand orientations and interactions with the protein residues lining the MBS, γ-Glu site, and the Z-site, which, moreover, are conserved between the *Leishmania*/*Trypanosoma* species (Figure S5). Data reduction and refinement statistics are reported in Table S2. We identified 2 TR dimers in the asymmetric unit. A relevant electronic density was observed in each trypanothione binding pocket of both dimers (*i.e.*, four molecules in the asymmetric unit), which allowed us to confidently orient the compounds within the cavities (Figure S6).

The densities observed inside the trypanothione pocket for both TR dimers provided well-defined results for some portions of the compounds, namely, the ethyl-*p*-fluorophenyl and furan moieties, indicating that these common portions anchor **9**, **10**, and **14** (Figure 6A–C) to the Z-site by van der Waals and electrostatic interactions, as previously observed for fragment **3**. More precisely, the ethyl-*p*-fluorophenyl moiety explores the small cavity corresponding to the narrow entrance of the interfacial cavity at the TR dimer, connecting the two trypanothione binding sites. The resulting density overlaps with that of fragment **3** and is consistent with the reported docking studies.

**Figure 7:**
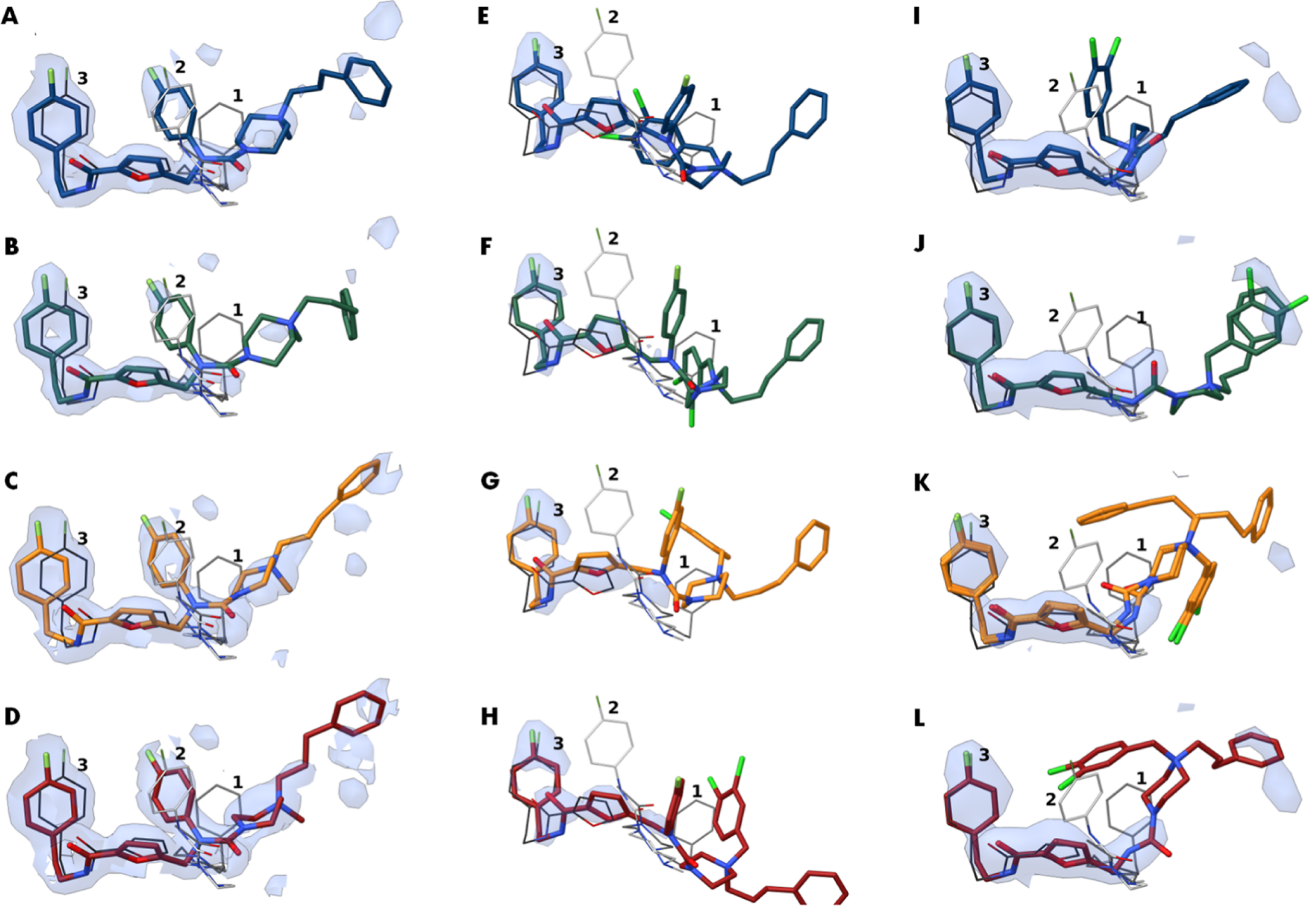
Conformations of **9**, **10**, and **14** within the trypanothione cavity of *Tb*TR. Two *Tb*TR homodimers are found in the asymmetric unit, resulting in 4 potential binding sites. The conformations adopted by **9** (A–D), **10** (E–H), or **14** (I–L) are shown in comparison with fragments **1**, **2**, and **3** reported by Fiorillo *et al.*(18) The 2Fo-Fc map is displayed as a blue volume contoured at 1σ.

The rest of the molecules of **9**, **10**, and **14** (including the piperazine ring, the *p*-fluorophenyl ring when present, the phenylpropyl and the dichlorobenzyl moieties) might adopt several conformations as illustrated in Figure 6. Regarding the TR/**9** complex (Figure 7A–D), the electronic density map of each trypanothione cavity is rather well-defined and allowed us to determine the positions of the entire molecule, except for the phenylpropyl, which seems to retain some mobility within the cavity.

The conformations reported in Figure 7A–L are the most probable ones, whose reconstructions are supported by the residual density present in the cavities. Their pronounced mobility is reflected by the elevated B factors (>100 Å^2^ for **10***vs* ∼65 Å^2^ for **14***vs* ∼49 Å^2^ for **9**, Figures S7–S9). Based on the refinement of the residual Fc–Fo difference map, we estimated that the occupancy for each compound ranged from 0.7 to 1.

Moreover, the binding of **9**, **10**, and **14** did not induce large TR conformational changes as shown by the respective 0.55 Å. 0.42 Å, and 0.57 Å overall root-mean-square deviation (rmsd) values obtained for the Cα, compared to that of apo-TR (2WOI PDB entry^20^). Nonethless, a discrete but notable shift of the Cα backbone of the 396−407 segment, which lines the Z-site, may be observed. Such a backbone stretch has already been described upon binding of **1**−**3** to the Z-site.^18^

As shown in Figure 7, the most potent inhibitors **9**, **10**, and **14** and parent fragments are partially overlapped. Specifically, the conformations of **9**, **10**, and **14** strikingly match that of fragment **3**, while they significantly differ from those of fragments **1** and **2**.

**Figure 8:**
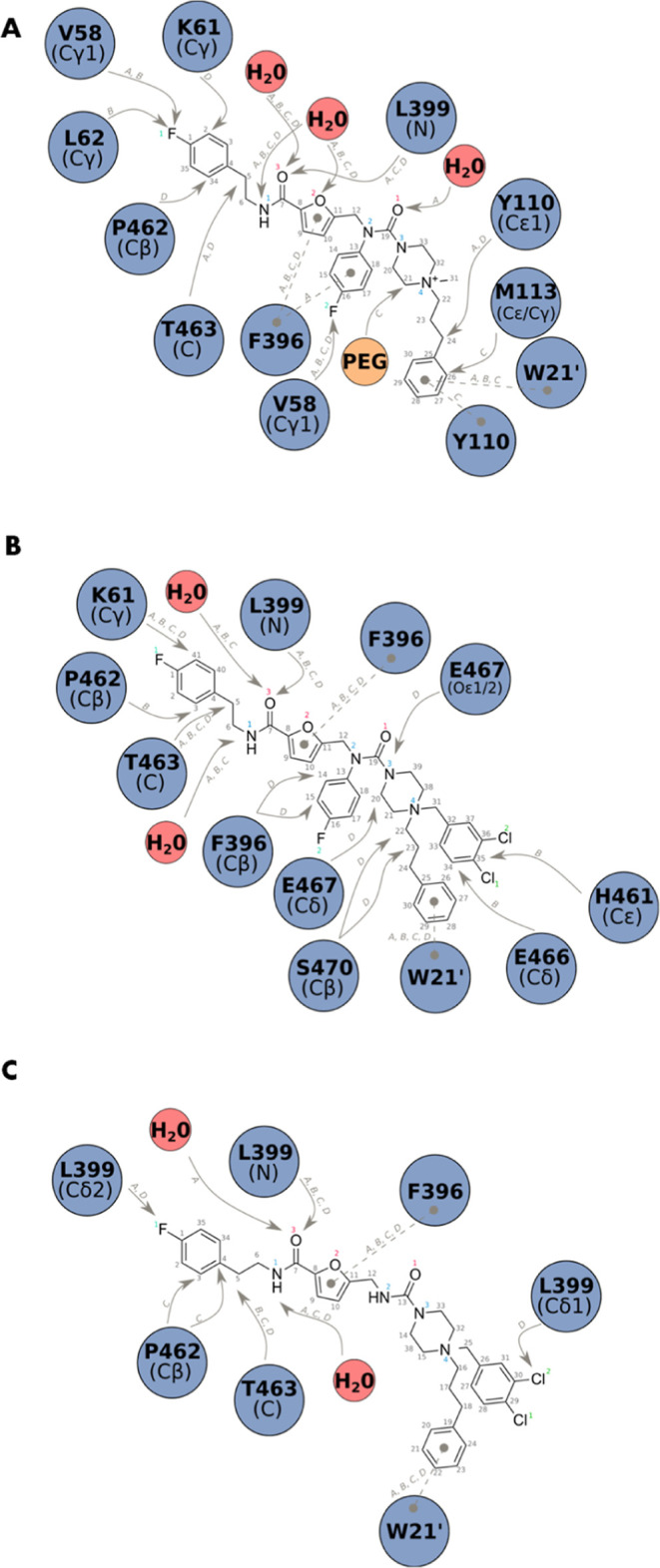
Interactions between TR and compounds **9** (A), **10** (B), and **14** (C). Only the interactions observed at less than 3.5 Å between the residues and compounds are reported. The A, B, C, and D gray letters refer to the compound numeration found in the PDB entries. Solid lines represent H bonds, electrostatic, and van der Waals interactions, while dashed lines represent stacking.

From the interaction network (Figure 8), it is clear that the furan and the ethyl-*p*-fluorophenyl groups anchor **9**, **10**, and **14** to the Z-site, consistently with the binding of the initial fragments. It is also evident that the dichlorobenzyl group—when present—protrudes toward the MBS together with the phenylpropyl moiety, making a T-shaped π-stacking interaction with W21. However, the electrostatic interaction between the piperazine ring of fragment-derived **9**, **10**, and **14** and E467 did not seem to be preserved as for **1** and **2**, differently from what was predicted by the docking studies.

Although the three compounds similarly accommodate in the trypanothione pocket, their conformations are somewhat different from what was expected based on the crystallographic structures of TR with **1**–**3**. Such discrepancies might arise from the fact that larger molecules derived from a cycle of elaboration are endowed with higher hindrance and less flexibility than those of the initial low-molecular-weight fragments.

Kinetic analysis and crystallographic data suggest that compounds **9**, **10**, and **14** compete with trypanothione as they bind to the Z-site thereby potentially impeding the substrate entrance into the cavity.

The crystal structure analysis may also allow us to speculate about the observed loss of selectivity (Table 1). Despite the presence of the bulkier side chains of M406 and Y106 in *h*GR (replacing the smaller L399 and A102 in *Tb*TR), it seems that the binding of **10** and **14** toward *h*GR is not fully hampered.

### Antileishmanial Activity and Cytotoxicity

To assess the antileishmanial activity, we evaluated the effect of **5**–**14** on *L. infantum* cellular growth, together with their cytotoxicity on mammalian macrophages. Amphotericin B (AmpB) was used as the reference drug, and the results are reported in Table 2. We performed an initial *in vitro* screening on *L. infantum* axenic amastigotes (efficacy expressed in EC_50_), a quick and easy phenotypic assay that uses the therapeutically relevant parasite stage (*i.e.*, the form infecting the human).^34^ In parallel, the cytotoxic concentrations (CC_50_ values in Table 2) were determined against human THP-1-derived macrophages, and the SIs were calculated as THP-1 macrophage CC_50_/axenic amastigote EC_50._ However, therapeutic efficacy in leishmaniasis involves parasite as well as host determinants, which require an integrated assessment of the host and parasite responses. To this end, for the selected compounds, we performed an *ex vivo* assay based on primary murine macrophages infected with natural amastigotes from spleens of infected hamsters. This allows us to preserve the host–parasite interaction and ensures a greater therapeutic relevance than that of the *in vitro* amastigote assay. Prior to this, we determined the effects on murine macrophage viability to exclude toxic concentrations to be employed in the *ex vivo* assay. Thus, the most active TR inhibitors **9**, **10**, and **14** were prioritized in the *ex vivo* intramacrophage amastigote assay (Table 2).

**Table 2:** Activity of the Tested Compounds in *In Vitro* Axenic Amastigote Assay and *Ex Vivo* Macrophages Infected by *L. infantum* Amastigotes and Cytotoxicity on Mammalian Macrophages

Cmp	axenic amastigote^a^EC_50_ ± SE (μM) (95% CI)	human THP-1-derived macrophage^a^CC_50_ ± SE (μM) (95% CI)	SI^b^	primary murine macrophage CC_50_ ± SE (μM) (95% CI)	intramacrophage amastigote EC_50_ ± SE (μM) (95% CI)
**5**	11.5 ± 2.1 (7.6–15.9)	9.6 ± 0.71 (8.2–11)	0.8	12.9 ± 0.9 (11.2–14.9)	nt^c^
**6**	12.0 ± 1.7 (8.9–16.0)	6.5 ± 0.6 (5.5–7.8)	0.5	9.1 ± 0.9 (7.5–11.1)	nt^c^
**7**	13.0 ± 1.5 (10.2–16.6)	23.4 ± 4.6 (15.6–34.9)	1.8	30.7 ± 6.1 (20.5–46.1)	nt^c^
**8**	10.3 ± 0.9 (8.5–12.5)	11.7 ± 1.6 (8.8–15.5)	1.1	14.4 ± 1.6 (11.6–17.9)	nt^c^
**9**	10.4 ± 1.1 (8.3–13.2)	38.4 ± 11 (21.6–68.3)	3.7	29.9 ± 4.2 (22.5–40.1)	15.3 ± 2.3 (11.3–20.7)
**10**	11.0 ± 1.9 (7.633–15.89)	16 ± 2.4 (12–22)	1.4	12.5 ± 0.8 (11.1–14.3)	nt^c^
**11**	25.6 ± 7.4 (14.26–45.93)	>50	>2	>50	nt^c^
**12**	14.1 ± 1.3 (11.7–16.9)	37.6 ± 13.8 (18–78.6)	2.7	26.7 ± 4.5 (18.9–37.8)	nt^c^
**13**	11.2 ± 0.7 (9.8–12.8)	21 ± 3.1 (15.4–28.3)	1.9	12.6 ± 0.9 (10.9–14.5)	nt^c^
**14**	8.98 ± 0.4 (8.2–9.9)	20.5 ± 3.6 (14.3–29.2)	2.3	12.7 ± 0.9 (11.1–14.7)	40% of reduction at 12.5 μM
**AmpB**	0.8 ± 0.4 (0.40–1.94)	21.1 ± 3	26.4	16.7 ± 2.05 (12.9–21.5)	0.54 ± 0.3 (0.18–1.64)

a 50% inhibitory concentration (EC_50_) ± standard error (SE); 50% cytotoxic concentration (CC_50_) ± SE.

b Selectivity index (SI) = human THP-1-derived macrophage CC_50_/axenic amastigote EC_50_.

c nt = not tested.

In the axenic amastigote model, all the tested compounds showed a fair, double-digit micromolar antileishmanial activity (apart from **14**), with EC_50_ values falling within a narrow range of concentrations (8.98 μM < EC_50_ < 25.6 μM). The compunds **5**–**14** were less potent than reference drug AmpB. No clear correlation between the enzymatic and cellular activity could be delineated for the series, though the lowest EC_50_ value was obtained for **14** (EC_50_ = 8.98 μM), which is at the same time the second-best TR inhibitor (*K*_iTR_ = 0.8 μM). On the other hand, **8**, which displayed marginal TR inhibition at 10 μM, showed a similar antileishmanial efficacy (EC_50_ = 10.3 μM). Thus, this might suggest the involvement of additional targets. The most active TR inhibitors bearing a quaternary ammonium (**9**, **10**, and **14**), which could have suffered for PK aspects, were indeed slightly more active than the rest of the compounds. Collectively, the EC_50_ values from the primary screening resulted in a flat SAR, and a direct correlation between the on-target inhibitory and phenotypic effect for all compounds is hard to trace. Moreover, when tested for their cytotoxicity on human macrophages, all compounds were slightly cytotoxic, though their CC_50_ values nearly approach that of AmpB. Only free carboxylic acid **11** did not show toxicity up to 50 μM (CC_50_ > 50 μM) and resulted in an SI > 2. Compounds **9**, **12**, and **14** were the less cytotoxic in the series (SI > 2). Interestingly, **6**, which was the compound with the lowest TR/GR SI, was concomitantly the one with the lowest human macrophage/axenic amastigote SI. Likely, the observed cytotoxic effects for the current series can be related to a GR off-target activity.

Then, a cytotoxicity assay was also performed on primary murine macrophages, the cells used for the *ex vivo* study. Noteworthily, no significant differences were observed between the primary murine and human THP-1-derived macrophages CC_50_ values.

Therefore, taking into account the cytotoxicity, the on-target activity, and the TR/GR SI, only **9** and **14** were prioritized for the *ex vivo* assay in intramacrophage amastigotes. Compound **10** exhibited a direct correlation between TR inhibition and phenotypic effects on axenic amastigotes; however, its cytotoxicity, which might arise from the GR inhibition (IC_50_ = 2.3 μM), prevented its *ex vivo* testing. Unfortunately, notwithstanding an SI of 2.3, we were unable to obtain an accurate intramacrophage EC_50_ value also for **14** due to its high cytotoxicity against the murine macrophages. On the contrary, thanks to a low toxicity, we were able to test **9** in the *ex vivo* assay. It was able to effectively inhibit the growth of the intramacrophage parasite form, showing an EC_50_ value (EC_50_ = 15.32 μM) that was consistent with that in the axenic amastigote model (EC_50_ = 10.42 μM).

## Conclusions

To bring out new opportunities for TR inhibition that could encompass new chemotypes and uncharacterized binding sites, we performed the first crystallographic fragment screening.^18^ Starting from that, we herein reported a fragment-to-lead optimization combining structural insights, *in silico* studies, and knowledge-based approaches.

The initial fragments **1**–**3** in their native binding showed positive features and provided an ideal starting point for medicinal chemistry elaboration. The recurrence of a piperazine moiety in fragments **1** and **2** and the potential of the furan-containing fragment **3** to target a small and specific subpocket in the Z-site provided an opportunity for developing **4** and **5** by the merging and linking strategies. Starting from hit **5**, SAR exploration and fragment growing were enabled by computational studies and available ligand-based information, leading to the design and synthesis of fragment-derived compounds **6**–**14**.

A trend of improvement in TR inhibitory activity was detected along the optimization process, from low percentages of inhibition at 100 μM for initial fragments **1**–**3** to IC_50_ values in the micromolar/submicromolar range for the best-performing compounds **9**, **10**, and **14**. Among all, **10** is the most potent fragment-derived TR inhibitor with a *K*_i_ value of 0.2 μM. To the best of our knowledge, it is among the strongest competitive inhibitors of *Li*TR enzyme yet identified.^14,19^ Unfortunately, compound **10** turned out to be also the most potent GR inhibitor (IC_50_ = 2.3 μM) of the set, which may account for its cytotoxicity on murine macrophages (CC_50_ = 12.5 μM). Nevertheless, a correlation between TR inhibition and phenotypic effects on axenic amastigotes (EC_50_ = 11.0 μM) might be observed for **10**. Thus, we uncovered a novel chemotype for TR inhibition, whose toxicity needs to be improved. On the other hand, compound **9** turned out to be a good TR inhibitor (*K*_i_ = 5.5 μM), together with a decent TR/GR selectivity (SI > 3). Furthermore, **9** was able to inhibit *L. infantum* growth in both *in vitro* (EC_50_ = 15.32 μM) and *ex vivo* models (EC_50_ = 10.42 μM). However, both **9** and **10** are quaternary ammonium inhibitors, which might not comply with the proposed target product profile of a treatment for visceral leishmaniasis, *i.e.*, “oral, safe, and well tolerated”.^35^ Indeed, PK properties of quaternary ammonium compounds are in many cases suboptimal; there might be a need for parenteral administration, and the neuromuscular blockage caused by many derivatives could raise safety concerns.^36^

To achieve better structural insights, the structures of *Tb*TR in complex with the most potent inhibitors **9, 10**, and **14** were solved. They also might help rationalize the observed lack of TR/GR selectivity. Quaternization of the piperazine promotes improvement in IC_50_ values compared to that of **5** (IC_50_ = 54.6 μM) by about 3-fold for **9** (IC_50_ = 20.5 μM), 42-fold for **10** (IC_50_ = 1.31 μM), and 23-fold for **14** (IC_50_ = 2.35 μM). However, this improvement does not seem to relate to the interaction between the positively charged ammonium and the protein but as shown in the solved structures, rather to the larger inhibitor structures, more efficiently occupying the binding pocket. With regard to selectivity, the binding of the optimized fragments **10** and **14** is probably allowed for *h*GR despite the presence of the bulkier side chains in *h*GR compared to those in TR. Finally, compound **9** appears to be more selective since it establishes weak interactions with MBS residues conserved in TR but not in GR.

Earlier, Ilari *et al*.^19^ demonstrated that 5-nitrothiophene carboxamides directed to the Z-site were endowed with high selectivity for TR over GR and that targeting the Z-site could be a good strategy for the development of selective compounds, However, the nitro group has been associated with genotoxicity and mutagenicity,^37^ and nitroarene is less desirable in drug design. Additionally, nitro group-bearing compounds might also act as TR redox cyclers, as nitroheterocyclic agents, *i.e*., nifurtimox and benznidazole.

Nonetheless, we assume that to drive the selectivity of TR inhibitors targeting the Z-site, bulkier charged groups could be introduced to anchor new *leads* to the L399/A102-lined cavity and/or the MBS.

In conclusion, although enhancing compound selectivity and activity fine-tuning are needed, we herein demonstrated the potentiality of FBDD applied to TR, a classic target often discarded for its challenges in discovering new (nonredox cycling) inhibitors. Thus, further medicinal chemistry optimization of **9** and **10** into lead-like compounds is warranted.

## Experimental Section

### Chemistry

All chemicals were purchased from Aldrich Chemistry (Milan, Italy), Alfa Aesar (Milan, Italy), and FluoroChem (Cambridge, UK) and were of the highest purity. The solvents were of analytical grade. Reaction progress was followed by thin-layer chromatography on precoated silica gel 60 F254 plates (Merck, Darmstadt, Germany). Chromatographic separations were performed on 0.040– 0.063 mm silica gel 40 columns *via* the flash method (Merck). The ^1^H nuclear magnetic resonance (^1^H NMR) and ^13^C NMR spectra were recorded on a Varian Gemini spectrometer (Varian Medical System Italia, Milan, Italy) at 400 and 100 MHz, respectively, in CDCl_3_ solutions unless otherwise indicated. Chemical shifts (δ) were reported as parts per million relative to tetramethylsilane, used as the internal standard; coupling constants (*J*) are reported in hertz (Hz). Standard abbreviations indicating spin multiplicities are given as follows: s (singlet), d (doublet), dd (double doublet), ddd (doublet of doublets of doublets), t (triplet), q (quartet), and m (multiplet). Ultra- high-performance liquid chromatography (HPLC)–mass spectrometry analyses were run on a Waters ACQUITY Arc system (Milan, Italy) consisting of a QDa mass spectrometer equipped with an electrospray ionization (ESI) interface and a 2489 UV/vis detector. The detected wavelengths were 254 and 365 nm. Analyses were performed on an XBridge BEH C18 column with a 10 × 2.1 mm internal diameter (particle size 2.5 μm) with an XBridge BEH C18 VanGuard Cartridge precolumn with a 5 × 2.1 mm internal diameter (particle size 1.8 μm) (Waters). The mobile phases were H_2_O (0.1% formic acid) and MeCN (0.1% formic acid). ESI in positive and negative mode was applied in the mass scan range of 50–1200 Da. The authors used a generic method and linear gradient: 0–0.78 min, 20% B; 0.78–2.87 min, 20–95% B; 2.87–3.54 min, 95% B; 3.54–3.65 min, 95–20% B; 3.65–5.73, 20% B. The flow rate was 0.8 mL/min. High-resolution mass spectra were recorded on a Waters Xevo G2-XS quadrupole time-of-flight apparatus operating in an electrospray mode. Compounds were named based on the naming algorithm developed by CambridgeSoft and used in ChemBioDraw Ultra (PerkinElmer, Milan, Italy, version 20.0). All the tested compounds were found to have >95% purity. Synthesis of compounds **1**, **2**, and **3** has been reported by Fiorillo *et al.*(18)

### General Procedure A

In a 50 mL sealed vessel, a mixture of *tert-*butyl piperazine-1-carboxylate (1.5–2.2 equiv) and proper alkyl halide **15**–**18** (1.0 equiv) in acetonitrile was stirred in the presence of K_2_CO_3_ (1.5 equiv) and KI (0.01 equiv). The mixture was heated to 80 °C for 3–18 h. Upon completion, the hot suspension was filtered, the residue was washed with acetone several times, the collected filtrates were concentrated *in vacuo*, and the resultant crude was purified by silica gel column chromatography; (ii) the reaction mixture was diluted in ethyl acetate and washed with HCl 1 N aqueous solution.

### General Procedure B

The *N*-Boc-protected compounds were dissolved in dichloromethane (0.2 M). Trifluoroacetic acid (20.0 equiv) was added at 0 °C. The ice bath was removed, and the resulting mixture was left under stirring at r.t. for 2 h. Upon reaction completion, the mixture was diluted with additional dichloromethane (10 mL) and washed with saturated NaHCO_3_ aqueous solution (2 × 15 mL). The organic phase was dried over anhydrous Na_2_SO_4_, filtered, and concentrated *in vacuo*.

### General Procedure C

To a solution of carbamate **30**–**31** (1.0 equiv) in dichloromethane (0.2 M) or dimethylformamide were added TEA (2.0 equiv) and the corresponding amine **23**–**26** (1.0 equiv). The reaction mixture was heated at 40 °C overnight. The solvent was removed *in vacuo*, and the resulting crude was purified by silica gel chromatography.

### General Procedure D

SOCl_2_ (10.0 equiv) was added dropwise to a suspension of the appropriate carboxylic acid (1.0 equiv) in toluene (0.2 M). The reaction was refluxed at 110 °C for 2 h before heating was stopped. Evaporation of the volatiles *in vacuo* gave the desired compound (assumed 100% yield), which was employed in the next synthetic step without further purification. To a microwave vial charged with a magnetic stirring bar and the proper acyl chloride (2.10 equiv) in toluene, **37** (1.0 equiv) was added dropwise. The reaction was carried out under microwave irradiation at 100 °C, 150 W for 10 min. The solvent was removed *in vacuo*, and the crude was purified by silica gel chromatography.

### General Procedure E

To a stirred solution of the ureido-based compounds **4, 32–35** (1.0 equiv) in dry dimethylformamide or dichloromethane under an inert atmosphere, sodium hydride 60% dispersion in mineral oil (2.5 equiv) was added at 0 °C. After 30 min, a solution of **38** (1.0 equiv) in dry dimethylformamide (2.50 mL) was added dropwise. The reaction mixture was stirred for 18 h at room temperature. The sodium hydride was quenched with water, and the residue was reconstituted with ethyl acetate (15 mL) and water (15 mL). The organic layer was washed with water (3 × 15 mL), dried over anhydrous Na_2_SO_4_, filtered, and concentrated *in vacuo*. The resulting residue was purified by silica gel column chromatography.

### General Procedure F

To compound **5** or **12** (1.0 equiv) in acetonitrile (0.2 M), KI (0.01 equiv) and the proper alkylating agent were added (4.0 equiv) in a pressure tube, and the reaction was refluxed overnight. Upon completion, the volatiles were removed *in vacuo*, and the compound was purified by trituration from diethyl ether.

### *N-*(4-Fluorophenyl)-4-(3-phenylpropyl)piperazine-1-carboxamide (**4**)

Following Procedure C, the desired compound was obtained from carbamate **30** (520 mg, 2.55 mmol) and amine **23** (590 mg, 2.55 mmol) in dichloromethane (13 mL). Purification by silica gel column chromatography (dichloromethane/methanol, 9.5:0.5) gave **4** as a white solid (574 mg, 66%). ^1^H NMR (400 MHz, DMSO-*d*_6_) δ 8.51 (s, 1H), 7.45–7.37 (m, 2H), 7.31–7.13 (m, 5H), 7.09–7.04 (t, *J* = 8.8 Hz, 2H), 3.45–3.39 (m, 4H), 2.60 (t, *J* = 7.6 Hz, 4H), 2.39–2.33 (m, 2H), 2.33–2.26 (m, 2H), 1.79–1.68 (m, 2H). ^13^C NMR (100 MHz, DMSO-*d*_6_) δ 157.7 (d, *J*_*C–F*_ = 240.0 Hz), 155.4, 142.4, 137.3 (4C), 128.7 (d, *J*_*Cm–F*_ = 8.0 Hz, 2C), 126.1, 121.7 (d, *J*_*Cp-F*_ = 7.6 Hz), 115.2 (d, *J*_*Co–F*_ = 22.0 Hz, 2C), 57.6 (2C), 53.1 (2C), 44.1, 33.3, 28.5. LCMS (ESI): Calcd for C_20_H_25_FN_3_O [M + H]^+^*m*/*z*: 342.20; found, 342.42.

### *N*-((5-((4-Fluorophenethyl)carbamoyl)furan-2-yl)methyl)-*N*-(4-fluorophenyl)-4-(3-phenylpropyl)piperazine-1-carboxamide (**5**)

Following general procedure E, **5** was obtained from ureido compound **4** (170 mg, 0.49 mmol) and alkyl halide **38** (140 mg, 0.49 mmol) in dry dimethylformamide (2.50 mL). Purification by silica gel column chromatography (dichloromethane/methanol/ammonia 32% aqueous solution, 9.8:0.2:0.02) yielded **5** as a yellow oil (80 mg, 27%). ^1^H NMR (400 MHz, CDCl_3_) δ 7.27–7.19 (m, 2H), 7.20–7.10 (m, 5H), 7.02–6.94 (m, 6H), 6.25 (d, *J* = 3.4 Hz, 2H), 4.72 (s, 2H), 3.61–3.54 (m, 2H), 3.25–3.16 (m, 4H), 2.85 (t, *J* = 7.2 Hz, 2H), 2.58 (t, *J* = 7.6 Hz, 2H), 2.31–2.22 (m, 2H), 2.22–2.14 (m, 4H), 1.77–1.65 (m, 2H). ^13^C NMR (100 MHz, CDCl_3_) δ 161.61 (d, *J*_*C–F*_ = 244.5 Hz), 160.0, 159.9 (d, *J*_*C–F*_ = 246.1 Hz), 158.2, 153.9, 146.9, 141.8, 141.1 (d, *J*_*Cp-F*_ = 3.2 Hz), 134.4 (d, *J*_*Cp-F*_ = 3.2 Hz), 130.1(d, *J*_*Cm–F*_ = 7.8 Hz, 2C), 128.3 (2C), 128.2 (2C), 126.3 (d, *J*_*Cm–F*_ = 8.2 Hz, 2C), 125.7, 116.3 (d, *J*_*Co–F*_ = 22.6 Hz, 2C), 115.3 (d, *J*_*Co–F*_ = 21.2 Hz, 2C), 114.9, 110.7, 57.6 (2C), 52.5 (2C), 48.5, 45.6, 40.3, 35.0, 33.4, 28.3. LCMS (ESI): Calcd for C_34_H_37_F_2_N_4_O_3_ [M + H]^+^*m/z*: 587.28; found, 587.40.

### 4-(3-(2-Chloro-10*H*-phenothiazin-10-yl)propyl)-*N*-((5-((4-fluorophenethyl)carbamoyl)furan-2-yl)methyl)-*N*-(4-fluorophenyl)piperazine-1-carboxamide (**6**)

Following general procedure E, **6** was obtained from ureido compound **32** (0.12 g, 0.25 mmol) and alkyl halide **38** (70 mg, 0.25 mmol) in dry dimethylformamide (3 mL). Purification by silica gel column chromatography (petroleum ether/ethyl acetate/methanol/ammonia 32% aqueous solution, 6:4:0.5:0.05) yielded **6** as a yellow oil (33 mg, 16%). ^1^H NMR (400 MHz, CDCl_3_) δ 7.20–7.12 (m, 2H), 7.09 (dd, *J* = 7.5, 6.4 Hz, 2H), 7.02–6.87 (m, 9H), 6.87–6.81 (m, 2H), 6.80 (d, *J* = 1.8 Hz, 1H), 6.24 (d, *J* = 3.4 Hz, 1H), 6.19 (t, *J* = 5.9 Hz, 1H), 4.70 (s, 2H), 3.86 (t, *J* = 6.6 Hz, 2H), 3.59 (dd, *J* = 13.5, 6.9 Hz, 2H), 3.14 (s, 4H), 2.84 (t, *J* = 7.1 Hz, 2H), 2.37 (t, *J* = 6.7 Hz, 2H), 2.16 (s, 4H), 1.85 (dd, *J* = 13.3, 6.7 Hz, 2H). ^13^C NMR (100 MHz, CDCl_3_) δ 161.6 (d, *J*_*C–F*_ = 244.5 Hz), 160.4, 159.9, 159.8 (d, *J*_*C–F*_ = 246.1 Hz), 158.3, 153.9, 146.9, 146.3, 144.4, 141.0 (d, *J*_*Cp-F*_ = 3.1 Hz), 134.4 (d, *J*_*Cp-F*_ = 3.1 Hz), 133.1, 130.1 (d, *J*_*Cm–F*_ = 7.8 Hz, 2C), 126.3 (d, *J*_*Cm–F*_ = 8.2 Hz, 2C), 124.7 (2C), 116.3 (d, *J*_*Co–F*_ = 22.6 Hz, 2C), 115.7, 115.7, 115.3 (d, *J*_*Co–F*_ = 21.1 Hz, 2C), 114.9, 110.7, 55.0 (2C), 52.6 (2C), 48.5, 45.6 44.9, 40.3, 35.0, 23.9. LCMS (ESI): Calcd for C_40_H_39_ClF_2_N_5_O_3_S [M + H]^+^*m/z*: 742.24; found, 742.20.

### *N-*((5-((4-Fluorophenethyl)carbamoyl)furan-2-yl)methyl)-*N*-(4-fluorophenyl)-4-(3-(2-(trifluoromethyl)-10*H*-phenothiazin-10-yl)propyl)piperazine-1-carboxamide (**7**)

Following general procedure E, **7** was obtained from ureido compound **33** (0.20 g, 0.38 mmol) and alkyl halide **38** (0.13 g, 0.45 mmol) in dry dimethylformamide (5 mL). Purification by silica gel column chromatography (petroleum ether/ethyl acetate/methanol/ammonia 32% aqueous solution, 6:4:0.5:0.05) yielded **7** as a yellow oil (120 mg, 40%) ^1^H NMR (400 MHz, CDCl_3_): δ = 7.18–7.09 (m, 6H), 7.01–6.91 (m, 9H), 6.86 (d, *J* = 8 Hz, 1H), 6.24 (d, *J* = 4 Hz, 1H), 6.13 (t, *J* = 6 Hz, 1H), 4.70 (s, 2H), 3.91 (t, *J* = 6 Hz, 2H), 3.58 (q, *J* = 7 Hz, 2H), 3.09 (tbr, *J* = 6 Hz, 4H), 2.83 (t, *J* = 8 Hz, 2H), 2.36 (t, *J* = 8 Hz, 2H), 2.14 (t, *J* = 4 Hz, 4H), 1.82 (p, *J* = 7 Hz, 2H). ^13^CNMR (100 MHz, CDCl_3_) δ 161.1 (d, *J*_*C–F*_ = 244.5 Hz), 160.3, 159.9(d, *J*_*C–F*_ = 246.1 Hz), 158.2, 153.9, 146.9, 145.6, 144.1, 140.8 (d, *J*_*Cp-F*_ = 3.1 Hz), 134.4 (d, *J*_*Cp-F*_ = 3.1 Hz), 130.2, 130.1 (d, *J*_*Cm–F*_ = 7.8 Hz, 2C), 129.9, 129.6, 129.3, 127.5, 127.5, 127.4, 126.3 (d, *J*_*Cm–F*_ = 8.2 Hz, 2C), 124.0 (2C), 116.4 (d, *J*_*Co–F*_ = 22.6 Hz, 2C), 115.8, 115.4, 115.2(d, *J*_*Co–F*_ = 21.1 Hz, 2C), 114.9, 111.9, 110.7, 54.9 (2C), 52.5 (2C), 48.5, 45.5, 44.9, 34.9, 23.8. LCMS (ESI): Calcd for C_41_H_39_F_5_N_5_O_3_S [M + H]^+^*m/z*: 776.27; found, 776.27.

### 4-(3,4-Dichlorobenzyl)-*N*-(4-fluorophenyl)-*N*-((5-(phenethylcarbamoyl)furan-2-yl)methyl)piperazine-1-carboxamide (**8**)

Following general procedure E, **8** was obtained from ureido compound **34** (110 mg, 0.30 mmol) and alkyl halide **38** (80 mg, 0.30 mmol) in dry dimethylformamide (2.5 mL). Purification by silica gel column chromatography (dichloromethane/methanol, 9.5:0.5) yielded **8** as a yellow oil (15 mg, 8%). ^1^H NMR (400 MHz, CD_3_OD) δ 7.70 (s, 1H), 7.62 (d, *J* = 8.2 Hz, 1H), 7.40 (d, *J* = 7.7 Hz, 1H), 7.21 (ddd, *J* = 18.1, 8.7, 5.1 Hz, 5H), 7.10 (t, *J* = 8.6 Hz, 2H), 7.03–6.95 (m, 2H), 6.94 (d, *J* = 3.4 Hz, 1H), 6.30 (d, *J* = 3.4 Hz, 1H), 4.23 (br s, 2H), 3.88 (br s, 2H), 3.52 (t, *J* = 7.3 Hz, 2H), 3.02 (br s, 8H), 2.85 (t, *J* = 7.3 Hz, 2H). ^13^C NMR (100 MHz, CDCl_3_) δ 161.0, (d, *J*_*C–F*_ = 244.5 Hz), 160.2, 159.8 (d, *J*_*C–F*_ = 246.1 Hz), 158.1, 153.6, 146.8, 140.7 (d, *J*_*Cp-F*_ = 3.2 Hz), 134.2 (d, *J*_*Cp-F*_ = 3.2 Hz), 130.1 (d, *J*_*Cm–F*_ = 7.8 Hz, 2C), 128.5 (2C) 128.0 (2C), 126.2 (d, *J*_*Cm–F*_ = 8.2 Hz, 2C), 126.1, 116.3, 116.1 (d, *J*_*Co–F*_ = 22.6 Hz, 2C), 115.3 (d, *J*_*Co–F*_ = 21.2 Hz, 2C), 114.7, 110.5, 67.9, 61.2, 52.1, 50.6, 48.3, 45.3, 40.2, 34.8. LCMS (ESI): Calcd for C_32_H_32_Cl_2_FN4O_3_ [M + H]^+^*m/z*: 610.53; found, 610.47.

### 4-(((5-((4-Fluorophenethyl)carbamoyl)furan-2-yl)methyl)(4-fluorophenyl)carbamoyl)-1-methyl-1-(3-phenylpropyl)piperazin-1-ium Iodide (**9**)

Following general procedure F, the desired compound was obtained from **5** (50 mg, 0.08 mmol) and MeI (48 mg, 0.34 mmol) in acetonitrile (1 mL). Purification by trituration from diethyl ether gave **9** as a yellow solid. Yield 100% ^1^H NMR (400 MHz, CDCl_3_) δ 7.30 (t, *J* = 7.2 Hz, 2H), 7.22–7.13 (m, 5H), 7.11–7.05 (m, 4H), 6.99 (t, *J* = 8.7 Hz, 3H), 6.92 (d, *J* = 3.4 Hz, 1H), 6.30 (t, *J* = 4.6 Hz, 1H), 6.23 (d, *J* = 3.4 Hz, 1H), 4.75 (s, 2H), 3.75–3.68 (m, 2H), 3.59 (m, 5H), 3.53–3.39 (m, 4H), 3.34 (s, 3H), 2.88 (t, *J* = 7.1 Hz, 2H), 2.76 (t, *J* = 7.1 Hz, 2H), 2.12–1.96 (m, 2H). ^13^C NMR (100 MHz, CDCl_3_) δ 162.7, 161.6, 160.3, 159.2, 158.1, 152.9, 147.2, 139.2, 138.9, 134.5, 130.2, 130.1, 128.8 (2C), 128.3 (2C), 126.8, 126.7, 117.2, 117.0, 115.4, 115.2, 114.6, 110.7, 63.6, 59.5, 53.3, 48.7, 47.2, 40.4, 39.8, 34.9, 31.8, 30.8, 23.7, 22.9. LCMS (ESI): Calcd for C_35_H_39_F_2_N_4_O_3_ [M-I]^+^*m/z*: 601.10; found, 601.06.

### 1-(3,4-Dichlorobenzyl)-4-(((5-((4-fluorophenethyl)carbamoyl)furan-2-yl)methyl)(4-fluorophenyl)carbamoyl)-1-(3-phenylpropyl)piperazin-1-ium Iodide (**10**)

Following general procedure F, the desired compound was obtained from **5** (32 mg, 0.05 mmol) and 1,2-dichloro-4-(chloromethyl)benzene (39 mg, 0.2 mmol) in acetonitrile (1 mL). Purification by trituration from diethyl ether gave **10** as an orange solid. Yield 100%. ^1^H NMR (400 MHz, CDCl_3_) δ 7.56 (d, *J* = 1.8 Hz, 1H), 7.36–7.28 (m, 2H), 7.26 (t, *J* = 3.5 Hz, 1H), 7.22 (d, *J* = 7.0 Hz, 1H), 7.14 (ddd, *J* = 16.8, 8.7, 5.0 Hz, 5H), 7.08–7.01 (m, 3H), 7.01–6.93 (m, 3H), 6.87 (d, *J* = 3.5 Hz, 1H), 6.35 (t, *J* = 6.0 Hz, 1H), 6.21 (d, *J* = 3.4 Hz, 1H), 4.76 (s, 2H), 4.70 (s, 2H), 3.85 (d, *J* = 15.1 Hz, 3H), 3.58 (dd, *J* = 13.6, 6.8 Hz, 5H), 3.31 (dd, *J* = 26.8, 12.4 Hz, 7H), 2.85 (t, *J* = 7.2 Hz, 3H), 2.78 (t, *J* = 6.5 Hz, 2H), 2.27–2.13 (m, 2H). ^13^C NMR (100 MHz, CDCl_3_) δ 165.3, 164.8, 162.7, 160.3, 159.3, 159.2, 158.2, 153.0, 147.0, 138.8, 136.2, 134.3, 134.0, 131.6, 130.2, 130.1, 129.1 (2C), 128.7 (2C), 127.1, 125.2, 117.3, 117.0, 115.4, 115.2, 114.5, 110.6, 57.5, 55.3, 53.3, 49.0, 40.5, 39.8, 34.9, 32.0, 30.8, 23.9, 22.7. LCMS (ESI): Calcd for C_41_H_42_Cl_2_F_2_N_4_O_3_ [M-I + H]^+^*m/z*: 747.26; found, 747.27.

### 4-(*N*-((5-((4-Fluorophenethyl)carbamoyl)furan-2-yl)methyl)-4-(3-phenylpropyl)piperazine-1-carboxamido)benzoic Acid (**11**)

The desired compound is obtained from **36** (290 mg, 0.43 mmol) and TFA (0.66 mL, 8.6 mmol) in dichloromethane (5 mL). **11** was obtained as a white solid (280 mg, 90%). ^1^H NMR (400 MHz, CDCl_3_) δ 7.93 (d, *J* = 8.2 Hz, 2H), 7.24 (t, *J* = 7.2 Hz, 2H), 7.20–7.01 (m, 7H), 6.98–6.85 (m, 3H), 6.34–6.20 (m, 2H), 4.81 (d, *J* = 11.6 Hz, 2H), 3.79 (s, 2H), 3.54 (dd, *J* = 13.4, 6.8 Hz, 2H), 3.45 (s, 2H), 3.21 (s, 2H), 2.94 (t, *J* = 16.0 Hz, 3H), 2.80 (t, *J* = 6.9 Hz, 2), 2.63 (t, *J* = 7.0 Hz, 4H), 2.01 (d, *J* = 13.8 Hz, 2H). ^13^C NMR (100 MHz, CDCl_3_) δ 168.00 162.8, 160.3, 158.7, 158.3, 153.2, 148.0, 146.9, 139.1, 134.2, 131.7, 130.2, 130.1, 128.7 (2C), 128.1(2C), 127.2, 126.6, 123.1, 115.5, 115.3, 114.8, 110.6, 56.8, 51.2, 48.0, 42.8, 40.5, 34.8, 32.4, 28.1, 24.8, 22.45. LCMS (ESI): Calcd for C_35_H_38_FN_4_O_5_ [M + H]^+^*m/z*: 613.28; found, 613.20.

### *N*-((5-((4-Fluorophenethyl)carbamoyl)furan-2-yl)methyl)-4-(3-phenylpropyl)piperazine-1-carboxamide (**12**)

Following general procedure C, the desired compound was obtained from carbamate **41** (50 mg, 0.14 mmol) and amine **23** (20 mg, 0.14 mmol) in dimethylformamide (1.5 mL). Purification by silica gel column chromatography (dichloromethane/methanol, 9.5:0.5) gave **12** as a white solid (32 mg, 56%). ^1^H NMR (400 MHz, CDCl_3_) δ 7.30–7.24 (m, 3H), 7.16 (t, *J* = 6.9 Hz, 4H), 7.03–6.94 (m, 2H), 6.58 (s, 1H), 6.27 (s, 1H), 4.90 (t, *J* = 5.1 Hz, 1H), 4.39 (s, 2H), 3.64–3.57 (m, 2H), 3.38 (d, *J* = 5.0 Hz, 4H), 2.86 (d, *J* = 7.2 Hz, 2H), 2.62 (t, *J* = 7.6 Hz, 2H), 2.41 (d, *J* = 5.0 Hz, 4H), 2.39–2.33 (m, 2H), 1.81 (dd, *J* = 15.2, 7.7 Hz, 2H). ^13^C NMR (100 MHz, CD_3_OD) δ 206.0, 162.9, 160.5, 158.5, 157.2, 154.9, 147.3, 142.0, 134.5, 130.3, 130.2, 128.4, 128.4, 125.9, 115.6, 115.4, 115.0, 109.7, 57.9, 52.8, 43.9, 40.6, 37.9, 35.1, 33.6, 29.8, 28.5. LCMS (ESI): Calcd for C_28_H_34_FN_4_O_3_ [M + H]^+^*m/z*: 493.60; found, 493.90.

### 4-(3,4-Dichlorobenzyl)-*N*-((5-((4-fluorophenethyl)carbamoyl)furan-2-yl)methyl)piperazine-1-carboxamide (**13**)

Following general procedure C, the desired compound was obtained from carbamate **41** (0.15 mmol, 60 mg) and amine **26** (0.12 mmol, 30 mg) in dimethylformamide (1.5 mL). Purification by silica gel column chromatography (dichloromethane/methanol/toluene, 9.6:0.4:0.1) gave **13** as a white solid (23 mg, 35%). ^1^H NMR (400 MHz, CDCl_3_) δ 7.43 (d, *J* = 1.5 Hz, 1H), 7.38 (d, *J* = 8.2 Hz, 1H), 7.17 (dt, *J* = 8.6, 3.6 Hz, 3H), 7.04–6.94 (m, 3H), 6.38 (s, 1H), 6.30 (d, *J* = 3.3 Hz, 1H), 4.82 (d, *J* = 5.1 Hz, 1H), 4.41 (d, *J* = 5.5 Hz, 2H), 3.62 (dd, *J* = 13.5, 6.8 Hz, 2H), 3.46 (s, 2H), 3.43–3.34 (m, 4H), 2.91–2.83 (m,2H), 2.47–2.37 (m, 4H). ^13^C NMR (100 MHz, CDCl_3_) δ 162.9, 158.4, 157.1, 154.8, 150.6, 147.3, 130.5, 130.4, 130.3, 130.2, 128.3, 115.6, 115.4, 115.0, 109.7, 61.7, 52.7, 43.9, 40.9, 38.0, 35.2, 29.8, 20.6. LCMS (ESI): Calcd for C_26_H_28_Cl_2_FN_4_O_3_ [M + H]^+^*m/z*: 534.43; found, 534.32.

### 1-(3,4-Dichlorobenzyl)-4-(((5-((4-fluorophenethyl)carbamoyl)furan-2-yl)methyl)carbamoyl)-1-(3-phenylpropyl)piperazin-1-ium Iodide (**14**)

Following general procedure F, the desired compound was obtained from **12** (45 mg, 0.09 mmol) and 1,2-dichloro-4-(chloromethyl)benzene (72 mg, 0.37 mmol) in acetonitrile (5 mL). Purification by trituration from diethyl ether gave **14** as a yellow solid (21 mg, 35%). ^1^H NMR (400 MHz, CD_3_OD) δ 7.68 (d, *J* = 2.1 Hz, 1H), 7.51 (d, *J* = 8.2 Hz, 2H), 7.35–7.20 (m, 7H), 7.17 (d, *J* = 8.3 Hz, 1H), 6.98 (dd, *J* = 11.1, 6.4 Hz, 2H), 4.61 (s, 2H), 4.36 (s, 2H), 3.99 (d, *J* = 16.0 Hz, 2H), 3.66–3.40 (m, 9H), 3.38 (s, 2H), 2.89–2.82 (m, 2H), 2.73 (t, *J* = 7.0 Hz, 2H), 2.23 (s, 2H), 1.60 (s, 1H), 0.87 (d, *J* = 7.0 Hz, 2H). ^13^C NMR (100 MHz, CD_3_OD) δ 159.4, 157.6, 155.7, 146.7, 139.6, 135.0, 134.5, 133.0, 132.1, 131.2, 130.9, 130.2, 130.1, 128.5, 128.4, 126.9, 126.4, 114.7, 114.6, 114.5, 108.8, 65.5, 62.6, 57.2, 55.1, 40.5, 37.4, 37.1, 34.4, 31.5, 29.4, 23.1. LCMS (ESI): Calcd for C_35_H_38_Cl_2_FN_4_O_3_ [M-I]^+^*m/z*: 651.23 (for the ^35^Cl isotope), 653.23 (for the ^37^Cl isotope); found, 651.41 (for the ^35^Cl isotope), 653.89 (for the ^37^Cl isotope).

### *tert*-Butyl 4-(3-Phenylpropyl)piperazine-1-carboxylate (**19**)

Following general procedure A, the desired compound was obtained from *tert*-butyl piperazine-1-carboxylate (890 mg, 4.78 mmol) and alkyl halide **15** (630 mg, 3.18 mmol) in acetonitrile (1 mL). Purification by silica gel column chromatography (dichloromethane/methanol/ammonia 32% aqueous solution, 9.8:0.2:0.02) yielded **19** as a colorless oil (554 mg, 92%). ^1^H NMR (400 MHz, CDCl_3_) δ 7.31–7.23 (m, 2H), 7.22–7.14 (m, 3H), 3.44 (t, *J* = 5.2 Hz, 4H), 2.64 (t, *J* = 7.6 Hz, 2H), 2.40–2.35(m, 6H), 1.84 (q, *J* = 7.6 Hz, 2H), 1.45 (s, 9H). LCMS (ESI): Calcd for C_18_H_29_N_2_O_2_ [M + H]^+^*m/z*: 305.22; found, 305.05.

### *tert-*Butyl 4-(3-(2-Chloro-10*H*-phenothiazin-10-yl)propyl)piperazine-1-carboxylate (**20**)

Following general procedure A, the desired compound was obtained from *tert*-butyl piperazine-1-carboxylate (40 mg, 2.14 mmol) and alkyl halide **16** (300 mg, 0.97 mmol) in acetonitrile (6 mL) in the presence of K_2_CO_3_ and KI for 18 h. Purification by silica gel column chromatography (dichloromethane/methanol/ammonia 32% aqueous solution, 9.8:0.2:0.02) yielded **20** as a colorless oil (220 mg, 48%). ^1^H NMR (400 MHz, CDCl_3_) δ 7.13 (ddd, *J* = 9.3, 8.3, 1.4 Hz, 2H), 7.01 (d, *J* = 8.1 Hz, 1H), 6.96–6.91 (m, 1H), 6.87 (ddd, *J* = 13.4, 6.7, 2.9 Hz, 3H), 3.92 (t, *J* = 6.8 Hz, 2H), 3.42–3.31 (m, 4H), 2.46 (t, *J* = 6.9 Hz, 2H), 2.40–2.28 (m, 4H), 1.93 (p, *J* = 6.9 Hz, 2H), 1.45 (s, 9H). LCMS (ESI): Calcd for C_24_H_31_ClN_3_O_2_S [M + H]^+^*m/z*: 461.04; found, 461.03.

### *tert*-Butyl 4-(3-(2-(Trifluoromethyl)-10*H*-phenothiazin-10-yl)propyl)piperazine-1-carboxylate (**21**)

Following general procedure A, the desired compound was obtained from t*ert*-butyl piperazine-1-carboxylate (330 mg, 1.78 mmol) and alkyl halide **17** (410 mg, 1.18 mmol) in acetonitrile (6 mL) in the presence of K_2_CO_3_ and KI for 18 h. Purification by silica gel column chromatography (petroleum ether/ethyl acetate/methanol/ammonia 32% aqueous solution, 7:3:0.1:0.01) yielded **21** as a colorless oil (580 mg, 99%). ^1^H NMR (400 MHz, CDCl_3_) δ = 7.20–7.10 (m, 4H), 7.04 (s, 1H), 6.93 (q, *J* = 8 Hz, 2H), 3.98 (t, *J* = 4 Hz, 2H), 3.35 (t, *J* = 4 Hz, 4H), 2.47 (t, *J* = 8 Hz, 2H), 2.33 (t, *J* = 4 Hz, 4H), 1.93 (m, 2H), 1.44 (s, 9H). LCMS (ESI): Calcd for C_25_H_30_F_3_N_3_O_2_S [M]^+^*m/z*: 493.21; found, 439.20.

### *tert*-Butyl 4-(3,4-Dichlorobenzyl)piperazine-1-carboxylate (**22**)

Following general procedure A, the desired compound was obtained from *tert*-butyl piperazine-1-carboxylate (200 mg, 1.07 mmol) and **18** (139 mg, 0.71 mmol) in acetonitrile (2.5 mL) in the presence of K_2_CO_3_ and KI for 18 h. The reaction mixture was diluted in ethyl acetate and washed with HCl 1 N aqueous solution (3 × 10 mL) to afford **22** as a colorless oil (100%). ^1^H NMR (400 MHz, CDCl_3_) δ 7.74–7.64 (m, 2H), 7.54 (d, *J* = 8.2 Hz, 1H), 4.09 (s, 2H), 1.56 (s, 8H), 1.43 (s, 9H). LCMS (ESI): Calcd for C_16_H_23_Cl_2_N_2_O_2_ [M + H]^+^*m/z*: 346.27; found, 346.87.

### 1-(3-Phenylpropyl)piperazine (**23**)

Following general procedure B, *N*-Boc amine **19** (1.03 g, 3.38 mmol) was treated with trifluoroacetic acid (7.70 g, 67.6 mmol) in dichloromethane (17 mL) at 0 °C to afford **23** as a colorless oil (233 mg, 94%). ^1^H NMR (400 MHz, CDCl_3_) δ 7.28–7.20 (m, 2H), 7.19–7.10 (m, 3H), 2.87 (t, *J* = 4.8 Hz, 4H), 2.65–2.56 (m, 2H), 2.46–2.27 (m, 6H), 2.20 (br, 1H), 1.85–1.73 (m, 2H). LCMS (ESI): Calcd for C_13_H_21_N_2_ [M + H]^+^*m/z*: 205.17; found, 205.30.

### 2-Chloro-10-(3-(piperazin-1-yl)propyl)-10*H*-phenothiazine (**24**)

Following general procedure B, *N*-Boc amine **20** (200 mg, 0.43 mmol) was treated with trifluoroacetic acid (0.66 mL 8.60 mmol) in dichloromethane (5 mL) at 0 °C to afford **24** as a colorless oil (150 mg, 96%).^1^H NMR (400 MHz, CDCl_3_) δ 7.17–7.08 (m, 2H), 7.00 (d, *J* = 8.0 Hz, 1H), 6.95–6.89 (m, 2H), 6.88 (d, *J* = 2.0 Hz, 1H), 6.86 (dd, *J* = 4.8, 2.0 Hz, 1H), 3.90 (t, *J* = 6.9 Hz, 2H), 2.85 (t, *J* = 4.9 Hz, 4H), 2.44 (t, *J* = 7.0 Hz, 2H), 2.39 (s br, 4H), 1.93 (dd, *J* = 11.8, 4.7 Hz, 2H). LCMS (ESI): Calcd for C_19_H_23_ClN_3_S [M + H]^+^*m/z*: 360.13; found, 360.92.

### 10-(3-(Piperazin-1-yl)propyl)-2-(trifluoromethyl)-10*H*-phenothiazine (**25**)

Following general procedure B, *N*-Boc amine **21** (309 mg, 0.60 mmol) was treated with trifluoroacetic acid (0.93 mL, 12.16 mmol), in dichloromethane (6 mL) at 0 °C, to afford **25** as a colorless oil (170 mg, 69%). ^1^H NMR (400 MHz, CDCl_3_): δ = 7.20–7.10 (m, 4H), 7.04 (s, 1H), 6.94 (m, 2H), 3.96 (t, *J* = 8 Hz, 2H), 2.84 (t, *J* = 6 Hz, 4H), 2.46 (t, *J* = 6 Hz, 2H), 2.38 (br s, 4H), 1.93 (p, *J* = 8 Hz, 2H), 1.69 (s, 1H). LCMS (ESI): Calcd for C_20_H_23_F_3_N_3_S [M + H]^+^*m/z*: 394.16; found, 394.15.

### 1-(3,4-Dichlorobenzyl)piperazine (**26**)

Following general procedure B, **22** (260 mg, 0.76 mmol) was treated with trifluoroacetic acid (1.2 mL, 15.18 mmol), in dichloromethane (4 mL) at 0 °C, to afford **26** as a colorless oil (116 mg, 62%). ^1^H NMR (400 MHz, CDCl_3_) δ 7.43 (d, *J* = 1.7 Hz, 1H), 7.37 (d, *J* = 8.2 Hz, 1H), 7.15 (dd, *J* = 8.2, 1.8 Hz, 1H), 3.43 (d, *J* = 6.3 Hz, 2H), 2.95–2.85 (m, 4H), 2.41 (s, 4H). LCMS (ESI): Calcd for C_11_H_15_Cl_2_N_2_ [M + H]^+^*m/z*: 246.16; found, 246.98.

### Phenyl (4-Fluorophenyl)carbamate (**30**)

Phenyl chloroformate (**27**, 0.99 mmol, 1.1 equiv) was added dropwise to a solution of aniline **28** (0.09 mL, 0.90 mmol, 1.0 equiv) and Na_2_CO_3_ (57 mg, 0.54 mmol, 0.6 equiv) in a mixture of ethyl acetate, tetrahydrofuran, and H_2_O (1.36, 0.27, and 0.27 mL, respectively) and cooled at 0 °C. The reaction mixture was stirred at room temperature overnight, then it was concentrated *in vacuo* to remove organic solvents. Water was added to the residue, and the resulting precipitate was recovered by filtration in vacuo, washed with water, and dried to give compound **30** as a gray solid. Yield 85%. ^1^H NMR (400 MHz, CDCl_3_) δ 7.44–7.37 (m, 4H), 7.26–7.22 (m, 1H), 7.21–7.17 (m, 2H), 7.08–7.01 (m, 2H), 6.88 (br s, 1H). LCMS (ESI): Calcd for C_13_H_11_FNO_2_ [M + H]^+^*m/z*: 232.08; found, 232.11.

### *tert*-Butyl 4-((Phenoxycarbonyl)amino)benzoate (**31**)

Phenyl chloroformate (**27**, 3.96 mmol, 0.50 mL, 1.1 equiv) was added dropwise to a solution of aniline **29** (700 mg, 3.60 mmol, 1.0 equiv) and Na_2_CO_3_ (2.16 mmol, 0.6 equiv) in a mixture of ethyl acetate, tetrahydrofuran, and H_2_O (5, 1, and 1 mL, respectively) cooled at 0 °C. The reaction mixture was stirred at room temperature overnight, then it was concentrated *in vacuo* to remove the ,organic solvents. Water was added to the residue, and the resulting precipitate was recovered by filtration *in vacuo*, washed with water, and dried to give compound **31** as a gray solid. Yield 93%. ^1^H NMR (400 MHz, CDCl_3_) δ 7.97 (d, *J* = 8.7 Hz, 2H), 7.50 (d, *J* = 8.7 Hz, 2H), 7.46–7.36 (m, 2H), 7.30–7.22 (m, 1H), 7.22–7.17 (m, 2H), 1.59 (s, 9H). LCMS (ESI): Calcd for C_14_H_12_NO_4_ [M-*tert*Bu + H]^+^*m*/*z*: 258.08; found, 258.09.

### 4-(3-(2-Chloro-10*H*-phenothiazin-10-yl)propyl)-*N*-(4-fluorophenyl)piperazine-1-carboxamide (**32**)

Following general procedure C, the desired compound was obtained from carbamate **30** (140 mg, 0.40 mmol) and amine **24** (90 mg, 0.40 mmol) in dichloromethane (4 mL). Purification by silica gel column chromatography (dichloromethane/methanol, 9.5:0.5) gave **32** as a white solid (140 mg, 72%).^1^H NMR (400 MHz, DMSO-*d*_6_) δ 7.43–7.37 (m, 2H), 7.22–7.15 (m, 2H), 7.14–7.08 (m, 2H), 7.05 (d, *J* = 2.2 Hz, 1H), 7.04–6.98 (m, 2H), 6.97–6.92 (m, 2H), 3.92 (t, *J* = 6.6 Hz, 2H), 3.36–3.30 (m, 4H), 2.49–2.41 (m, 4H), 2.38 (t, *J* = 6.6 Hz, 2H), 1.82–1.72 (m, 2H). LCMS (ESI): Calcd for C_26_H_27_ClFN_4_OS [M + H]^+^*m/z*: 498.04; found, 498.20.

### *N*-(4-Fluorophenyl)-4-(3-(2-(trifluoromethyl)-10*H*-phenothiazin-10-yl)propyl)piperazine-1-carboxamide (**33**)

Following general procedure C, the desired compound was obtained from carbamate **30** (128 mg, 0.48 mmol) and amine **25** (190 mg, 0.48 mmol) in dichloromethane (5 mL). Purification by silica gel column chromatography (dichloromethane/methanol, 9.5:0.5) gave **33** as a white solid (200 mg, 80%).^1^H NMR (400 MHz, CDCl_3_): δ = 7.30–7.26 (m, 2H), 7.21–7.12 (m, 4H), 7.05 (s, 1H), 6.95 (m, 4H), 6.24 (s, 1H), 4.00 (t, *J* = 8 Hz, 2H), 3.40 (t, *J* = 8 Hz, 4H), 2.51 (t, *J* = 8 Hz, 2H), 2.44 (t, *J* = 8 Hz, 4H), 1.95 (p, *J* = 8 Hz, 2H). LCMS (ESI): Calcd for C_27_H_27_F_4_N_4_OS [M + H]^+^*m/z*: 531.18; found, 531.18.

### 4-(3,4-Dichlorobenzyl)-*N*-(4-fluorophenyl)piperazine-1-carboxamide (**34**)

Following general procedure C, the desired compound was obtained from carbamate **30** (150 mg, 0.64 mmol) and amine **26** (156 mg, 0.64 mmol) in dimethylformamide (7 mL). Purification by silica gel column chromatography (dichloromethane/methanol, 9.8:0.2) gave **34** as a greyish solid (150 mg, 73%). ^1^H NMR (400 MHz, CDCl_3_) δ 7.43 (d, *J* = 1.7 Hz, 1H), 7.38 (d, *J* = 8.2 Hz, 1H), 7.33–7.23 (m, 2H), 7.15 (dd, *J* = 8.2, 1.8 Hz, 1H), 6.96 (dd, *J* = 11.9, 5.5 Hz, 2H), 6.30 (s, 1H), 3.48 (d, *J* = 5.1 Hz, 6H), 2.46 (d, *J* = 5.0 Hz, 4H). LCMS (ESI): Calcd for C_18_H_19_Cl_2_FN_3_O [M + H]^+^*m/z*: 383.27; found, 383.45.

### *tert*-Butyl 4-(4-(3-Phenylpropyl)piperazine-1-carboxamido)benzoate (**35**)

Following general procedure C, the desired compound was obtained from carbamate **31** (340 mg, 1.66 mmol) and amine **23** (520 mg, 1.66 mmol) in dichloromethane (9 mL). Purification by silica gel column chromatography (dichloromethane/methanol/ammonia 32% aqueous solution 9.8:0.2:0.02) gave **35** as a brownish oil (570 mg, 80%). ^1^H NMR (400 MHz, CDCl_3_) δ 7.95–7.86 (m, 2H), 7.44–7.37 (m, 2H), 7.28 (dd, *J* = 11.9, 4.7 Hz, 2H), 7.21–7.15 (m, 3H), 3.55–3.47 (m, 4H), 2.70–2.60 (m, 2H), 2.50–2.43 (m, 4H), 2.42–2.34 (m, 2H), 1.83 (dt, *J* = 15.1, 7.6 Hz, 2H), 1.58 (s, 9H). LCMS (ESI): Calcd for C_25_H_34_N_3_O_3_ [M + H]^+^*m/z*: 424.26; found, 424.40.

### 5-(Chloromethyl)-*N*-(4-fluorophenethyl)furan-2-carboxamide (**38**)

Following general procedure D, **38** was obtained from **36** (500 mg, 3.51 mmol) and SOCl_2_ (2.50 mL, 35.10 mmol) and then reacted with 2-(4-fluorophenyl)ethan-1-amine (**37**, 0.20 mL, 1.67 mmol). Purification by silica gel column chromatography (petroleum ether/ethyl acetate, 6:4) yielded **38** as a pale brownish solid (286 mg, 67%). ^1^H NMR (400 MHz, CDCl_3_) δ 7.19 (dd, *J* = 8.3, 5.4 Hz, 2H), 7.05 (d, *J* = 3.4 Hz, 1H), 7.01 (t, *J* = 8.7 Hz, 2H), 6.49–6.43 (m, 1H), 6.40 (s, 1H), 4.55 (s, 2H), 3.65 (dd, *J* = 13.5, 6.9 Hz, 2H), 2.90 (t, *J* = 7.2 Hz, 2H). LCMS (ESI): Calcd for C_14_H_14_ClFNO_2_ [M + H]^+^*m/z*: 282.07; found, 282.09.

### *tert*-Butyl 4-(*N*-((5-((4-Fluorophenethyl)carbamoyl)furan-2-yl)methyl)-4-(3-phenylpropyl)piperazine-1-carboxamido)benzoate (**39**)

Following general procedure E, **39** was obtained from ureido compound **35** (550 mg; 1.29 mmol) and alkyl halide **38** (362 mg, 1.29 mmol) in dry dimethylformamide (5 mL). Purification by silica gel column chromatography (petroleum ether/ethyl acetate/methanol/ammonia 32% aqueous solution,6:4:0.5:0.05) yielded **39** as a yellow solid (290 mg, 16%). ^1^H NMR (400 MHz, CDCl_3_) δ 7.95–7.89 (m, 2H), 7.25 (t, *J* = 7.3 Hz, 2H), 7.19–7.11 (m, 5H), 7.06–6.96 (m, 5H), 6.27 (d, *J* = 3.4 Hz, 1H), 6.19 (t, *J* = 5.9 Hz, 1H), 4.83 (s, 2H), 3.58 (dd, *J* = 13.4, 7.0 Hz, 2H), 3.28–3.19 (m, 4H), 2.84 (t, *J* = 7.2 Hz, 2H), 2.63–2.56 (m, 2H), 2.31–2.24 (m, 2H), 2.25–2.18 (m, 4H), 1.74 (dd, *J* = 15.1, 7.8 Hz, 2H), 1.58 (s, 9H). LCMS (ESI): Calcd for C_39_H_46_FN_4_O_5_ [M + H]^+^*m/z*: 669.35; found, 669.40.

### 5-(Aminomethyl)-*N*-(4-fluorophenethyl)furan-2-carboxamide (**40**)

A mixture of alkyl halide **38** (200 mg, 0.710 mmol) and potassium phthalimide salt (158 mg, 0.852 mmol) in dimethylformamide (3 mL) was heated to 60 °C for 3 h. The reaction mixture was poured into water and extracted with ethyl acetate (3 × 15 mL). The organic phases combined, were filtered over Na_2_SO_4_, and concentrated *in vacuo* (281 mg, 100%). After the evaporation of the solvent, the intermediate 5-((1,3-dioxoisoindolin-2-yl)methyl)-*N*-(4-fluorophenethyl)furan-2-carboxamide (281 mg, 0.71 mmol) was dissolved in ethanol (8 mL), and a solution of hydrazine hydrate (0.70 mL, 14.33 mmol) was added dropwise. The reaction was refluxed for 4 h, ethanol was removed *in vacuo*, and the residue was washed with dichloromethane. The solid is filtered off, and the organic phases are combined and washed with water (2 × 10 mL). The crude produce was purified by column chromatography eluting with dichloromethane/methanol, 9.5:0.5, to obtain **40** as a colorless oil (4.1 mg; 56% yield). ^1^H NMR (400 MHz, CD_3_OD) δ 7.24 (dd, *J* = 8.5, 5.5 Hz, 2H), 7.08 (d, *J* = 3.5 Hz, 1H), 7.04–6.95 (m, 2H), 6.67 (d, *J* = 3.5 Hz, 1H), 4.22 (s, 2H), 3.61–3.52 (m, 2H), 2.91–2.85 (m, 2H). LCMS (ESI): Calcd for C_14_H_16_FN_2_O_2_ [M + H]^+^*m/z*: 264.12; found, 264.32.

### Phenyl ((5-((4-Fluorophenethyl)carbamoyl)furan-2-yl)methyl)carbamate (**41**)

Phenyl chloroformate (**27**, 60 mg, 0.38 mmol, 1.0 equiv) was added dropwise to a solution of **40** (0.35 mmol, 105 mg, 1.0 equiv) and K_2_CO_3_ (0.21 mmol, 29 mg, 0.6 equiv) in a mixture of ethyl acetate, tetrahydrofuran, and H_2_O (1 mL, 0.15 and 0.15 mL, respectively) cooled at 0 °C. The reaction mixture was stirred at room temperature for 2 h, then it was concentrated *in vacuo* to remove the organic solvents. The reaction crude was purified by column chromatography eluting with dichloromethane/methanol, 9.8:0.2, to afford **41** (58 mg, 44%). ^1^H NMR (400 MHz, CDCl_3_) δ 7.41–7.33 (m, 2H), 7.12 (d, *J* = 8.0 Hz, 2H), 7.05 (d, *J* = 3.3 Hz, 1H), 6.99 (dt, *J* = 8.7, 2.0 Hz, 2H), 6.94–6.87 (m, 1H), 6.86–6.80 (m, 2H), 6.48 (s, 1H), 6.38 (d, *J* = 3.3 Hz, 1H), 5.45 (s, 1H), 4.45 (d, *J* = 6.0 Hz, 2H), 3.64 (dd, *J* = 13.8, 6.7 Hz, 2H), 2.93–2.83 (m, 2H). LCMS (ESI): Calcd for C_21_H_20_FN_2_O_4_ [M + H]^+^*m/z*: 383.14; found, 383.21.

### Computational Studies

The conformational plasticity of the target is only partially taken into account in docking calculations, and the results of these methodologies are often very sensitive to the quality of the input structure. Indeed, after the visual inspection of the experimental binding mode adopted by fragments **1**, **2**, and **3** (PDB-ID 5S9Z, 5SA1, and 5S9W, respectively), we set up a simple procedure to choose the most suited protein structures for performing the docking calculations. A cross-docking exercise was then carried out using the Glide tool from Schrodinger^38^ to validate the ability of the docking protocol to reproduce the available experimental complexes and to find out the structure endowed with the better propensity to reproduce the native binding modes. As a result of the cross-docking, PDB-ID 5S9W was chosen. A cubic grid box (36 Å per side) centered on the catalytic tetrad (E466, H461, C52, and C57), the Z-Site, and the MBS were used (Figure S9), in order to cover the entire volume of the binding cavity. From a molecular standpoint, the Z-Site of *Tb*TR cavity is characterized by three important features: (i) a negatively charged surface due to two consecutive glutamate residues (E466 and E467) involved in the stabilization of permanents or pH-dependent positive charges; (ii) a hydrophobic narrow channel below the catalytic tetrad, where the aromatic groups can be accommodated; (iii) the presence of the F396, which is involved in the stabilization of aromatic rings through π–π interactions and/or positively charged groups.

Moreover, as described by Fiorillo *et al.*,^18^ two essential water molecules are involved in the hydrogen-bond network between the amidic moiety of fragment **3** and residues from the Z-Site (L399, M400, T463). Considering their important role in the binding pose adopted by the fragments and the presence in all the crystal structures used for the cross-docking procedure, we decided to include them during the calculation.

The design of new derivatives was based on the assumption that the aforementioned key interactions should be preserved by the resulting docking poses of the derivatives (see the Supporting Information for further details).

## Biology

### Expression and Purification

The *Tb*TR and *Li*TR genes were subcloned in a pET28b vector, and the BL21(DE3) *E. coli* strain was transformed with the resulting constructs. The transformed cells were grown at 37 °C, and expression was induced with 1 mM IPTG once the optical density reached 0.5. The cells were incubated 4 h more at 37 °C prior to harvest. Cell pellets were resuspended in 20 mM Tris pH 8.0, 300 mM NaCl, 5 mM imidazole, 5 mM MgCl_2_, 0.1 mM phenylmethylsulfonyl fluoride, DNase, and cOmplete antiprotease cocktail tablets. The resuspended cells were sonicated and centrifuged to discard the cell debris. The protein was purified by immobilized metal affinity chromatography (IMAC) (Ni-NTA HiTrap column purchased from Cytiva) and eluted using a gradient of imidazole. The 6-His tag was cleaved using 1 unit of thrombin per mg of protein. Thrombin was removed upon binding on a benzamidine sepharose 6B resin (purchased from Cytiva). The tag-free protein was further purified by reverse-IMAC. The buffer was exchanged into 20 mM *N*-(2-hydroxyethyl)piperazine-*N*′-ethanesulfonic acid (HEPES) pH 7.4.

### Enzymatic Assays

Enzymatic inhibition assays were performed in 50 mM HEPES pH 7.4, 40 mM NaCl at 25 °C using a JASCO V650 spectrophotometer equipped with a JASCO EHC 716 Peltier element to ensure controlled temperature. The first experiment was carried out using 50 nM *Li*TR, 10 or 100 μM compound to be tested, 150 μM trypanothione (trypanothione disulfide purchased from Bachem), and 100 μM NADPH (tetrasodium salt, purchased from Calbiochem). The best inhibitor candidates were then tested at concentrations ranging from 1 nM to 250 μM to determine the IC_50_. Assays were initiated upon addition of 100 μM NADPH to 50 nM *Li*TR or *h*GR (human glutathione reductase purchased from Sigma-Aldrich) in the presence of different concentration of compounds and 150 μM trypanothione. For both experiments, the oxidation of NADPH was followed as a decrease in absorbance at 340 nm. For each concentration of compound, the initial velocity of the NADPH oxidation was used to determine the percentage of *Li*TR activity with respect to that in the absence of any compound. The IC_50_ was determined upon fitting the residual activity of TR as a dose–response logistic equation defined as *y*min + (*y*max – *y*min)/(1 + (*x*/IC_50_)^slope). In order to determine the inhibition constant *K*_i_, inhibition kinetics were followed, at 25 °C in the presence of 100 μM NADPH, with compound concentrations ranging from 0 to 15 μM and repeated at four distinct trypanothione concentrations, namely, 0, 25, 50, and 100 μM. The *K*_i_ was determined graphically upon linear fitting of data points for each trypanothione concentration. Data analysis was performed with QtiPlot 0.9.8.9 svn 2288, and graphs were made with Matplotlib.

### Crystallization and X-ray Diffraction

*Tb*TR (12–18 mg/mL) with 50 mM NaBr crystallized in sitting drops using the vapor diffusion method in 13–15% PEG3350, 22–24% MPD, 40 mM imidazole pH 7.5. Crystals were soaked with 0.5–1.7 mM compounds (5% DMSO) from 2 to 12 h. *Tb*TR crystals were mounted on cryo-loops and directly flash-frozen in liquid nitrogen prior to data collection. Diffraction data were collected at Elettra XRD2 beamline (Trieste, Italy) at 100 K at a 1 Å-wavelength on a Pilatus 6 M detector. The data were indexed, integrated, and scaled using XDS^39^ and Aimless.^40^ The structures were solved by molecular replacement using the 2WOI PDB entry as a model in MOLREP 11.7.03.^41^ Iterative rounds of refinement and model building were carried out using Refmac5 5.8.0267 and coot 0.8.9.2.^42−44^ Aimless, MOLREP, and Refmac5 were operated from ccp4 (ccp4 7.1.018). The structures were deposited in the Protein Data Bank as the 8PF3, 8PF4, and 8PF5 entries. Crystallographic details and statistics are reported in Table S2. Images have been prepared with UCSF CHIMERA 1.12.

### Primary Screening Assay

#### *In Vitro* Assay: Inhibition of Axenic Amastigote Growth

Axenic amastigote growth inhibition was evaluated using *L. infantum* strain (MHOM/TN/80/IPT1, WHO international reference strain). Axenic amastigote cultures were obtained as described previously with modifications.^45^ Promastigotes were grown in Schneider’s Drosophila medium (SIGMA) containing 10% heat-inactivated fetal calf serum (FCS)(GIBCO-BRL) and 2% gentamicin (50 mg/L)(Sigma) in 25 cm^2^ flasks at 22 °C. After 4–5 days, the parasites were adjusted to 1 × 10^6^ parasites/mL in axenic medium MAA/20 consisting of modified medium 199 (Gibco BRL) with Hanks’ salts supplemented with 0.5% tryptic soy broth (Sigma), 0.01 mM bathocuproine disulfonic acid, 3 mM l-cysteine, 15 mM d-glucose, 5 mM l-glutamine, 4 mM NaHCO_3_, 0.023 mM bovine hemin, and 25 mM HEPES to a final pH of 6.5 and supplemented with 20% pretested FCS, and axenic amastigotes were obtained by shifting the incubation conditions: 198 μL of suspension was seeded in triplicate in 96-well flat bottom microplates and incubated at 37 °C, 5% CO_2_ for 48 h in order to allow the axenization. The amastigote cells were visualized under the light microscope to confirm transformation. Then, 2 μL of the selected compounds with varying concentrations were added: 100, 50, 25, 12.5, 6.25, 3.12, 1.56, 0.8, 0.4, 0.2, 0.1, 0.05 μM. Amphotericin B (IC_50_ 0.5 μM) (Euroclone) was used as the control. Each experiment was conducted in triplicate for each drug concentration, and three independent experiments were performed. To estimate the 50% inhibitory concentration (IC_50_), the (3-[4.5-dimethylthiazol-2-yl]-2.5-diphenyltetrazolium bromide) (MTT) micromethod was used throughout the experiments with modification. After 72 h of incubation, 30 μL of MTT was added to each well, and the plates were further incubated for 3 h. The absorbance at 550 nm was measured with a 96-well scanner. Antileishmanial activity was expressed as the percentage of inhibition in the number of live parasites compared to that in the control (nontreated parasites). % inhibition was calculated as follows: % *growth inhibition* = [(*absorb cell with drug* × 100)/*absorb cell control*] – 100. Results were analyzed by GraphPad Prism 5.0, dose–response curves were obtained and IC_50_ deduced (GraphPad Software Inc., San Diego, USA).

### Secondary Screening Assay

#### Cytotoxicity Assay

To assess the cytotoxicity of compounds on mammalian macrophages, we tested all the compounds against both human leukemia monocyte cell line (THP-1 cells, ATCC) differentiated into macrophages by treatment with 40 ng/mL of phorbol myristate acetate (PMA; Sigma) for 48 h and peritoneal exudate macrophages harvested from Balb/c mice. Macrophages were placed on 96-well culture plates at 5 × 10^5^ cells/well and incubated with complete RPMI 1640 containing 10% FCS (GIBCO-BRL) in a 5% CO_2_ incubator at 37 °C for 5 h in order to achieve cell adhesion. After this time, the macrophages were incubated with the medium alone (control) or with medium containing different concentrations (2-fold serial dilution from 150 to 0.1 μM) of the selected compounds for 72 h at 37 °C in 5% CO_2_. Cell viability was evaluated using the MTT assay, and the concentration of the compound that produced a 50% reduction of cell viability in treated culture cells with respect to untreated ones (CC_50_) was determined by GraphPad Prism 5.0 (GraphPad Software Inc., San Diego, USA). The data were analyzed statistically by means of Student’s *t*-test using GraphPad Prism 5 software (GraphPad Software, San Diego, CA, USA), *p* values of 0.05 or less were considered statistically significant. The selectivity index for each compound was calculated as the ratio of cytotoxicity (CC_50_) in macrophages cells to activity (IC_50_) against *Leishmania* axenic amastigotes. The experiments were performed in triplicate, and two independent experiments were conducted.

### *Ex Vivo* Experiments

#### Inhibition of Intramacrophage Amastigote Growth

Balb/c murine macrophages were used to perform *ex vivo* assay since the parasites infect them more effectively. Macrophages (5 × 10^5^/well) were placed on glass coverslips within a 24-well culture plate and incubated with complete RPMI in a 5% CO_2_ incubator at 37 °C for 4 h to achieve cell adhesion. After this time, *L. infantum* amastigotes from spleens of previously infected hamsters were added onto the macrophages adhering to the coverslips at the ratio of 10:1 (parasites/macrophages). After 24 h of infection, the noninternalized amastigotes were removed by washes with RPMI. The infected macrophages were incubated with complete RPMI medium (control) or medium containing different concentrations of compounds in a 5% CO_2_ incubator at 37 °C for 72 h. Then, the coverslips were fixed with methanol, stained with Giemsa 10%, and analyzed by optical microscopy. The percentage of the infected macrophages and the number of amastigotes per infected macrophage were determined by random counting of 100 cells in each coverslip. Infection was judged to be adequate if more than 70% of the macrophages in the untreated control were infected. Activity for each compound was expressed as *percentage of parasite burden reduction* = infectivity index of treated cells/infectivity index of untreated cells. The infectivity index was calculated as follows: number of infected macrophages × mean number of amastigotes per macrophage / total number of macrophages. Nonlinear regression analysis (Graph-Pad Software Inc., San Diego, USA) was used for curve fitting and calculation of 50% inhibitory concentrations (IC_50_). The experiments were performed in triplicate, and two independent experiments were conducted.

## Data Availability

Atomic coordinates were deposited on the Protein Data Bank under the accession numbers 8PF3, 8PF4, and 8PF5.

## Supporting Information Available

Chromatograms and MS spectra for the selected compounds; NMR spectra for compounds **6, 9–10**, and **14**; docking calculations; and experimental details for crystallography (PDF)
Molecular formula strings and biological data (CSV)
Coordinates of the docked binding pose of **1** (PDB)
Coordinates of the docked binding pose of **2** (PDB)
Coordinates of the docked binding pose of **3** (PDB)
Coordinates of the docked binding pose of **9** (PDB)
Coordinates of the docked binding pose of **14** (PDB)
Coordinates of the docked binding pose of **5** (PDB)
Coordinates of the docked binding pose of **10** (PDB)
Coordinates of the docked binding pose of **6** (PDB)
Coordinates of the prepared TR_Protein (PDB)

## Supplementary Material

jm3c01439_si_002.csv

jm3c01439_si_003.pdb

jm3c01439_si_004.pdb

jm3c01439_si_005.pdb

jm3c01439_si_006.pdb

jm3c01439_si_007.pdb

jm3c01439_si_008.pdb

jm3c01439_si_009.pdb

jm3c01439_si_010.pdb

jm3c01439_si_011.pdb

## Abbreviations

CF_3_-PTZ: 2-(trifluoromethyl)-10*H*-phenothiazine
CL: cutaneous leishmaniasis
Cl-PTZ: 2-chloro-10*H*-phenothiazine
diClBn: dichlorobenzyl
FBDD: fragment-based drug discovery
*h*GR: human Glutathione Reductase
*Li*: *Leishmania infantum*
MBS: Mepacrine Binding Site
PK: pharmacokinetic
rmsd: root-mean-square deviation
SAR structure-activity relationship SI: selectivity index
*Tb*: *Trypanosoma brucei*
TR: trypanothione reductase
VL: visceral leishmaniasis
WHO: World Health Organization
