# Supplementary material for: Fragment Merging, Growing, and Linking Identify New Trypanothione Reductase Inhibitors for Leishmaniasis
Source: J Med Chem. 2024 Jan 2;67(1):402–19. doi: 10.1021/acs.jmedchem.3c01439 (PMC10788915; doi:10.1021/acs.jmedchem.3c01439)
Supplement: Supplementary file 1 — jm3c01439_si_001.pdf [file jm3c01439_si_001.pdf]

SUPPORTING INFORMATION

Fragment Merging, Growing, and Linking Identify New Trypanothione Reductase Inhibitors for Leishmaniasis

Cécile Exertier,[1,§] Alessandra Salerno,[2,§] Lorenzo Antonelli,[3] Annarita Fiorillo,[3] Riccardo Ocello,[2,4] Francesca Seghetti,[2] Jessica Caciolla,[2] Elisa Uliassi,[2] Matteo Masetti,[2] Eleonora Fiorentino,[5] Stefania Orsini,[5] Trentina Di Muccio,[5] Andrea Ilari,[1]* Maria Laura Bolognesi[2]*

[1] Institute of Molecular Biology and Pathology (IBPM) of the National Research Council of Italy (CNR), c/o Department of Biochemical Sciences, Sapienza University of Rome, Piazzale A. Moro 5, 00185 Roma, Italy

[2] Department of Pharmacy and Biotechnology, Alma Mater Studiorum - University of Bologna, Via Belmeloro 6, 40126 Bologna, Italy

[3] Department of Biochemical Sciences "A. Rossi Fanelli", Sapienza University of Rome, Piazzale A. Moro 5, 00185 Roma, Italy

[4] Computational and Chemical Biology, Istituto Italiano di Tecnologia, via Morego 30, 16163 Genova, Italy

[5] Department of Infectious Diseases, Istituto Superiore di Sanità, Viale Regina Elena 299, 00161 Roma, Italy

Corresponding Authors

andrea.ilari@cnr.it

marialaura.bolognesi@unibo.it

TABLE OF CONTENTS

**Docking calculations of fragments 1-3**	**3**
**Figure S1**	**4**
**Docking studies of fragment-derived 6, 9, 10 and 14**	**5**
**Figure S2**	**6**
**Figure S3**	**7**
**Figure S4**	**8**
**Table S1**	**9**
**Figure S5**	**9**
**Table S2**	**10**
**Figure S6**	**11**
**Figure S7**	**12**
**Figure S8**	**13**
**Figure S9**	**14**
**Figure S10**	**15**
**Compound Purity and HPLC traces**	**16**
**^{1}H-NMR and ^{13}C-NMR spectra**	**18**
**Bibliography**	**22**

Docking calculations of fragments 1-3. In Figure S1, we report the best results in terms of docking score for each fragment together with the respective crystallographic binding mode as a reference. All the docking calculations share the same size and location of the box, as shown in Figure S2. Interestingly, **3** was the only fragment matching the crystallographic binding mode, in terms of the interactions with the Z-Site (π-π stacking with P369, and the water-mediated hydrogen bond between the amidic group and T463, E467, and M400). In particular, the predicted docking pose adopted a kinked conformation, where the *p*-fluoro-phenyl ring was accommodated below the catalytic tetrad, and it was surrounded by lipophilic residues (L399, M400, P462).

Conversely, the best pose predicted for fragment **1** diverged significantly with respect to its crystallographic reference. In particular, the salt bridge between the positively charged piperazine ring and E467 was replaced by an equivalent interaction with nearby E466, and the entire molecule appeared to be shifted toward the solvent-exposed portion of the binding site. Notably, the departure from the Z-site is justified by the establishment of additional hydrophobic interactions of the propyl-benzene tail with a solvent-exposed cluster of lipophilic residues (V362, P336, I339, and I458, among the others).

A remarkable difference between the predicted and experimental binding modes was also observed for fragment **2**. Here, the best docking pose turned out to be highly influenced by the direct hydrogen bond established between the ureidic moiety and L399 and the water-mediated hydrogen bond of the same group with E467 and T463. Furthermore, the positively charged nitrogen of the piperazine ring interacted with E467 and F369 through a salt bridge and cation-π interaction, respectively. Intriguingly, the orientation of the ethyl-*p*-fluorophenyl moiety is overall consistent with that displayed by the same group in fragment **3**, which was located in the hydrophobic cavity, rather than towards the solvent.

We decided to take advantage of the information derived from the crystal structures and docking calculations to merge the fragments, exploiting the common functional group like the amidic core or the piperazine ring as pivotal features for binding the Z-Site.

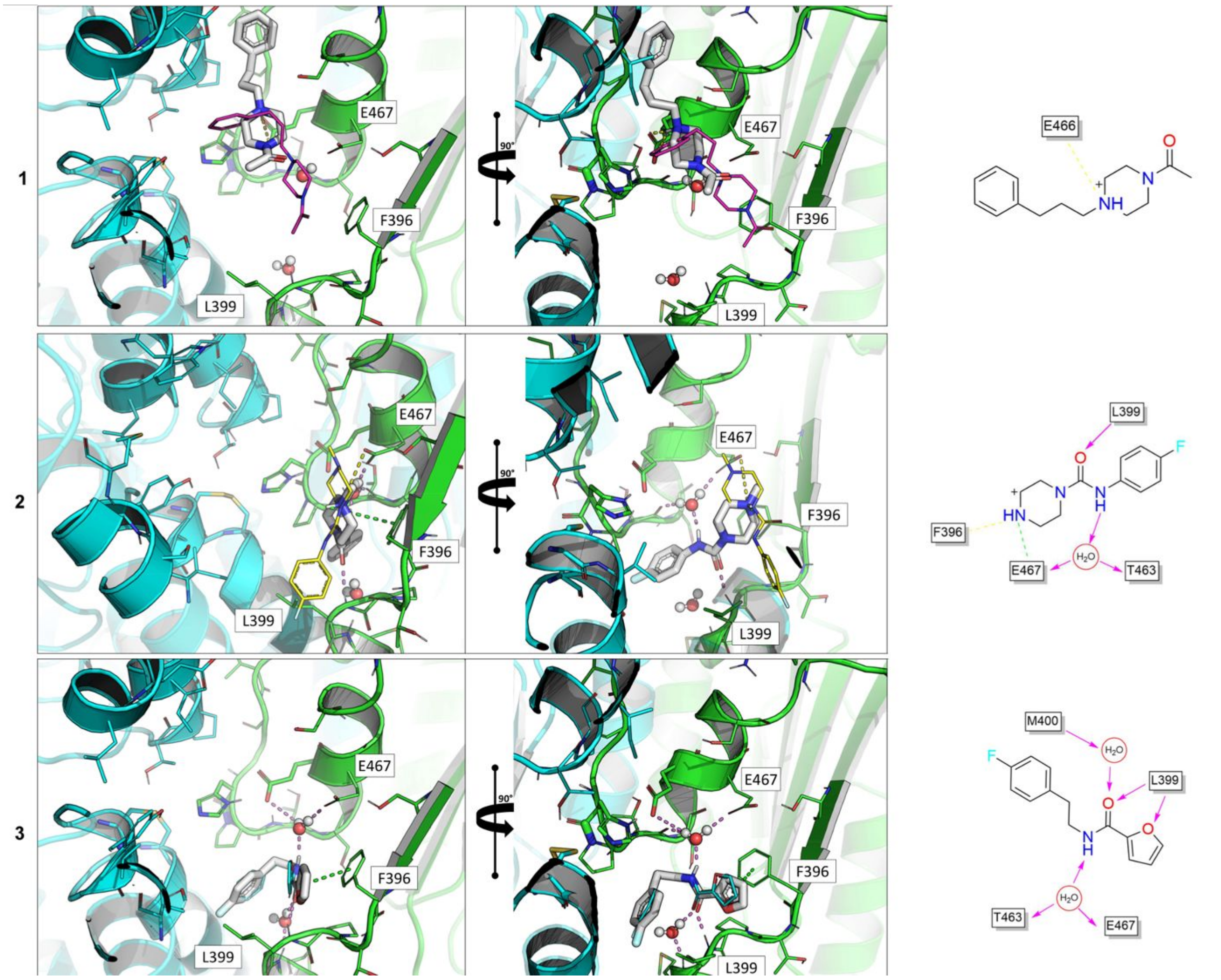

Figure S1. Left: comparison between the binding modes of fragments **1**-**3** as predicted by the docking calculation (thin sticks) and the experimental binding mode (thick white sticks). Right. 2D representation of the main interactions established by the best docking pose for each fragment with TR residues.

Docking studies of fragment-derived compounds 6, 9, 10, and 14. Docking studies were performed for compounds representative of each group of modifications to confirm that the compounds designed by knowledge-guided growing strategy were able to address the desired hotspots while preserving the network of crucial interactions.

Compounds **6-8** were designed to probe modifications of the aromatic region interacting with the MBS in fragment-derived compound **5**, and the binding mode of compound **6** was predicted by docking calculations (Figure S2). In the best ranked pose, the tricyclic scaffold (PTZ) of **6** favourably interacts with the MBS, particularly with H461, T110 and G49. Similarly, the orientation of the furan portion directed to the Z-site is preserved compared to the hit fragment **5**, but the compound loses the interaction with the γ-Glu site.

Computational studies were performed on compounds **9** and **10** bearing *N*-alkylation on the piperazine ring (Figure S2). From docking calculations, the binding mode of **9** seemed preserved compared to **5** upon the introduction of a permanent positive charge by *N*-methylation of piperazine ring. Indeed, the quaternary nitrogen guides the electrostatic recognition with E466. Similarly, we preserved the cationic charge of quaternary nitrogen whilst conferring to the molecule a more hydrophobic surface contact by substitution of the methyl group with a diClBn moiety (**10**). Consistently with the behaviour of **9**, the interactions of **10** resulted preserved in the region of the ethyl-*p*-fluorophenyl and furan ring bound to the Z-site wall, and also regarding the salt bridge with E466. The prediction of the diClBn group contacting the MBS was explained by a series of weak Van der Waals interactions with T110.

As suggested from the analysis of docking pose of hit fragment **5** and crystal structure of **2**, compounds **11-14** were designed by modification on region C. Among those, the docking pose of **14** (Figure S2) confirmed the possibility of removing the hydrophobic *p*-fluorophenyl ring exposed to the solvent without affecting the overall interactions. The conformation adopted by the derivative **14** is indeed similar to that of **10** (analogue, bearing the *p*-fluorophenyl group).

Overall docking studies confirmed that the desired hotspots were addressed by the different strategies of fragments optimization that led to the design of compounds **6-14**. Moreover, in all cases, the interactions with the small cavity targeted by furan-moiety are consistent notwithstanding modifications of the other substituents directed to other hotspots.

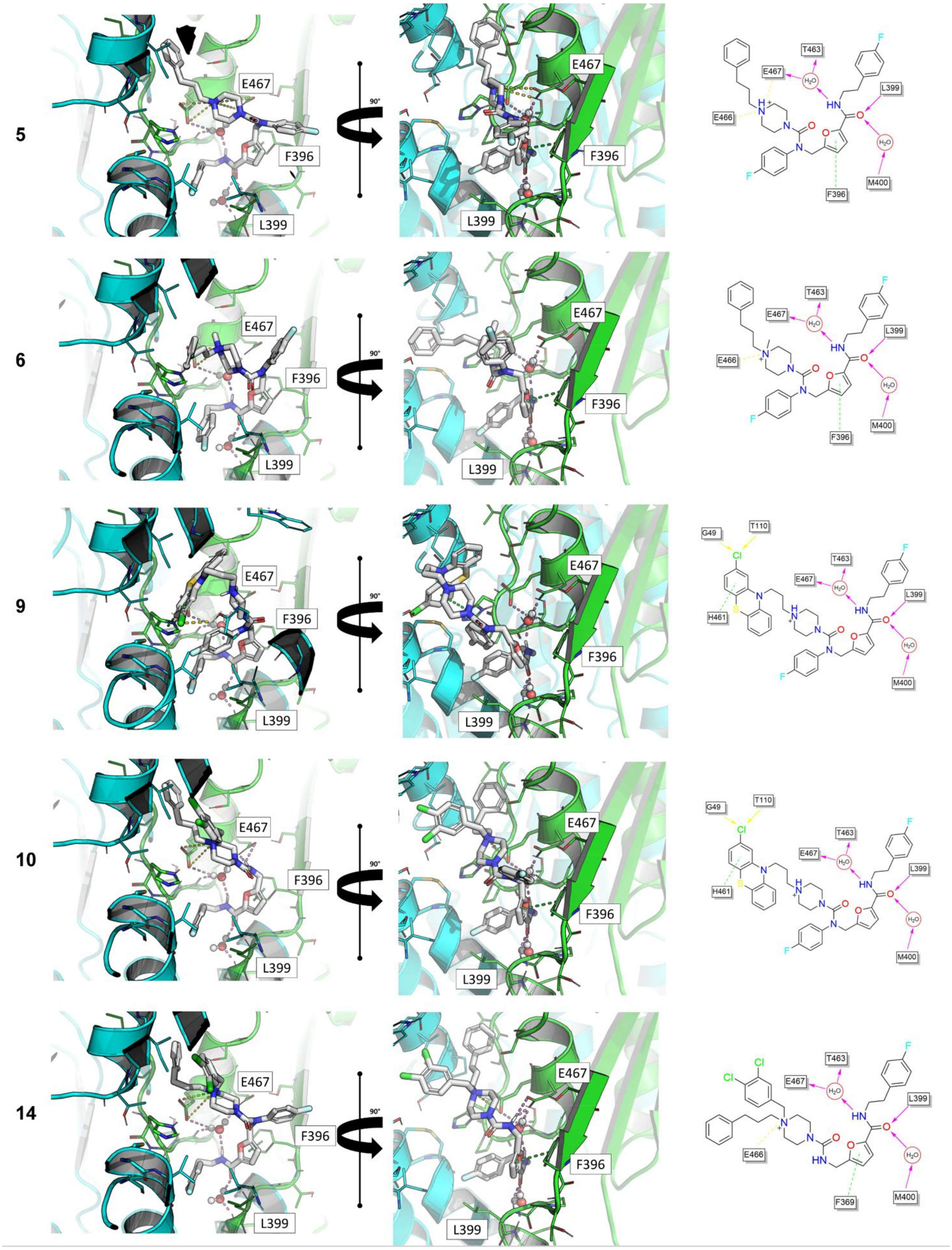

Figure S2. Binding modes of fragments **5**, **6**, **9**, **10**, and **14** as predicted by docking calculation (left) and 2D representations of the main interactions established with TR residues (right).

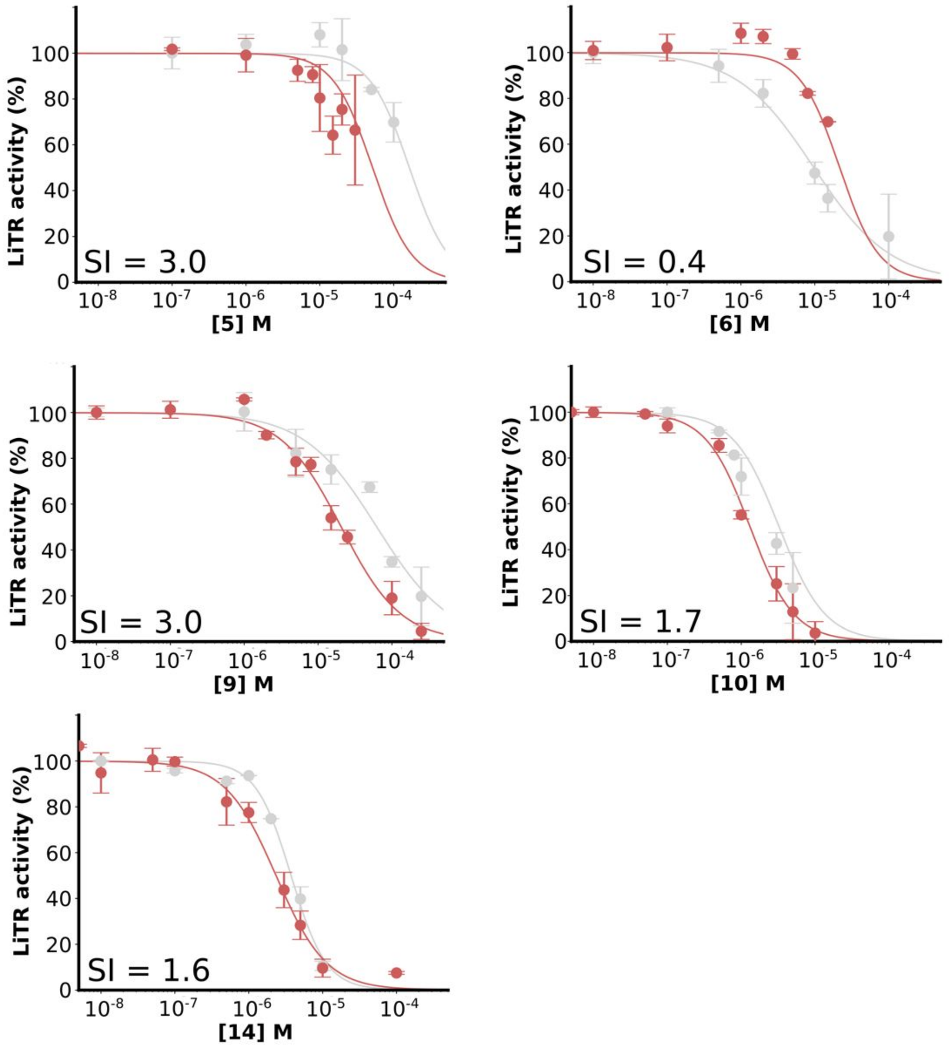

Figure S3. Inhibition assay of *Li*TR (red) and *h*GR (grey) with compounds 5-6, 9-10 and 14. Enzymatic assays were carried out at 25°C on a JASCO V650 spectrophotometer equipped with a JASCO EHC716 Peltier unit. Compound concentrations used during the experiments range from 1 nM to 250 µM. The low solubility of **5** and **6** did not allowed reliable data collection at concentrations higher than 30 µM and 20 µM, respectively. Briefly, 50 nM *Li*TR or *h*GR (Sigma Aldrich) were mixed in 50 mM HEPES pH 7.4, 40 mM NaCl with each compound and 150 µM trypanothione or glutathione. After 3-minute incubation at 25°C, the measurement was initiated upon addition of 100 µM NADPH. The oxidation of NADPH was followed as a decrease in absorbance at 340 nm. Each initial velocity of *Li*TR enzymatic reaction with the compounds was expressed as a percentage of residual activity with respect to that in the absence of compound. Each data point is the mean of two measurements. The IC_{50} was determined upon fitting the data as a dose response logistic equation defined by the Kaleidagraph software as ymin+(ymax-ymin)/(1+(x/IC_{50})^slope).

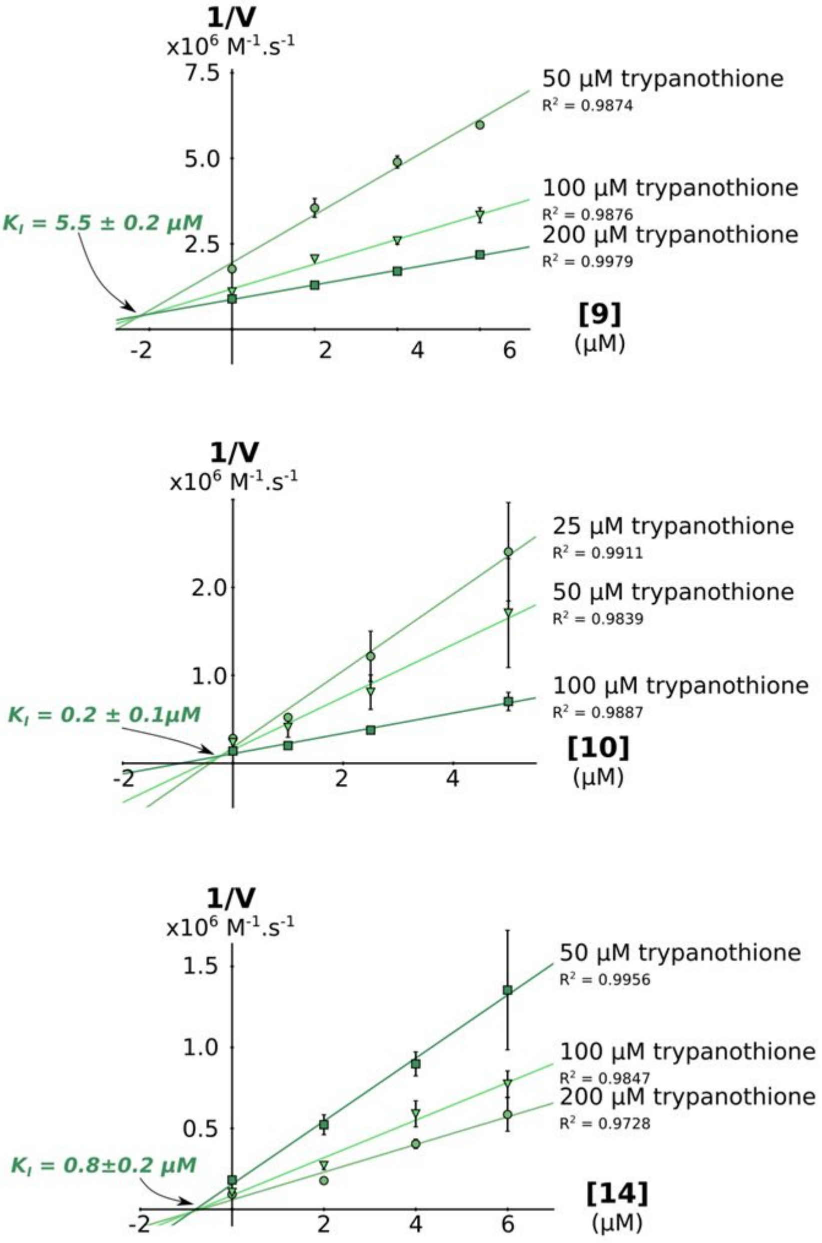

Figure S4. Determination of inhibition constants K_i of *Li*TR towards 9, 10 and 14. Enzymatic assays were carried out at 25°C on a JASCO V650 spectrophotometer equipped with a JASCO EHC716 Peltier unit. Compound concentrations range from 1 to 6 µM. Briefly, 50 nM *Li*TR were mixed in 50 mM HEPES pH 7.4, 40 mM NaCl with each compound and various concentrations of trypanothione (reported on the figure). After 3-minute incubation at 25°C, the measurement was initiated upon addition of 100 µM NADPH. The oxidation of NADPH was followed as a decrease in absorbance at 340 nm. The inverse of the initial velocity of *Li*TR enzymatic reaction was reported as a function of the compound concentration. The intersection of the linear fitting of data at different trypanothione concentration provides a graphical determination of the inhibition constant K_i. Each data point is the mean of three measurements.

Table S1. Calculated physicochemical properties for compounds 1-14. Calculated lipophilicity (Log*P*), Topological Polar Surface Area (*TPSA*), Log*D* at pH=7.40, and *in vitro* TR inhibitory activity.

Cmp.	MW	LogP[a]	TPSA[a]	LogD[b]	LiTR residual activity %	IC_{50} LiTR (μM)
**1**	246.35	2.05	23.55	0.55	77.7 ± 4.8 @100 μM	
**2**	222.26	1.48	23.55	0.90	78.5 ± 7.3 @100 μM	
**3**	233.24	2.56	42.24	2.26	85.3 ± 3.1 @100 μM	
**4**	341.43	3.33	35.58	2.95	96.7 ± 6.1 @100 μM	
**5**	586.68	5.49	69.03	4.81	65.4 ± 3.0 @100 μM	54.6 ± 20.9
**6**	744.3	6.35	97.57	5.65	46.9 ± 18.0 @10 μM	21.7 ± 4.4
**7**	777.86	6.77	97.57	6.03	70.8 ± 5.9 @10 μM	n.d.
**8**	627.51	5.85	69.03	6.03	94.1 ± 4.9 @10 μM	n.d.
**9**	728.62	3.28	65.79	1.41	48.5 ± 7.9 @10 μM	20.5 ± 2.0
**10**	873.61	5.34	65.79	4.34	22.0 ± 11.6 @10 μM	1.31 ± 0.07
**11**	612.7	4.31	106.33	2.31	74.5 ± 9.3 @10 μM	n.d.
**12**	492.6	3.80	77.82	2.73	74.6 ± 9.3 @10 μM	n.d.
**13**	533.43	4.23	77.82	4.01	89.2 ± 4.9 @10 μM	n.d.
**14**	779.52	3.84	74.58	2.32	10.3 ± 2.8 @10 μM	2.35 ± 0.21

[a] Calculated lipophilicity as Consensus *Log*P with Swiss ADME (www.swissadme.ch)
[b] Calculated with Chemaxon's Playground (https://playground.calculators.cxn.io/)

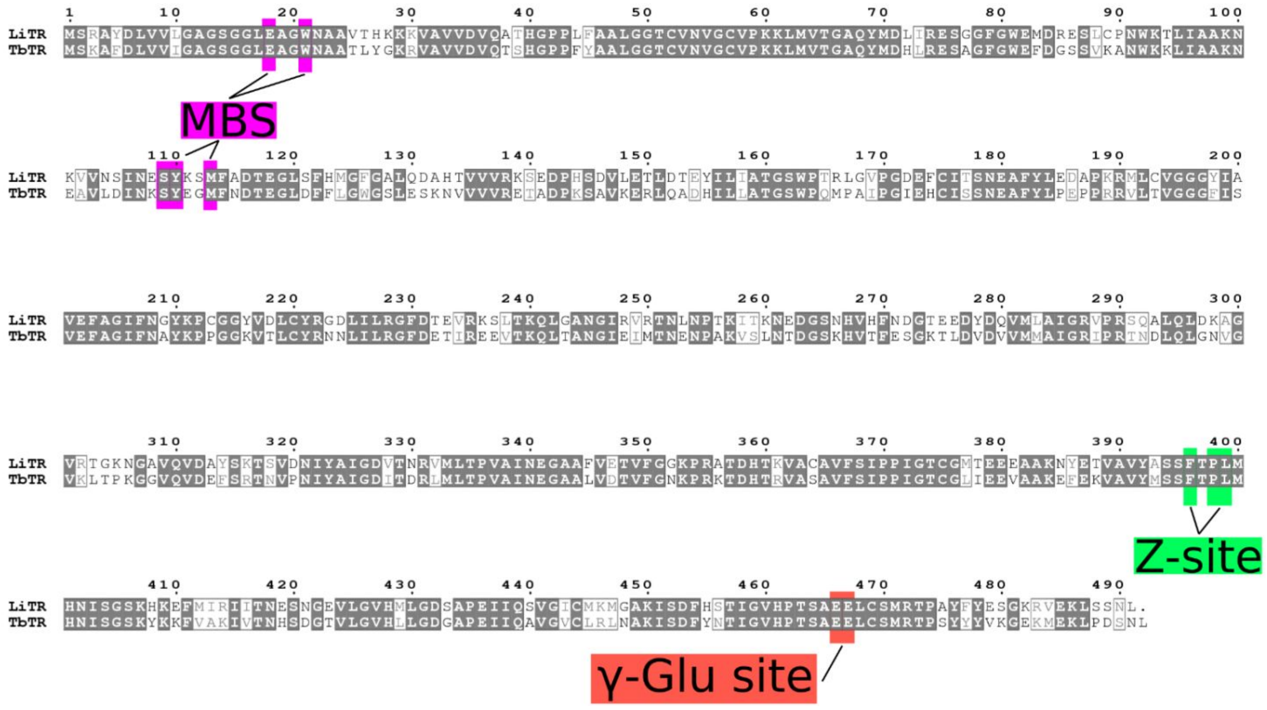

Figure S5. Sequence alignment of *Tb*TR and *Li*TR. The alignment was obtained using the Clustal Omega web tool (https://www.ebi.ac.uk/Tools/msa/clustalo/).[1]

Table S2. Data collections, refinement, statistics, and validation. The statistics for the highest-resolution shell are shown in parentheses. R_{free} is based on 5% of the data randomly selected that were not used during refinement.

	**TbTR-SE13**	**TbTR-ADF01**	**TbTR-AC7**
pdb code	8PF3	8PF5	8PF4
Crystallization conditions	3-15% PEG3350, 22-24% MPD, 40 mM imidazole pH 7.5	3-15% PEG3350, 22-24% MPD, 40 mM imidazole pH 7.5	3-15% PEG3350, 22-24% MPD, 40 mM imidazole pH 7.5
**Data collection**			
Beamline	ELETTRA XRD2	ELETTRA XRD2	ESRF ID23-1
Wavelength(A)	1	1	0.88560
Δφ(°)	0.5	0.5	0.15
Number of images	720	720	2400
Space group	P21	P21	P21
Unit cell parameters (Å/°)	100.70/63.74/170.10	101.11/63.40/169.32	100.92/63.63/169.10
	90.00/97.75/90.00	90.00/98.34/90.00	90.00/97.29/90.00
Resolution range (Å)	168.5-2.15 (2.28-2.15)	167.30-2.42 (2.57-2.42)	167.74 – 1.85 (2.03-1.85)
No of reflexions	708977 (112537)	468306 (76853)	741988 (49946)
No of unique reflections	117489 (18906)	81239 (12995)	135585 (6779)
Completeness (%)	99.7 (98.4)	99.8 (99.5)	94.7 (69.0)
I/sigma (I)	10.37 (2.25)	11.56 (2.08)	8.1 (1.6)
R_{merge}	11.4 (92.5)	12.0 (72.8)	17.6 (208.0)
R_{meas}	12.5 (101.3)	13.2 (80.0)	19.1 (223.7)
$CC_{1/2}$	99.6 (74.1)	99.7 (76.4)	99.4 (65.5)
Mosaicity	0.198	0.382	
**Refinement**			
Wilson B-factors (Å^2)	34.3	41.0	29.6
R_{work}/R_{free}	0.179/0.220	0.190/0.257	0.179/0.230
R.m.s.d. bond length (Å)	0.0083	0.0080	0.0080
R.m.s.d. bond angle (°)	1.5399	1.5700	1.4838
Used Reflections	111276	81177	128789
Number of atoms	16115	15740	17062
Protein	15067	14926	15226
FAD	212	212	212
Compound	208	225	176
Solvent	33	36	262
Waters	595	341	1186
**Mean B factors (Å^2)**			
Protein	43.97	43.59	23.06
FAD	33.30	32.60	13.80
Compound	121.96	65.86	43.48
Solvent (DMSO, PEG)	59.23	61.12	52.58
Waters	40.64	35.90	26.12
**Ramachandran plot statistics**			
Favored (residues/%)	1895/97.4	1870/96.0	1891/97.2
Allowed (residues/%)	48/2.5	71/3.6	50/2.6
Outliers (residues/%)	3/0.3	7/0.4	5/0.3

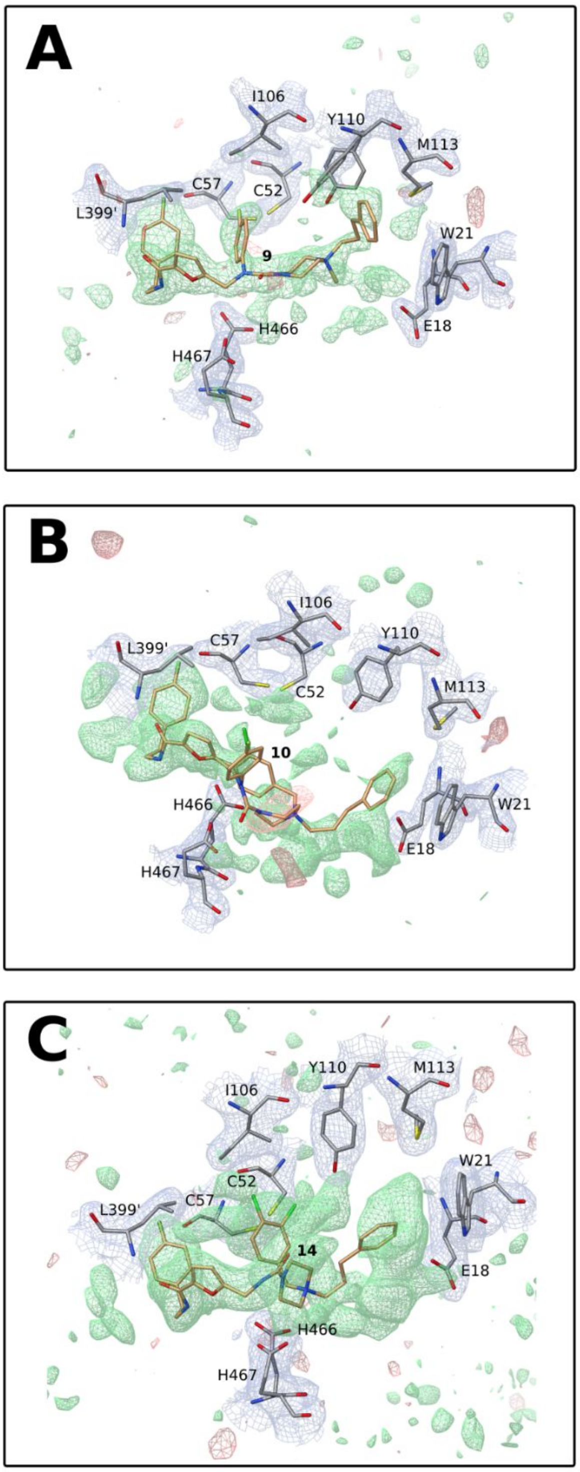

Figure S6. Omit maps. Binding site of TR in complex with compounds **9** (A), **10** (B) and **14** (C). In the figure are represented the 2Fo-Fc maps (blue) contoured at 1σ of TR and Fo-Fc omit maps (green and red) contoured at 3σ of the complexes (in the absence of the compounds). 2Fo-Fc (blue) and Polder Omit Fo-Fc (green and red) maps are contoured respectively at 1 and 3σ. Polder maps make weak densities, that can be obscured by bulk solvent, become visible, which is particularly recommended for ligands.[2]

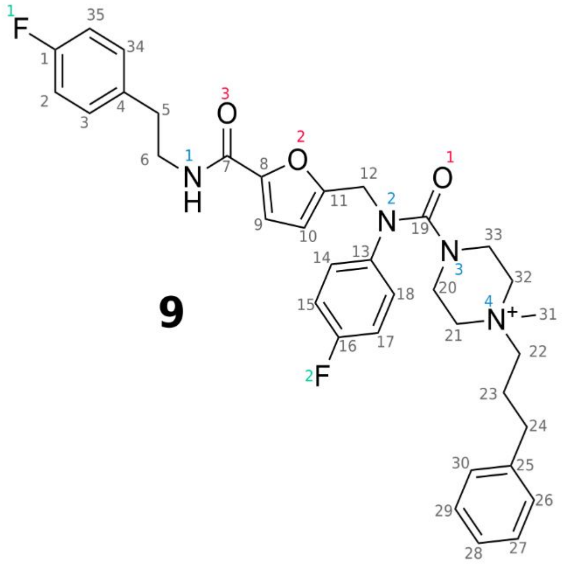

9

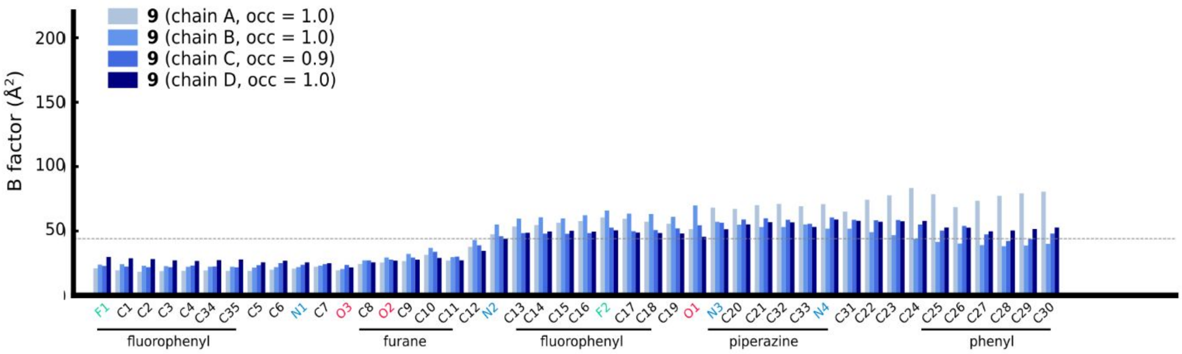

Figure S7. B factors of compound **9**.

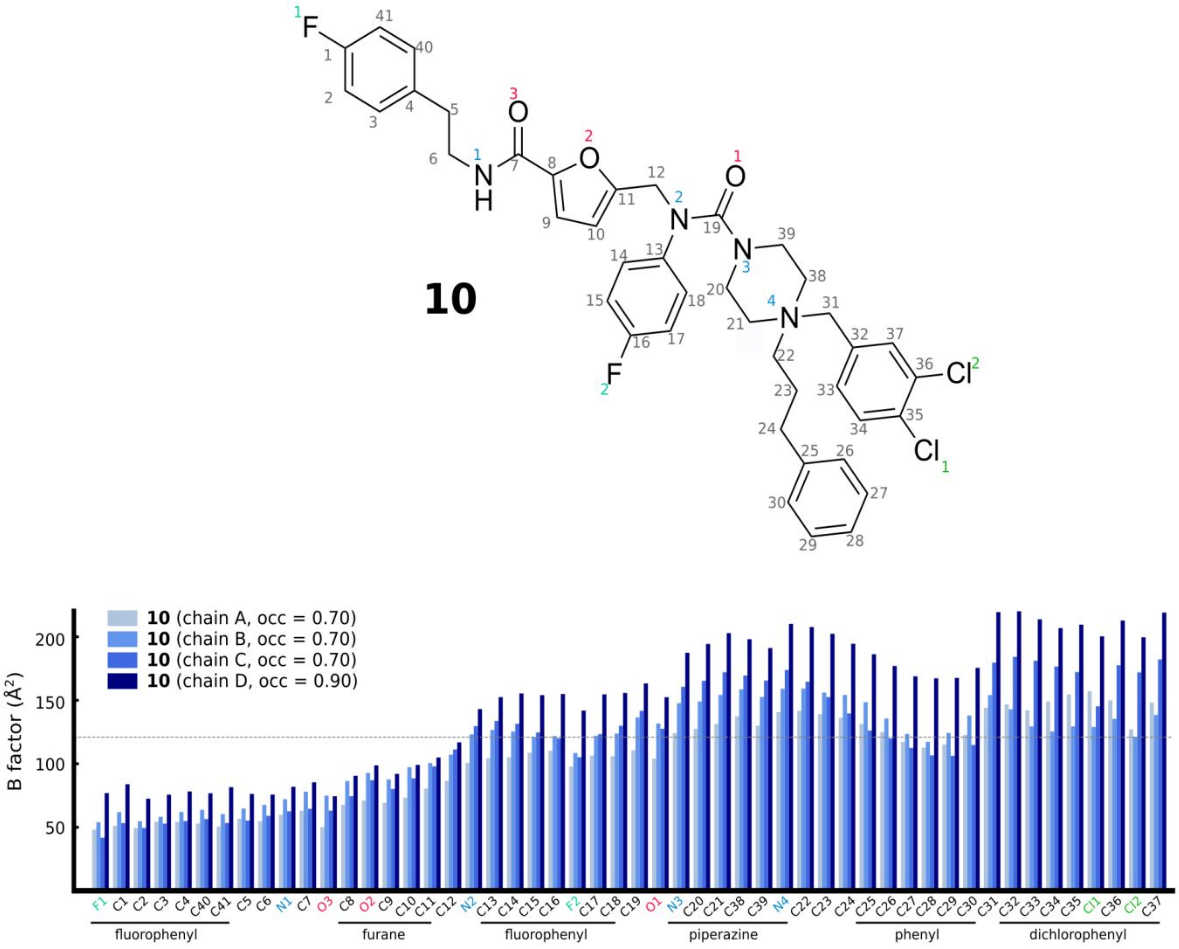

Figure S8. B factors of compound **10**.

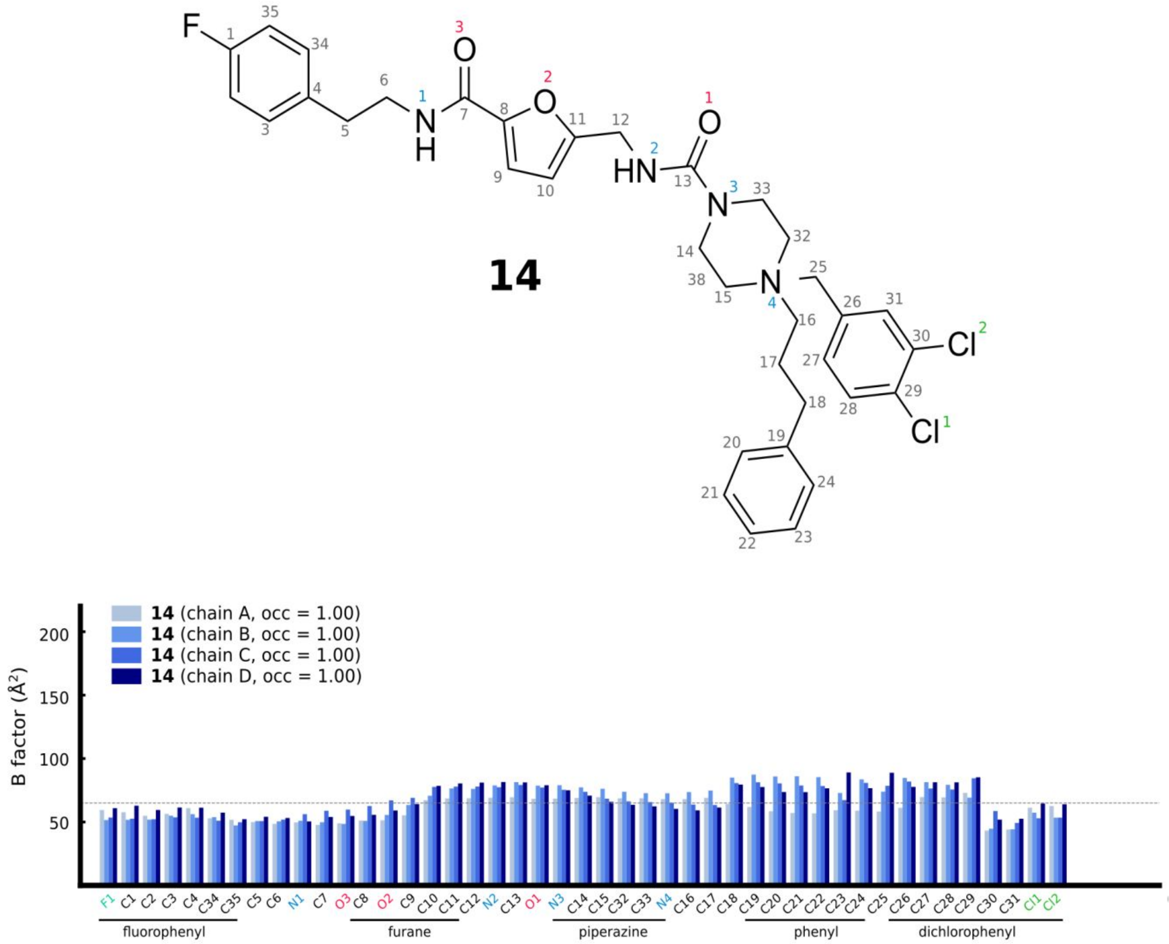

Figure S9. B factors of compound **14**.

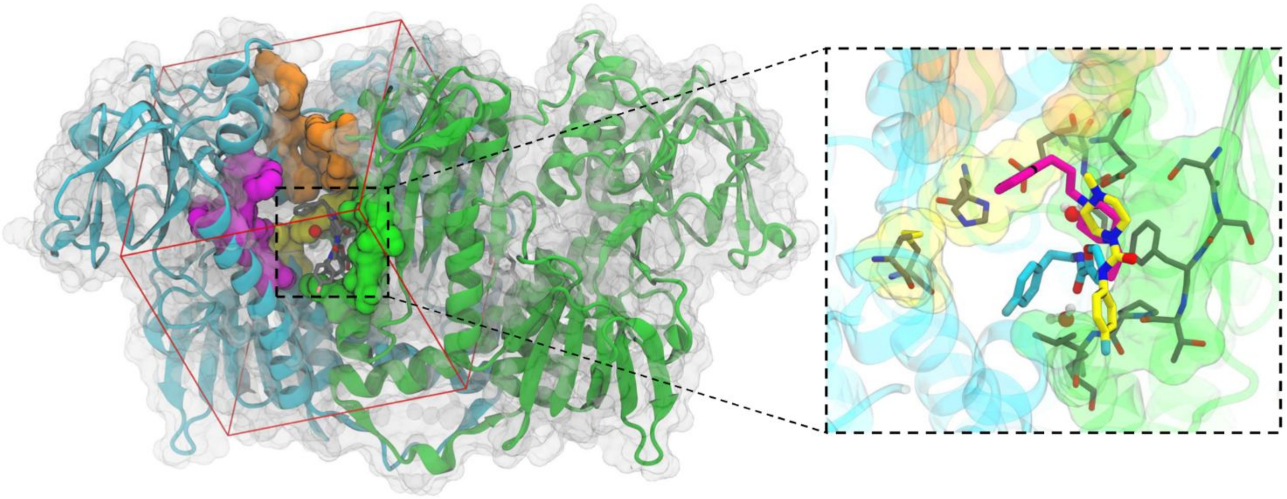

Figure S10. Cartoon representation of TR color-coded in terms of chains A and B (green and cyan, respectively). The residues composing the binding site are displayed as a continuous surface (catalytic site, *Z*-Site, MBS, and the solvent-exposed cluster of hydrophobic residues are shown in yellow, green, pink, and orange). The sides of the box used for setting up the docking calculations are represented in red. The inset shows an overlay of the experimental binding mode of fragments **1**, **2**, and **3** (pink, yellow, and cyan sticks, respectively).

Compound Purity and HPLC traces.

Compound 6

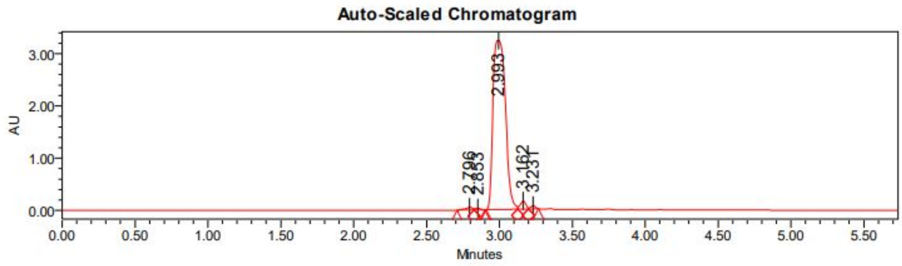

Peak Results

	Name	RT	Area	Height	% Area
1		2.796	176137	52944	0.88
2		2.853	85511	34984	0.43
3		2.993	19188559	3245781	95.94
4		3.162	411022	151617	2.06
5		3.231	138958	60310	0.69

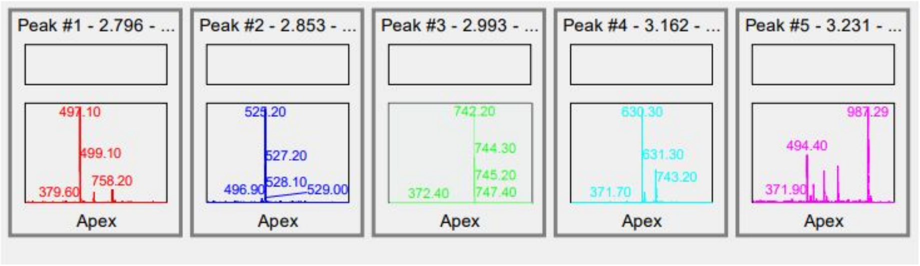

Compound 9

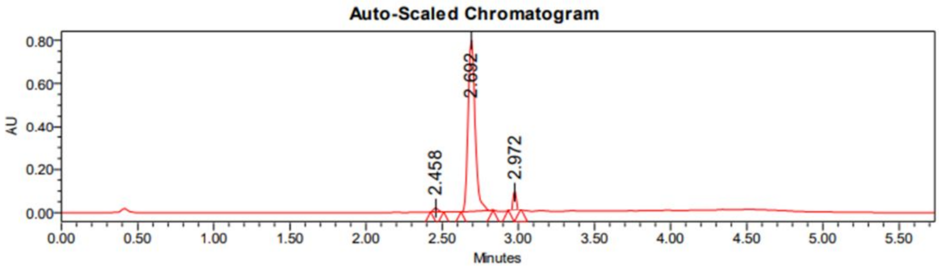

Peak Results

	Name	RT	Area	Height	% Area
1		2.458	38915	17595	1.32
2		2.692	2549139	790804	96.44
3		2.972	66048	29888	2.24

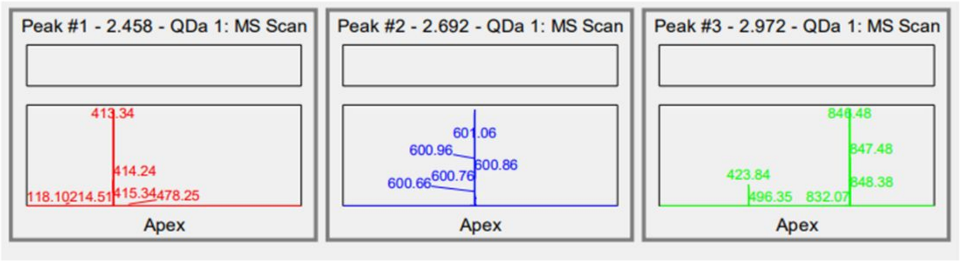

Compound 10

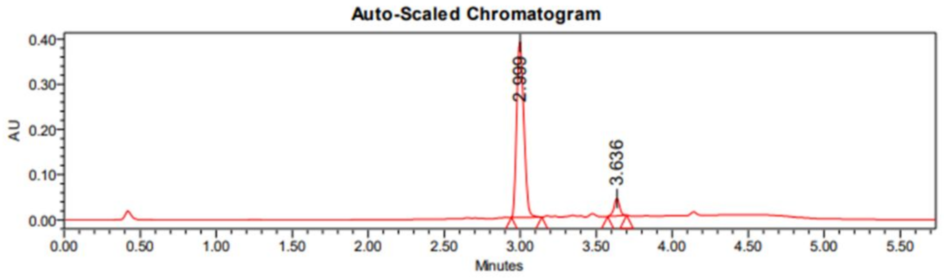

Peak Results

	Name	RT	Area	Height	% Area
1		2.999	1250485	387899	95.62
2		3.636	57673	38154	4.38

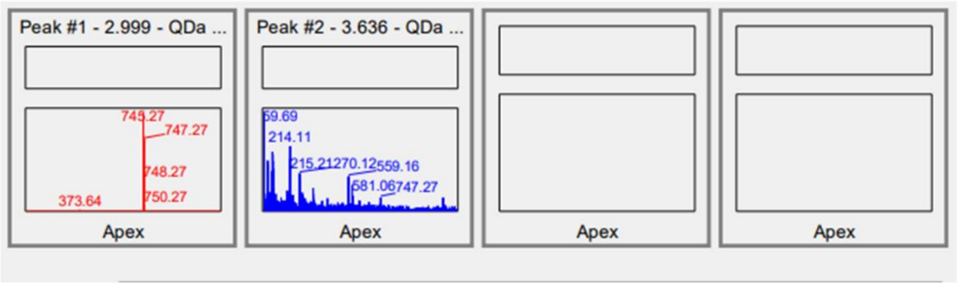

Compound 14

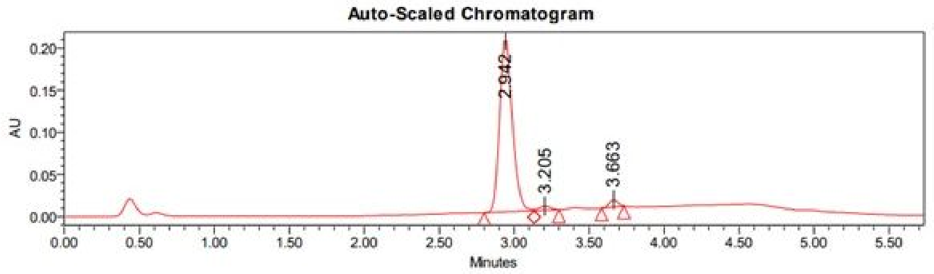

Peak Results

	Name	RT	Area	Height	% Area
1		2.942	1208358	205655	95.13
2		3.205	27058	4937	2.13
3		3.663	34847	8629	2.74

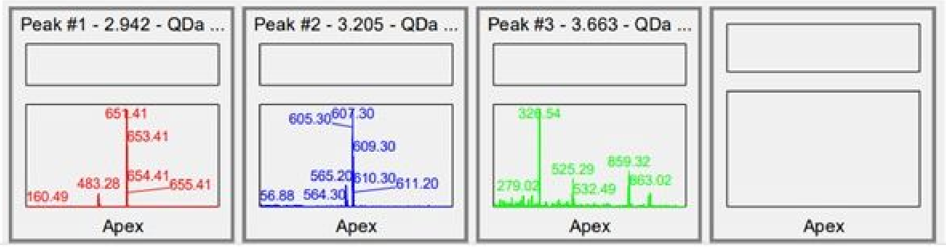

^{1}H-NMR and ^{13}C-NMR of Compound 6 ($CDCl_3$)

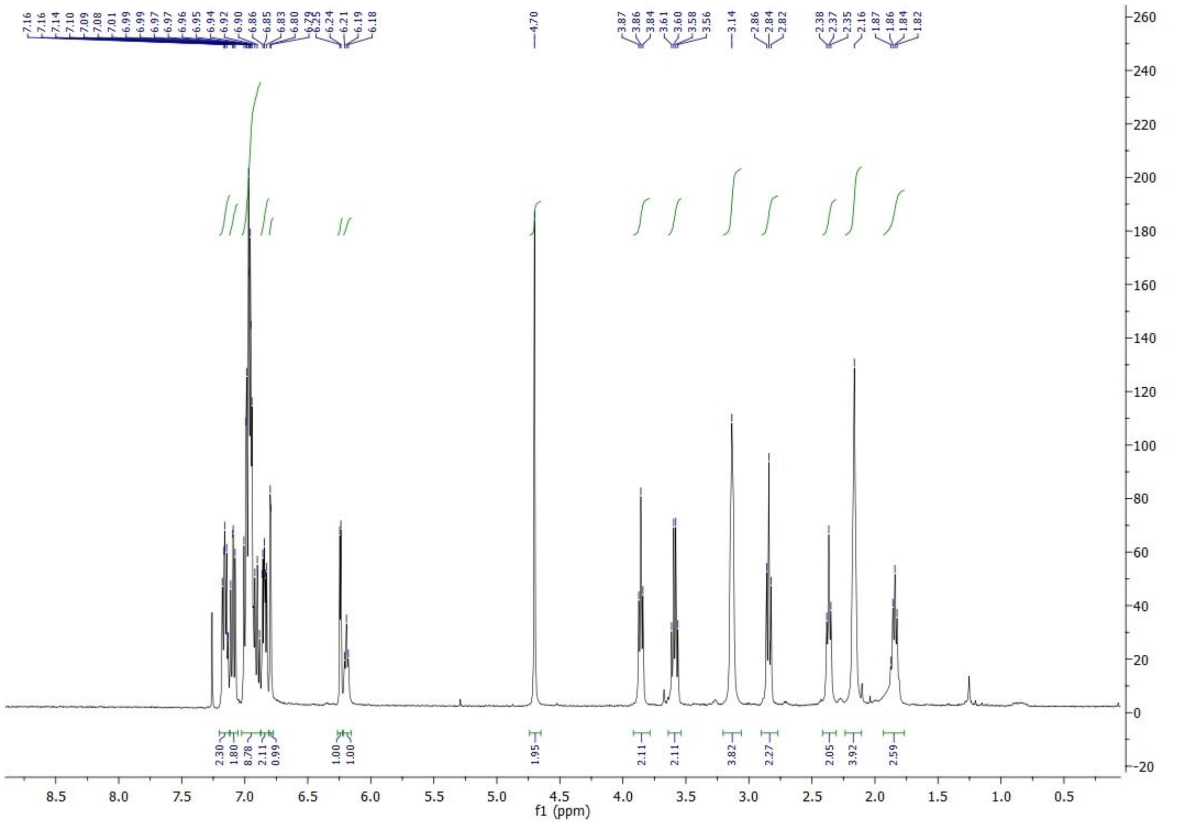

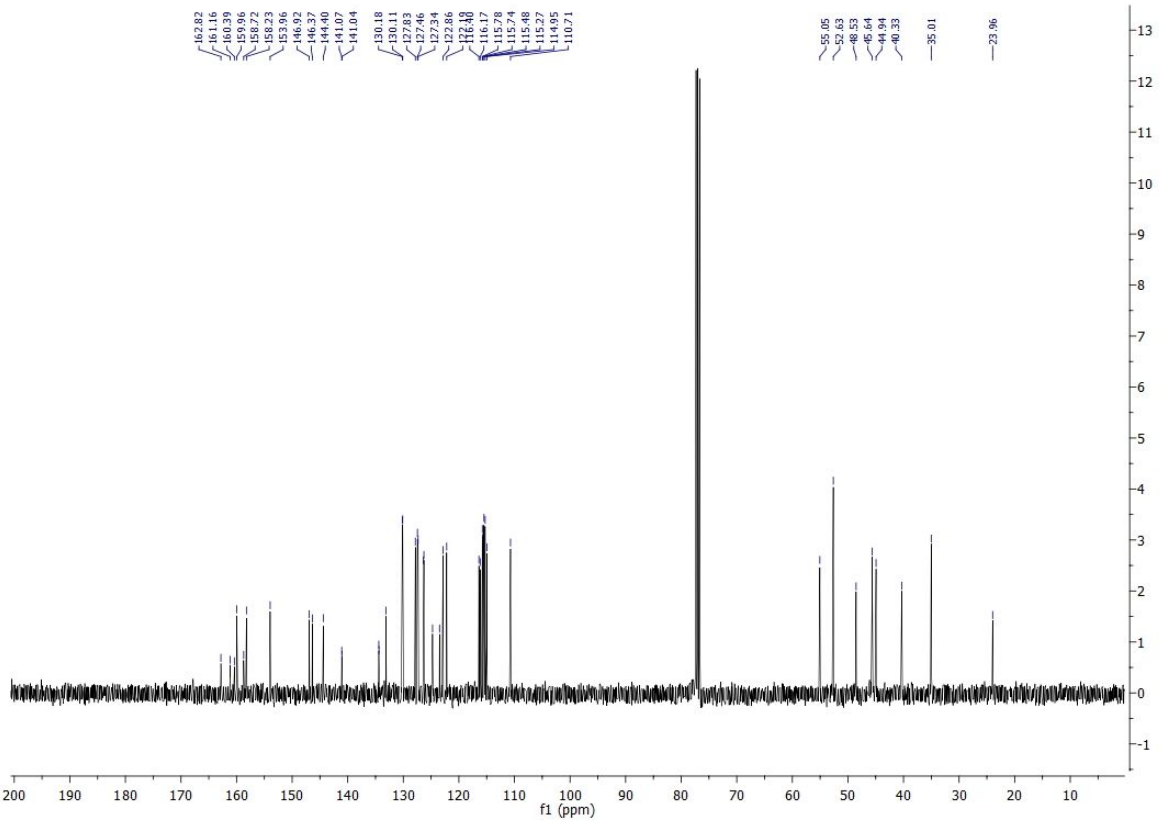

^{1}H-NMR and ^{13}C-NMR of Compound 9 ($CDCl_3$)

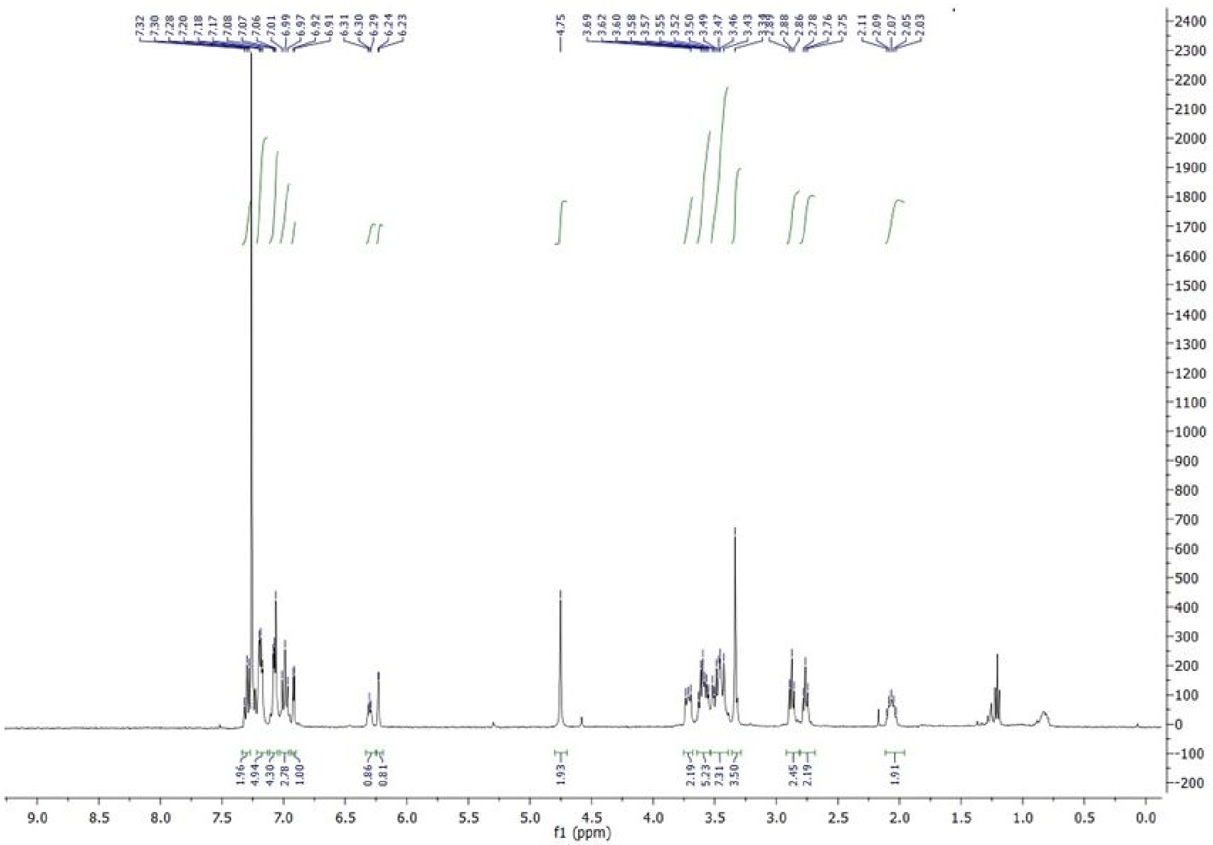

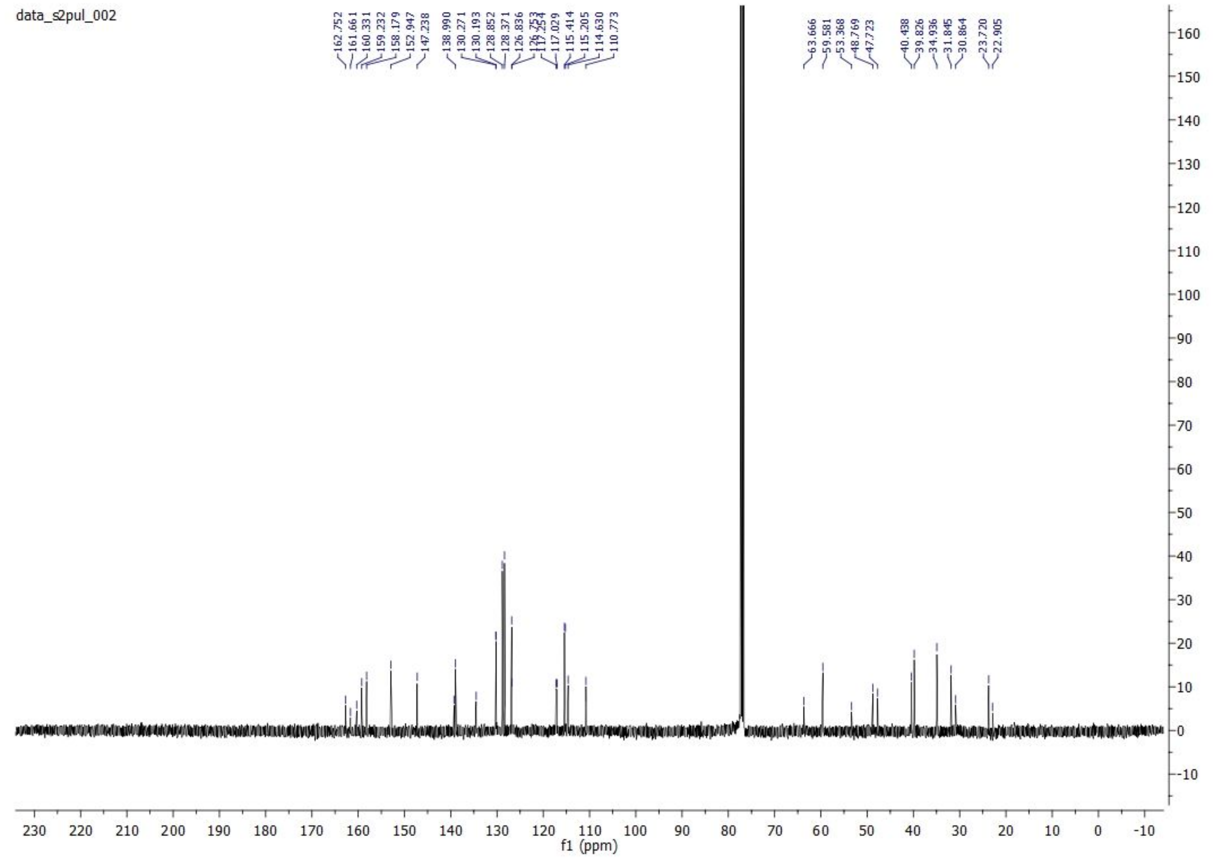

^{1}H-NMR and ^{13}C-NMR of Compound 10 ($CDCl_3$)

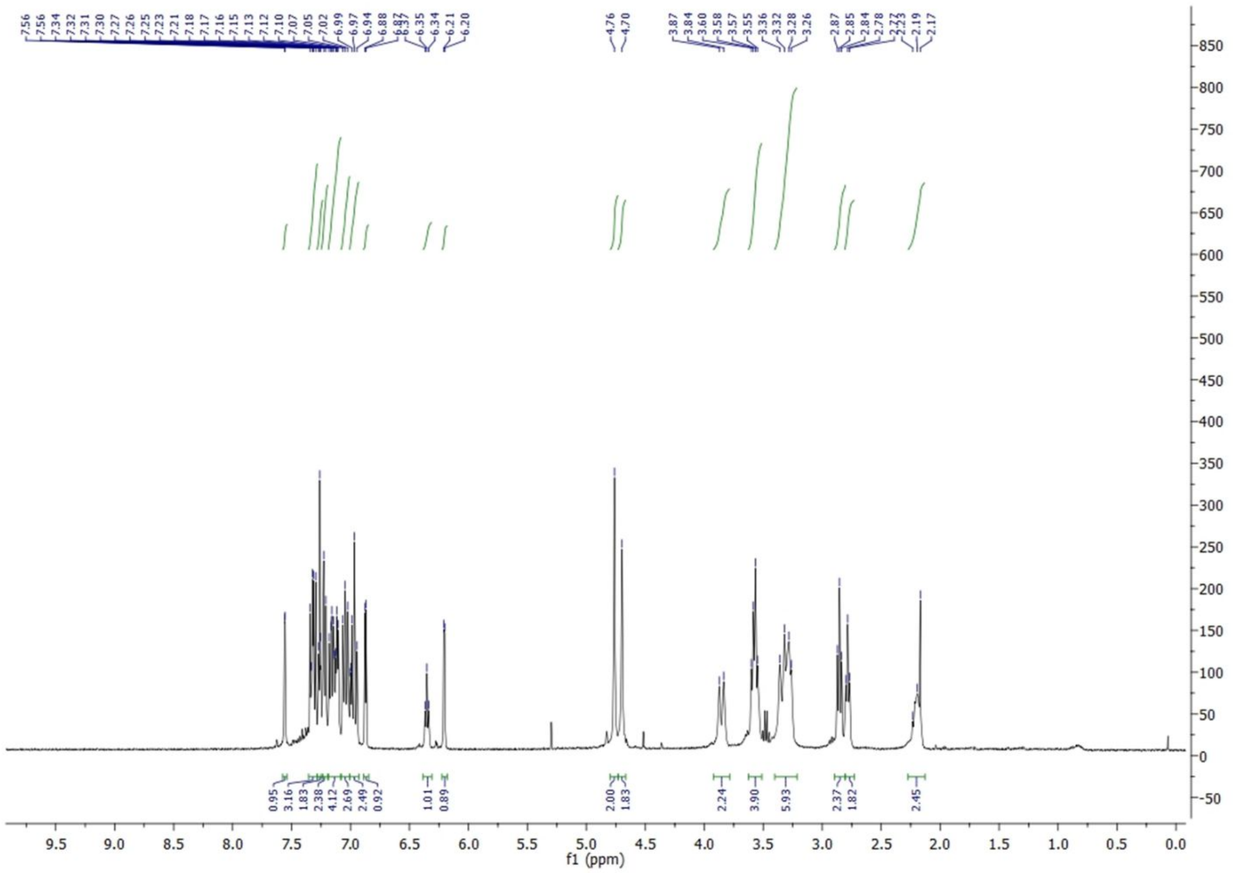

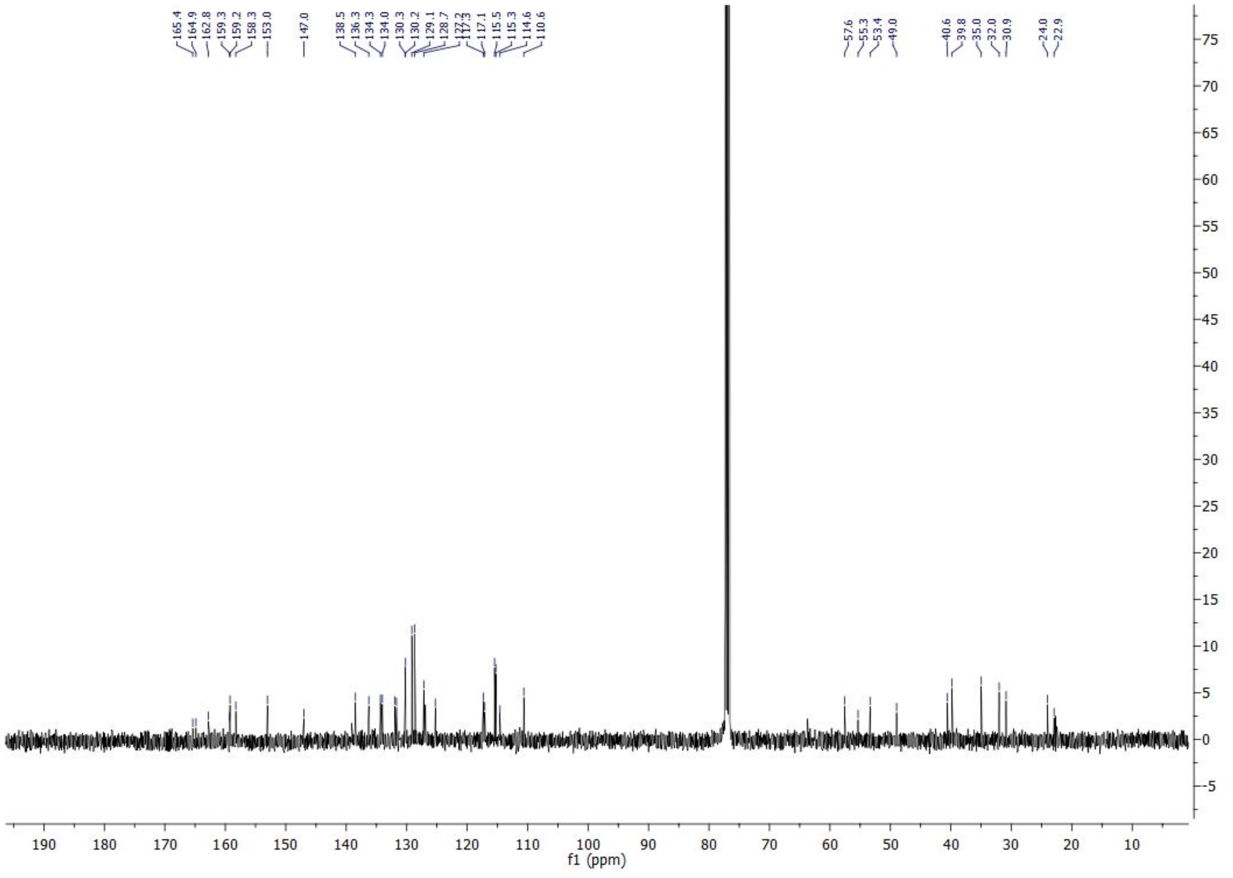

^{1}H-NMR and ^{13}C-NMR of Compound 14 (CD_3OD)

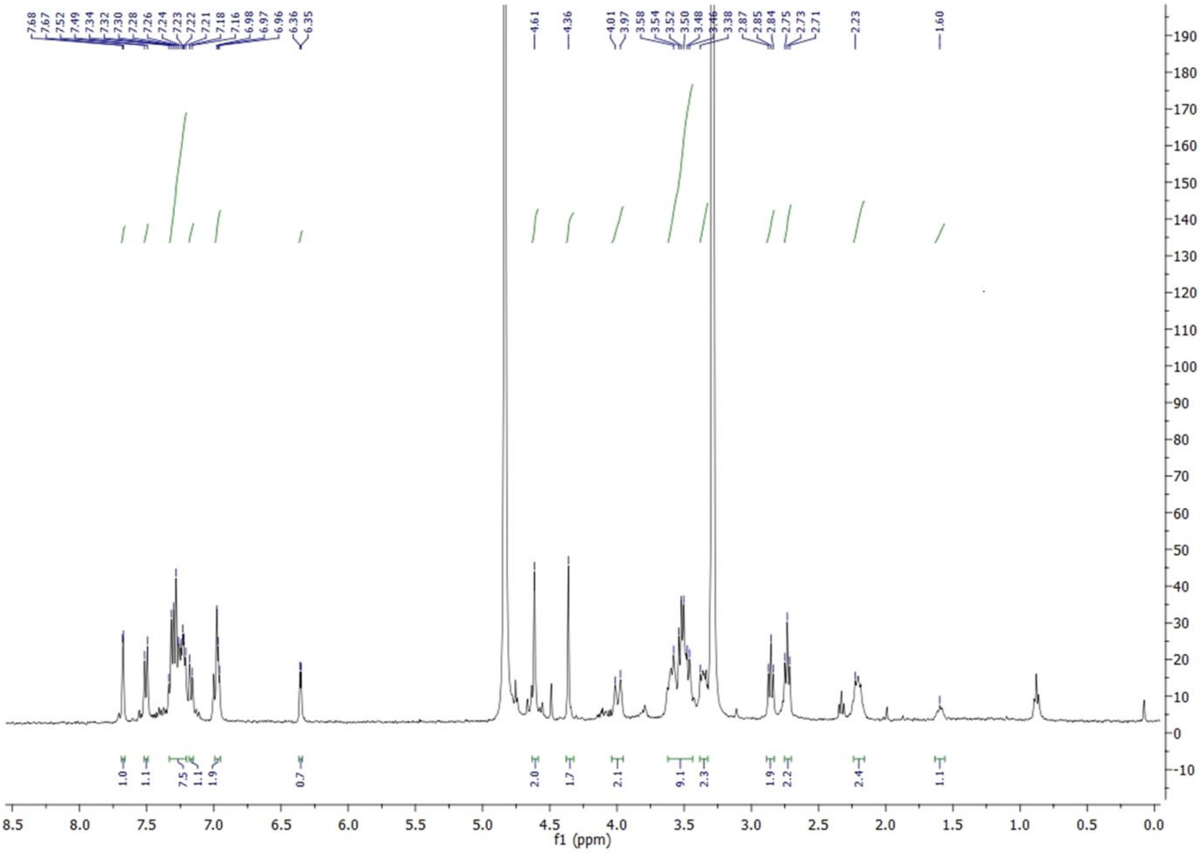

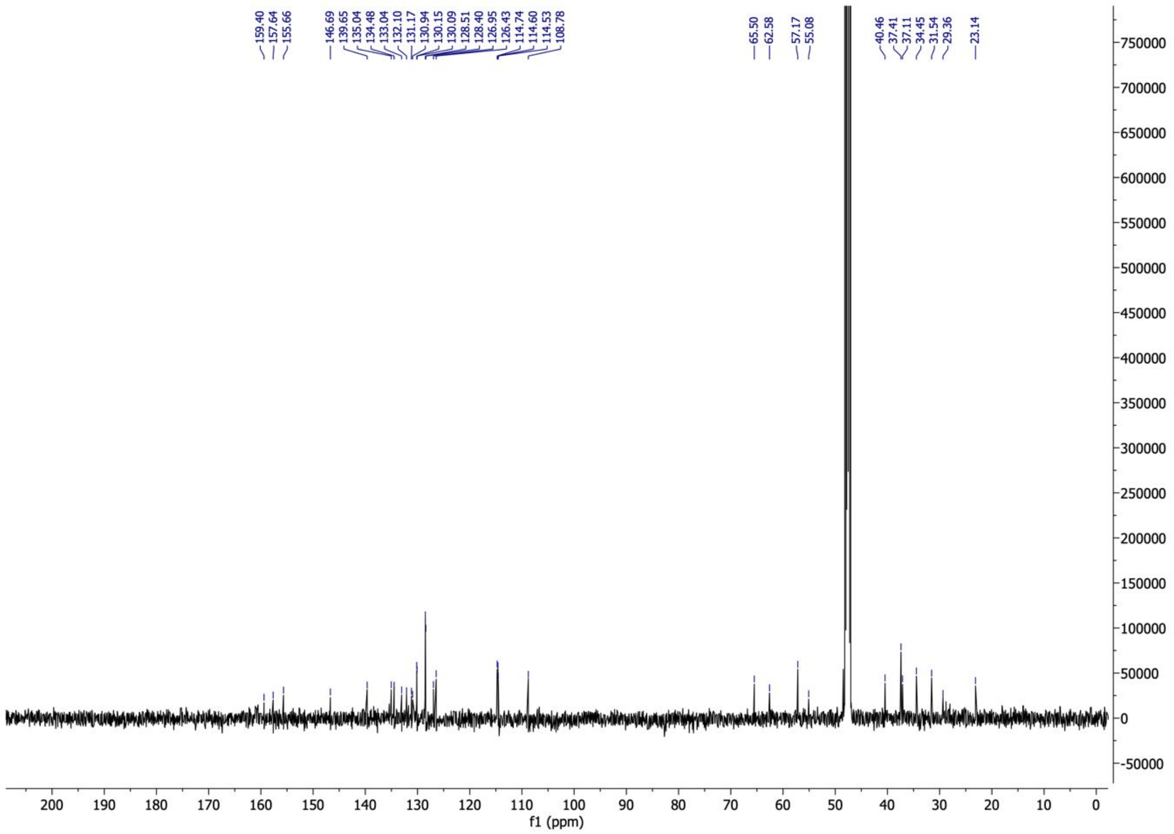

Bibliography

1. Madeira F, Pearce M, Tivey ARN, Basutkar P, Lee J, Edbali O, Madhusoodanan N, Kolesnikov A, Lopez R. Search and sequence analysis tools services from EMBL-EBI in 2022. *Nucleic Acids Res.* **2022**, *50* (W1), W276-W279. DOI: 10.1093/nar/gkac240.
2. Liebschner D, Afonine PV, Moriarty NW, Poon BK, Sobolev OV, Terwilliger TC, Adams PD. Polder maps: improving OMIT maps by excluding bulk solvent. *Acta Crystallogr D Struct Biol.* **2017**, *73* (Pt 2), 148-157. DOI: 10.1107/S2059798316018210.
